# Analysis of Differentially Expressed Genes Associated with Coronatine-Induced Laticifer Differentiation in the Rubber Tree by Subtractive Hybridization Suppression

**DOI:** 10.1371/journal.pone.0132070

**Published:** 2015-07-06

**Authors:** Shi-Xin Zhang, Shao-Hua Wu, Yue-Yi Chen, Wei-Min Tian

**Affiliations:** 1 College of Horticulture, Hainan University, Haikou, Hainan, 570228, China; 2 Key Laboratory of Biology and Genetic Resources of Rubber Tree, Ministry of Agriculture, State Key Laboratory Incubation Base for Cultivation and Physiology of Tropical Crops, Rubber Research Institute, CATAS, Danzhou, Hainan, 571737, China; USDA/ARS, UNITED STATES

## Abstract

The secondary laticifer in the secondary phloem is differentiated from the vascular cambia of the rubber tree (*Hevea brasiliensis* Muell. Arg.). The number of secondary laticifers is closely related to the rubber yield potential of *Hevea*. Pharmacological data show that jasmonic acid and its precursor linolenic acid are effective in inducing secondary laticifer differentiation in epicormic shoots of the rubber tree. In the present study, an experimental system of coronatine-induced laticifer differentiation was developed to perform SSH identification of genes with differential expression. A total of 528 positive clones were obtained by blue-white screening, of which 248 clones came from the forward SSH library while 280 clones came from the reverse SSH library. Approximately 215 of the 248 clones and 171 of the 280 clones contained cDNA inserts by colony PCR screening. A total of 286 of the 386 ESTs were detected to be differentially expressed by reverse northern blot and sequenced. Approximately 147 unigenes with an average length of 497 bp from the forward and 109 unigenes with an average length of 514 bp from the reverse SSH libraries were assembled and annotated. The unigenes were associated with the stress/defense response, plant hormone signal transduction and structure development. It is suggested that Ca^2+^ signal transduction and redox seem to be involved in differentiation, while PGA and EIF are associated with the division of cambium initials for COR-induced secondary laticifer differentiation in the rubber tree.

## Introduction

The rubber tree (*Hevea brasiliensis* Muell. Arg.) is the primary source of natural rubber (NR) in the world. The obtained NR is synthesized and stored in the secondary laticifer within the secondary phloem of trunk bark. The number of secondary laticifers in the trunk bark is closely related to the rubber yield potential of *Hevea* [1]. Although much effort has been focused on biological processes in the differentiated secondary laticifers [1–3], the molecular mechanism of the differentiation of the secondary laticifers from the cambia remains largely unknown due to the lack of an experimental system suitable for this purpose. It is possible that such an experimental system may be developed based on findings that the differentiation of the secondary laticifer in the epicormic shoot of the rubber tree could be induced by exogenous jasmonic acid (JA) and its precursor linolenic acid within 40 days [1]. The different fusiform initials of the vascular cambia differentiate the secondary laticifer cells in a synchronous manner and the induced laticifer cells can be easily detected by histochemical staining and distinguished from the primary laticifer cells [1].

Coronatine (COR) is a toxin isolated from *Pseudomonas syingae* pv. Atropurpurea [4]. It is composed of two components, the polyketide coronafacic acid (CFA) and coronamic acid (CMA) [5, 6]. COR structurally and functionally mimics the most active isoleucine conjugate of JA (+)-7-iso-JA-Ile (JA-Ile) and its activity is 100–10 000 higher than JA [7–11]. In the present study, an experimental system for the COR-induced secondary laticifer differentiation in the epicormic shoots of the rubber tree was developed to perform the molecular identification of genes related to secondary laticifer differentiation. By using the system, the suppression of subtractive cDNA libraries of COR-induced secondary laticifer differentiation was performed, and several differentially expressed genes were identified. The results provide some indicative cues for detecting the molecular events of secondary laticifer differentiation in the rubber tree.

## Materials and Methods

### Plant materials

**Fig 1:**
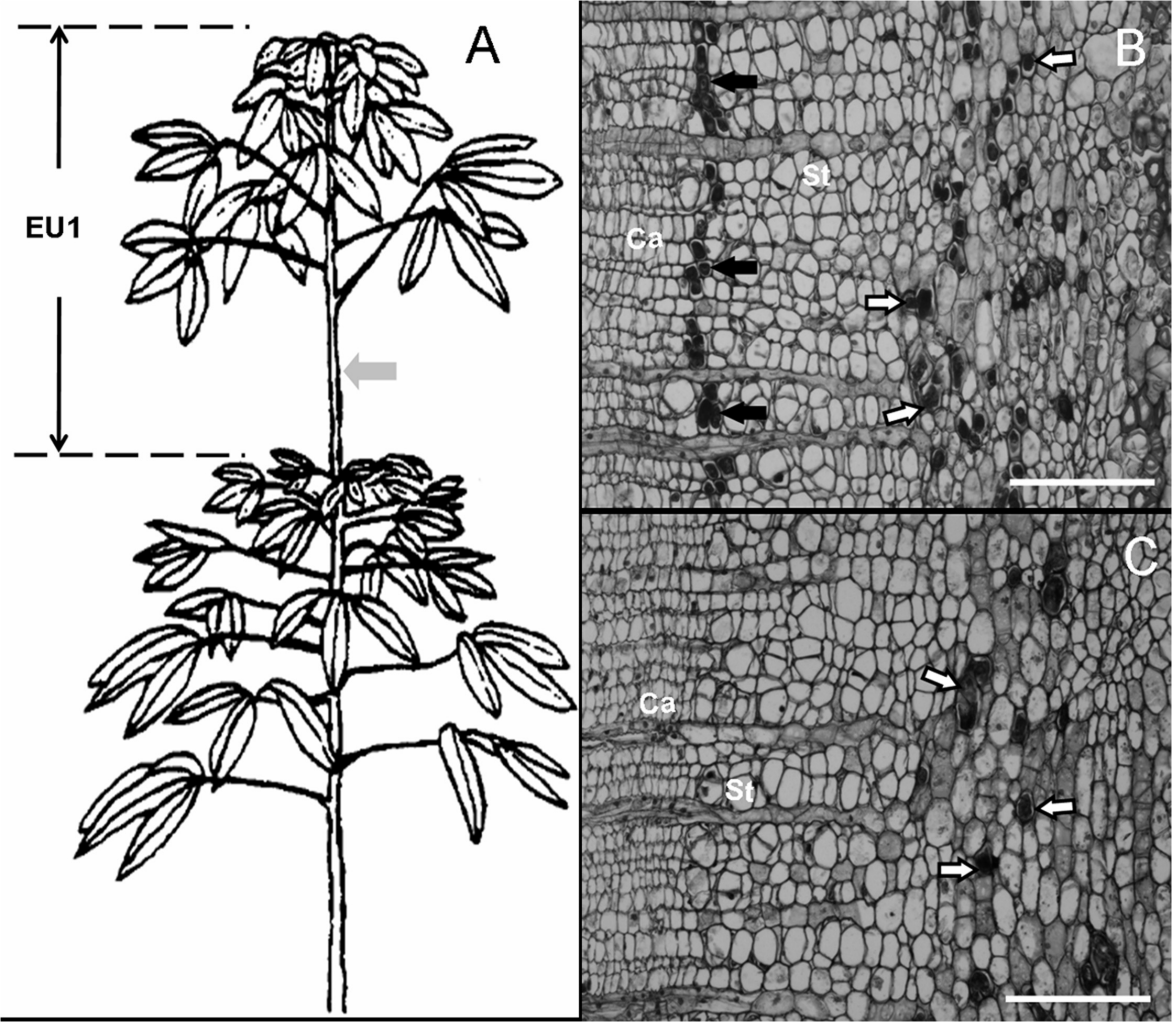
Diagram of *Hevea* epicormic shoots (A) and the cross-sections of bark (B-C) showing the sites of chemical application and secondary laticifer differentiation. The gray arrow in panel A indicates the site of chemical application and sampling. Panel B, treated with 20 μM coronatine (COR). Panel C, treated with water. EU, extension unit. White arrows show the primary laticifers. Black arrows show the secondary laticifers. Ca, cambium; St, sieve tube. Bars = 100 μm.

Plantlets of the rubber tree clone CATAS 7-33-97 budded on rootstocks were grown in the nursery of Rubber Research Institute of Chinese Academy of Tropical Agricultural Sciences (CATAS) on Hainan Island, P. R. China. The plantlets were pruned each year, and epicormic shoots were developed from the latent buds of the pruned stems. The epicormic shoots flush five to six times a year; therefore, such a shoot consists of a series of foliage clusters, separated by leafless lengths of stem. Each of these morphologically distinct growth increments represents a growth flush and is referred to as an extension unit (EU) [1] (Fig 1A). Fig 1A illustrates the sites of chemical application and sampling.

### Experimental treatments

When the epicormic shoots had developed more than two extension units, treatments were performed on EU1. The stem surface of 10 cm^2^ in the middle of the EU1 (Fig 1A) was scraped with a sharp razor to remove the epidermis cuticle and the part of the cortex. The wounded surface was immediately applied with 20 μM COR (Sigma, USA) and sterile water and wrapped with a polyethylene membrane. For SSH (Suppression subtractive hybridization) library construction, the treated sites were sampled 1 day, 2 days and 3 days after the treatments. The tissue samples containing vascular cambia were collected from the inside of the bark and the outside of the wood by scraping with an RNase-free sharp razor. To obtain enough tissue samples for poly(A)+ mRNA purification from total RNA, samples were collected from nine epicormic shoots at each time interval for each treatment. The tissue samples from the nine shoots were combined (ranging from 0.739 g to 1.146 g) to be used to extract total RNA. For light microscope observation, bark samples, including part of the xylem, were collected from three epicormic shoots 7 days after the treatments. For real-time PCR analysis, the treated sites were sampled half an hour (0.5 h), one hour (1 h), two hours (2 h), four hours (4 h), and eight hours (8 h) after treatments, in addition to sharing the samples used for SSH library construction. The vascular cambia-containing tissue samples were collected from nine epicormic shoots at each time interval. The samples from every three of the nine epicormic shoots were mixed to be used to extract total RNA, with three biological replicates of RNA samples for each time interval. The vascular cambia-containing tissue was collected from the inside of the bark and the outside of the wood by scraping with an RNase-free sharp razor.

### Light microscopy

To eliminate tannin-like substances that may be mistaken for the rubber inclusion in laticifer, bark samples were fixed in 80% ethanol for 24 h at room temperature, treated with iodine and bromine in glacial acetic acid [1], and embedded in paraffin after dehydration. Sections (12 μm in thickness) were cut with a microtome (Leica Microsystems Inc., Bannockburn, IL, Germany) and stained with fast green. The laticifers in sections could be recognized because the rubber in the laticifers was brown in color due to the iodine-bromine treatment [1].

### RNA isolation

Total RNA was extracted according to the method of Tian et al. [12]. The RNA quality was analyzed by the ultraviolet spectrophotometer, and the quantity was determined by the Thermo Gene Company Limited ND2000 (Thermo Fisher Scientific, Inc., Waltham, MA, USA) (S1 Table).

### Construction and screening of SSH cDNA libraries

Poly(A)+ mRNA was purified from total RNA with an Oligotex mRNA Mini Kit (QIAGEN, Valencia, CA, USA). SSH was performed using the PCR-Select cDNA subtraction kit (Clontech Laboratories, Inc., Mountain View, CA, USA) according to the manufacturer's protocol and the methods of Diatchenko et al. [13]. For forward subtraction, cDNA from the COR-treated samples was used as a “tester,” and the sterile water-treated sample was used as a “driver.” For reverse subtraction, cDNA from the sterile water-treated samples was used as a “tester” and the COR-treated sample was used as a “driver.” The second PCR amplification products of the subtracted cDNAs were cloned into pMD 18-T vectors using the TA cloning kit (Takara, Dalian, China). The subtraction efficiency was evaluated by PCR with primers for the rubber tree 18s rRNA gene (the primers as 5’- GGTCGCAAGGCTGAAACT-3’ and 5’-ACGGGCGGTGTGTACAAA-3’). The PCR products from the subtracted samples were linked into the pGEM-T easy vector (Promega Corporation, Madison, WI, USA) and then transferred into *E*. *coli* competent cells JM109 (Promega Corporation, Madison, WI, USA) to generate SSH libraries. The transformants were planted on LB agar plates containing 100 μg/ml ampicillin, 40 μg/ml 5-Bromo-4-chloro-3-indolyl β-D-galactopyranoside (X-Gal) and 1 mM isopropyl-beta-D-thiogalactopyranoside (IPTG) for blue-white screening. All the chemicals were purchased from Sangon Biotech (Shanghai, China). The cDNA inserts of the white colonies were checked by colony PCR with M13 forward and reverse primers. The clones that yielded a single PCR product were selected for the next analysis.

### Reverse Northern blot analysis

To validate the differential ESTs between COR and water treatment, reverse northern blot was performed with DIG-labeled cDNA probes. The six cDNA samples were obtained by reverse transcription of the unsubtracted total RNA samples from cambia-containing tissues upon both COR treatment and water treatment for 1 day, 2 days and 3 days. The 1 μg of each cDNA sample was labeled and detected using the DIG DNA Labeling and Detection Kit (Roche Diagnostics GmbH, Mannheim, Germany). Approximately 1 μl of each colony PCR product was spotted by hand onto a sheet of nylon membrane (Roche Diagnostic Corporation, Germany) and then linked by UV-cross (1.2 J/cm^2^), dried at 50°C for 10 min in an oven, and stored at 4°C. Hybridization was performed in a hybridizer (*HM-4000 Multidizer*, Ultra-Violet Products Ltd., Cambridge, UK) at 42°C for 8 hours. After hybridization, the sheet was washed twice in 2× SSC containing 0.1% SDS at 25°C for 5 min followed by washing twice in 0.5× SSC containing 0.1% SDS at 65°C for 15 min. The sheet was subjected to immunological detection with anti-digoxigenin-AP conjugate and the premixed stock solution of NBT/BCIP supplied in the DIG DNA Labeling and Detection Kit, according to the manufacturer’s instructions.

### DNA sequencing and analysis

Raw sequence trace files were performed by DNA Sequencing Analysis Software v5.2 (Applied Biosystems) to obtain base-calling with quality scores. The low quality sequences (quality score <55) and the short sequences (< 100 bp) were given up. The vector and adaptor sequences were detected by DNAMAN 7.0 software and removed. All unique genes were searched against the NCBI database with the basic local alignment search tool (BLASTX and BLASTN) (http://blast.ncbi.nlm.nih.gov). The functional categories of all unique genes were performed according to Gene Ontology [14].

### Real-time PCR analysis

Real-time PCR analysis was performed to validate the results of the SSH data and to monitor the differential expression pattern of selected unigenes with *HbACTIN* (NCBI accession number: JF270598) [15] and *HbRH8* (NCBI accession number: HQ323244) [16] for double housekeeping gene analysis [17]. The primer sequences were listed in S2 Table. The real-time PCR reactions and correct analysis were performed with a CFX96/384 touch real-time PCR detection system (Bio-Rad Labratories Inc, Ca, USA). The stability value of the housekeeping genes was assessed. The mean CV was 0.1455 (requirement, <0.25), and the mean M value was 0.4172 (requirement, <0.5). The amplification efficiency of the tested genes was similar to that of the reference genes (S2 Table). Real-time PCR was performed by using 100 ng first-strand cDNA, 10 pmol forward and reverse primers and the TransStart Tip Green qPCR SuperMix (a SYBR green mix, TransGen Biotech, Beijing, China) in a 20-μl reaction mixture, according to the manufacturer's protocol. Amplification was carried out at 95°C for 5 min, followed by 40 cycles (95°C for 10 sec, Tm for 20 sec, 72°C for 10 sec). The relative expression values were calculated from three biological replicates using a modified 2-^ΔΔ^CT method [18]. The single factor variance analysis of the real-time PCR data was performed with SPSS 20 software.

## Results

### Effect of COR on inducing secondary laticifer differentiation

Under natural conditions, there were no secondary laticifers in the bark tissues of the stem of EU1, although the primary laticifers had developed (Fig 1A–1C). Upon 20 μM COR treatment for 7 days, a row of the secondary laticifers was observed in the secondary phloem close to the cambia region (Fig 1B). As a control, water had no effect on inducing secondary laticifer differentiation (Fig 1C). Previous data showed that exogenous jasmonic acid and its precursor linolenic acid could induce secondary laticifer differentiation within 40 days [1]. The present study demonstrated that COR could mimic the role of exogenous jasmonic acid in inducing secondary laticifer differentiation and be even more effective. This experimental system is suitable for the identification of the differentially expressed genes associated with secondary laticifer differentiation.

### Construction of SSH libraries and reverse northern blot analysis

Subtractive efficiency is vital for the successful construction of SSH libraries. In this study, we used the rubber tree 18s rRNA gene (a housekeeping gene) to evaluate the subtractive efficiency of the SSH libraries. The 18s rRNA gene PCR products appeared to be detectable after 22- and 28-amplification cycles with unsubtracted and subtracted cDNAs as PCR templates, respectively (S1 Fig). It was shown that the samples were effectively subtracted and genes with differential expression were enriched in the two SSH libraries.

**Fig 2:**
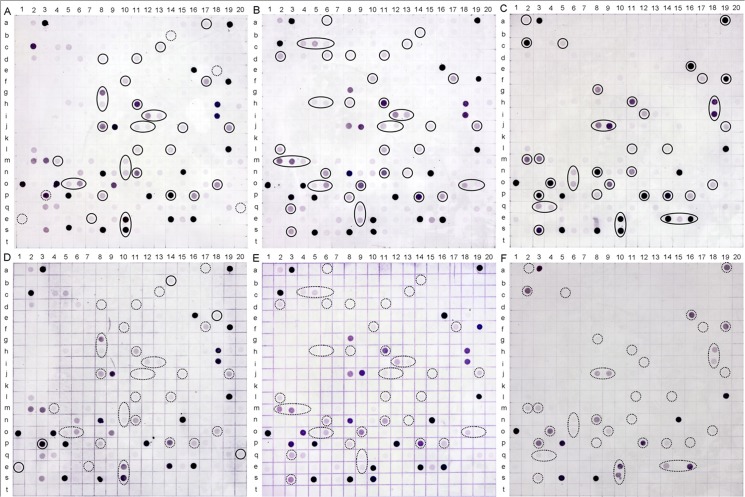
Reverse northern blot analysis of differentially expressed ESTs in the forward and reverse SSH libraries. Panel A, B and C, hybridization with unsubtracted cDNA probes from samples upon COR treatment for 1 day (A), 2 days (B) and 3 days (C). Panels D, E and F, hybridization with unsubtracted cDNA probes from samples upon water treatment for 1 day (D), 2 days (E) and 3 days (F). a to k, ESTs from the forward SSH library. i to t, ESTs from the reverse SSH library. The strong and corresponding weak hybridization signals were indicated with solid rings and dotted rings, respectively.

A total of 528 positive clones were obtained by blue-white screening, of which 248 clones came from the forward SSH library, while 280 clones came from the reverse SSH library. Approximately 215 of the 248 clones and 171 of the 280 clones contained cDNA inserts according to colony PCR screening. The differential expression of all the ESTs from the 386 positive clones (215 and 171 clones) was analyzed by reverse northern blot. The sheet containing 215 ESTs (Fig 2, line a-k) and 171 ESTs (Fig 2, line j-t) was hybridized with labeled cDNA probes from samples treated with COR (Fig 2A–2C) and water (Fig 2D–2F) for 1 day (Fig 2A and 2D), 2 days (Fig 2B and 2E) and 3 days (Fig 2C and 2F). A difference in dot color of the duplicates between hybridization with COR and water treatment at the same time interval was observed. A few of the differentially expressed spots were marked with real rings for strong dots and dotted rings for the corresponding weak ones (Fig 2).

### Sequence assembly and unigene annotation

A total of 286 differentially expressed ESTs were selected for sequencing. After the ESTs with a length of less than 100 bp were excluded, there were 143 and 93 unigenes assembled from the ESTs in the forward and reverse SSH libraries, respectively. All the 236 unigenes displayed at least one significant alignment with an existing gene in the NCBI database (quality score ≥ 55) based on BLASTX and BLASTN analysis (S3 Table and S4 Table). All EST sequences were provided in the supporting information (S7 Table).

**Fig 3:**
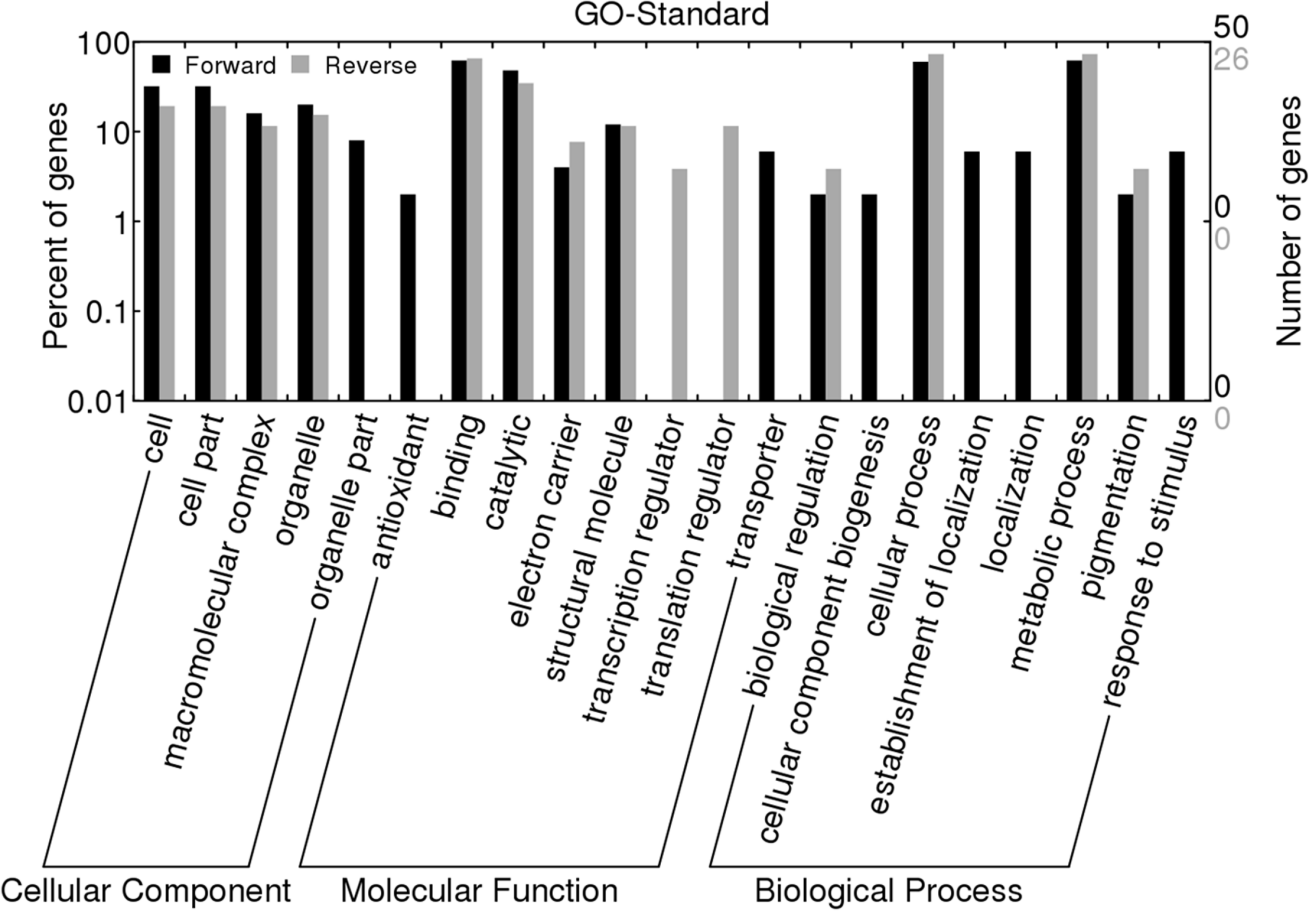
The GO standard analysis of 286 genes from the forward and reverse SSH libraries by BGI WEGO.

Gene Ontology [14] conventions were used to provide descriptions of the gene products associated with molecular functions, cellular components and biological processes [19]. The functional classification of the 236 unigenes was performed by the GO convention (http://geneontology.org/). Fig 3 was drawn in Web Gene Ontology Annotation Plotting (BGI WEGO) (http://wego.genomics.org.cn/cgi-bin/wego/index.pl) to show the percentages of the subtractive unigenes in the three GO categories. (1) Approximately 54 and 17 unigenes were classified into 5 categories of the cellular component in the forward and reverse SSH libraries. Among them, unigenes encoding for cytochrome f, cytochrome b6 and ORF 143 were only present in the forward SSH library. (2) Approximately 67 and 35 unigenes were classified into 8 categories of molecular functions. The largest proportion of molecular functions were categorized by binding (31 and 17 unigenes) and catalytic activity (24 and 9 unigenes). Interestingly, antioxidant activity (1 unigene: predicted protein) and transporter activity (3 unigenes: vesicle docking protein P115, pyrophosphate-energized membrane proton pump 3 and PPase) were only noted in the forward SSH library; however, transcription regulator activity (1 unigene: *Hevea brasiliensis* microsatellite Hbtnr-23) and translation regulator activity (3 unigenes were all translation initiation factor, EIF) were annotated in the reverse SSH library. (3) Approximately 73 and 44 unigenes were classified into 8 categories of biological processes in the forward and reverse SSH libraries. Cellular processes (30 and 20 unigenes) and metabolic processes (31 and 20 unigenes) were the most frequently represented cellular components. Additionally, the 4 categories of biological processes: cellular component biogenesis (1 unigene: auxin-responsive protein IAA19), establishment of localization (3 unigenes: PPase, vesicle docking protein P115 and pyrophosphate-energized membrane proton pump 3), localization (3 unigenes: PPase, vesicle docking protein P115 and pyrophosphate-energized membrane proton pump 3) and response to stimulus (3 unigenes: predicted protein and HSP80) only appeared in the forward SSH library. Overall, the results showed that an obvious difference in several parts of the three GO categories existed between unigenes in the forward and reverse SSH libraries.

KEGG annotation provided an alternative functional annotation of genes according to their associated biochemical pathways based on sequence similarity searches against the KEGG database (http://www.genome.jp/kegg/pathway.html). The 236 unigenes were also assigned to KEGG analysis. The unigenes mapped to the KEGG pathways were shown in S5 Table. Among the unigenes in the two libraries, 72 corresponded to metabolism, 53 mapped to genetic information processing, 3 were categorized into environmental information processing, 1 belonged to cellular processes, and 15 were classified as organismal systems. The pathway for the biosynthesis of other secondary metabolites, nucleotide metabolism, folding, sorting, degradation and translation were most often mapped for unigenes in the forward SSH library, while translation was most often mapped for unigenes in the reverse library (S5 Table). Among the KEGG pathways, environmental information processing only appeared in the forward library. Three unigenes (indole-3-acetic acid-induced protein ARG7, glutamine synthetase and 40S ribosomal protein S6) were mapped into the processing. Among the sub-pathways of the KEGG pathways, unigenes mediating lipid metabolism, signal transduction, environmental adaptation, the immune system and the nervous system only appeared in the forward library (S5 Table).

### Analysis of the differential expression pattern of several unigenes by real-time PCR

Through experimental morphology experiments, we found that cambium cell division was in progress 3 days after MeJA or mechanical wounding treatment [20]. It is reasonable to conclude that the genes differentially expressed within 3 days of COR treatment may be associated with secondary laticifer differentiation. Ten unigenes were selected from the forward and reverse SSH libraries to validate the reverse northern blot data and monitor expression patterns within 3 days of COR and water treatments by real-time PCR. These unigenes were related to signal transduction, metabolism and energy, RB, unknown function, stress/defense response, transcription and post-transcription, protein metabolism, transporter and cell biogenesis on the basis of GO and KEGG analyses. The specific real-time PCR primers of these 20 genes are listed in S2 Table. Most of the expression profiles of the 20 genes at the corresponding time intervals were consistent with the results of reverse northern blots.

**Fig 4:**
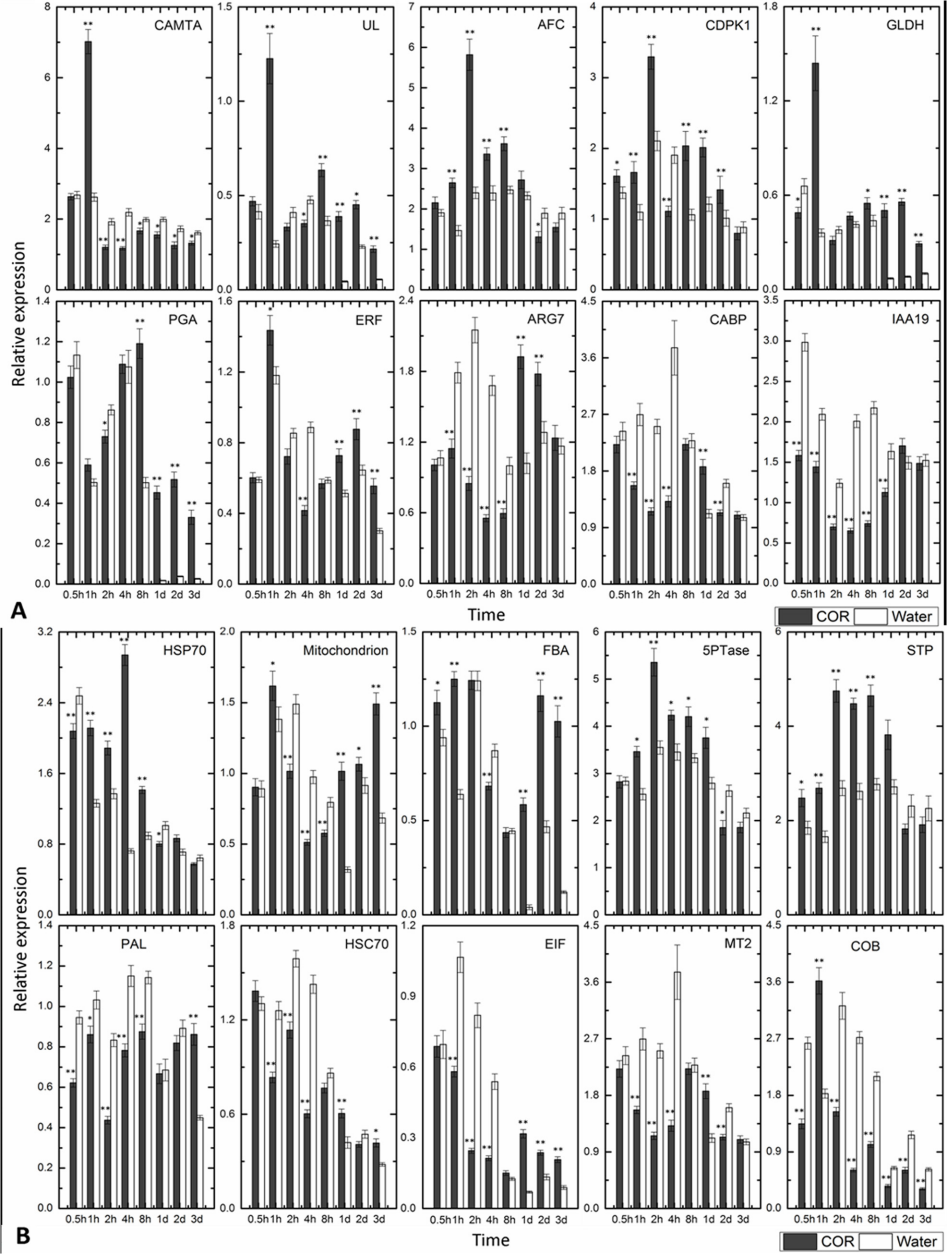
Expression pattern of all 10 unigenes from the forward (A) and reverse (B) SSH library by real-time PCR. Shoots were treated with 20 μM COR and water. Cambia-containing tissues were collected half an hour (0.5 h), one hour (1 h), two hours (2 h), four hours (4 h), eight hours (8 h), one day (1 d), two days (2 d) and three days (3 d) after treatments. The relative expression was normalized to the housekeeping genes of *HbACTIN* and *HbRH8*. The data were shown as averages ± SE. *, significant difference (*P* < 0.05); **, very significant difference (*P* < 0.01).

The expression pattern of 10 unigenes from the forward and reverse SSH library was shown in Fig 4A and 4B, respectively. The Figs were drawn by the software OriginLab 9.0 (OriginLab Corporation. MA. USA). The expression pattern of most of the unigenes from the forward SSH library showed a sharp contrast between the COR and water treatments (Fig 4A). The unigenes encoding calmodulin-binding transcription activator (CAMTA), ubiquitin 11-like (UL), calcium-dependent protein kinase 1 gene (CDPK1), N-acetyl-farnesylcysteine (AFC) and galactono-1,4-lactone dehydrogenase (GLDH) were rapidly up-regulated at 1 h (CAMTA, UL, GLDH) and 2 h (CDPK1, AFC), and thereafter, they were down-regulated and remained relatively stable upon COR treatment (Fig 4A). It was noted that the transcript level of all of the four unigenes (UL, AFC, GLDH and CDPK1) was still significantly higher upon COR treatment than that upon water treatment at the late stage (from 8 h to 1d or 3 d). Only at the late stage (from 8 h to 3 d) was the transcript level of the unigene encoding for polygalacturonase (PGA) upon COR treatment significantly higher than that upon water treatment. The reverse was the case for the unigene encoding for CAMTA. By contrast, the transcript level of unigenes encoding indole-3-acetic acid-induced protein (ARG7) and calmodulin binding protein (CABP) at the early stage (from 1 h to 4 h) and the unigene encoding auxin-responsive protein IAA19 (IAA19) from 0.5 h to 1 d was significantly lower upon COR treatment than that upon water treatment. However, the expression patterns of most of the unigenes from the reverse SSH library were roughly similar between COR and water treatments (Fig 4B). Nevertheless, compared with the water treatment, the transcript levels of the unigenes encoding fructose-bisphosphate aldolase (FBA), phosphoinositide 5-phosphatase (5TPase) and sugar transporter (STP) were significantly higher while those encoding phenylalanine ammonia-lyase 3 (PAL), metallothionein (MT2) and mitochondrial cytochrome b (COB) were significantly lower at most of the time intervals after COR treatment. The transcript levels of the unigene encoding translation initiation factor-like protein (EIF) were significantly lower at the early stage (from 1 h to 4 h) and higher at the late stage (from 1 d to 3 d) after COR treatment.

## Discussion

In previous studies, we have found that jasmonic acid (JA) and its precursor linolenic acid have an effect on inducing secondary laticifer differentiation in the stem of epicormic shoots of the rubber tree [20]. This finding implies that jasmonate signaling plays a pivortal role in regulating the differentiation of the secondary laticifer, a defense structure, in the rubber tree. As coranatine (COR) can structurally and functionally mimic the most active isoleucine conjugate of JA, (+)-7-iso-JA-Ile (JA-Ile) [7–11], we developed an experimental system of COR-induced secondary laticifer differentiation to trace the molecular events associated with secondary laticifer differentiation. Scraping the stem surface is necessary for COR-induced secondary laticifer differentiation (data not shown). Accordingly, the application of COR on the intact surface of the stem of epicormic shoots has a much smaller effect on gene expression than that of COR being applied on the wounded surface (S2 Fig). Although the effect of scraping *per se* on gene expression is stronger than that of COR applied on the intact surface, this effect is much less than that of COR being applied on the wounded surface (S2 Fig). It is noted that water has an obvious influence on gene expression (S3 Fig), which is consistent with the evident differences in the expression of most of the selected twenty genes among time intervals upon water treatment (Fig 4). Moreover, the effect of water being applied on the wounded surface is stronger than that of scraping *per se* (S3 Fig). Although water influences gene expression in a positive or negative manner, it does not induce secondary laticifer differentiation (Fig 1C). It seems reasonable that genes with similar expression patterns for COR and water treatments are unlikely to be related to COR-induced secondary laticifer differentiation.

In the present study, COR was demonstrated to be very effective in inducing secondary laticifer differentiation, and hundreds of differentially expressed genes were detected by screening SSH libraries. The dissection of the molecular regulating network of COR-induced secondary laticifer based on these differentially expressed unigenes is impossible at present because no data are available from plant models. Nevertheless, unigenes that exhibit reverse expression patterns for the COR and water treatments and are related to jasmonate signaling, stress response and development should be considered to be associated with COR-induced secondary laticifer differentiation.

It is interesting that several differentially expressed unigenes are stress/defense-related and associated with jasmonate signaling and development (S6 Table). These unigenes are clustered into several biological processes, of which Ca^2+^ signal transduction and redox seem to be closely related to COR-induced secondary laticifer differentiation. In plants, intracellular Ca^2+^ levels are modulated in response to abiotic and biotic stresses, and specific calcium signatures can be transduced into downstream effects by different calcium sensors [21, 22]. Ca^2+^/CaM has long been considered a crucial component in the plant defense signaling pathway [23]. In *Arabidopsis*, ABA and MeJA activate slow anion channels and Ca^2+^ permeable cation channels in the plasma membrane of wild-type guard cell protoplasts to mediate various guard cell defense responses [24]. A calmodulin-like protein, CML42, regulates plant defense and JA signaling for increased herbivory [25], and six *AtCAMATA*s differentially respond to a variety of external signals, such as cold, wounding and drought [26, 27]. Moreover, CAMTA can also regulate the development and ripening of tomato fruit. CDPKs are critical in modulating JA homeostasis [28]. Silencing calcium-dependent protein kinases *NaCDPK4* and *NaCDPK5* results in wound- and herbivory-induced JA accumulation and affects plant resistance against insects in a JA- and JA signaling-dependent manner in *Nicotiana attenuate* [28]. CDPK1 is also responsible for cold, drought and wound responses in plants [29] [30] and is involved in regulating root development in *Medicago truncatula* [31] and *Sergey Ivashuta* [30]. In the present study, unigenes encoding CAMTA, CDPK1 and CABP, the components of Ca^2+^ signal transduction, are differentially expressed between the COR and water treatments. It is indicative that Ca^2+^ signal transduction may be involved in regulating secondary laticifer differentiation. Another cue for the regulation of secondary laticifer differentiation may be redox. The unigenes encoding COB, GLDH, MT2 and myoinositol polyphosphate 5-phosphatases (5PTases, EC3.1.3.56) are differentially expressed between the COR and water treatments. These unigenes are associated with the regulation of redox as well as other biological processes. *Arabidopsis thaliana* has 15 genes encoding 5PTases. The 5PTase7 can regulate the production of reactive oxygen species and salt tolerance in [14]. The 5PTase12 and 5PTase13 control Ins (1, 4, 5) P3/Ca^2+^ for maintaining pollen dormancy and regulating the early germination of pollen [32], and 5PTase13 interacted with SnRK1, linking inositol, sugar, and stress signaling [5]. In the mitochondria of eukaryotes, cytochrome b (COB) is a component of respiratory chain complex III (EC 1.10.2.2) [33], acts in electron transfer and can regulate the balance of active oxygen in plants [34]. GLDH is an FAD-containing oxidoreductase that catalyzes the terminal step of the Smirnoff–Wheeler pathway of vitamin C (L–ascorbate) biosynthesis in plants [35, 36]. L-ascorbic acid participates in the detoxification of ROS (Reactive oxygen species) produced from processes such as oxidative metabolism and photosynthesis [37, 38]. The MTs are implicated in a range of physiological processes, including the role of scavenging ROS [39, 40] [41].

In addition, unigenes encoding indole-3-acetic acid-induced protein (ARG7) and auxin-responsive protein IAA19 (IAA19) are significantly down-regulated upon COR treatment at early stages, suggesting that IAA signaling may antagonize the effect of COR on inducing secondary laticifer differentiation.

The differentiation of fusiform initials is followed by cell division. Polygalacturonases (PGA) are believed to be responsible for various biological processes, such as seed germination, organ abscission, pod and anther dehiscence, pollen grain maturation, fruit softening and decay, xylem cell formation, and pollen tube growth [42]. EIF is related to stress/defense response and development. In *Arabidopsis thaliana*, *AtEIF5A3* can enhance thermotolerance and oxidative and osmotic stress resistance [43], while *AtEIF3f* is required for pollen germination and embryogenesis [44]. It is noted that the cell division-related genes PGA and EIF are substantially up-regulated upon COR treatment from 1 day to 3 days, which is consistent with the occurrence of cambium initial division 3 days after mechanical wounding [20].

## Conclusion

With the aid of a suitable experimental system, unigenes differentially expressed in response to COR treatment were detected for the first time. Ca^2+^ signal transduction and redox seems to be involved in differentiation, while PGA and EIF are associated with the division of cambium initials during COR-induced secondary laticifer differentiation. As the functional annotation of most of unigenes refers to the data from plant species without laticifer, it is still a challenge to identify laticifer differentiation-related genes in the rubber tree.

## Supporting Information

S1 Fig Agrose gel electrophoresis profiles of the PCR products of 18s rRNA, showing subtractive efficiency. The figure showed a light band after 25 cycles of PCR by using unsubtracted sample (31 cycles of subtracted), the results show that 18sRNA is effective for the test of subtracted efficiency.
(TIF)

S2 Fig Effect of scraping on the expression of *HSP70*. The figure provided the comparison of the HSP70 (heat shock protein 70) transcripts level in the cambia-containing tissues from the intact stem of epcormic shoots without any treatments, all of the application of COR on the intact surface, scraping per se, and application of COR on the wounded surface of the stem of epicormic shoots influence the expression of HSP70. The effect of scraping on the gene expression was stronger than that of COR being applied on the intact surface while much less than that of COR being applied on the wounded surface.
(TIF)

S3 Fig Effect of water on the expression of *HSC70*. The figure provided the comparison of the HSC70 (heat shock cognate 70-interacting protein) transcripts level in the cambia-containing tissues from the intact stem of epcormic shoots without any treatments, all of the application of water on the intact surface, scraping per se, and application of water on the wounded surface of the stem of epicormic shoots influence the expression of HSC70. The effect of scraping on the gene expression was stronger than that of water being applied on the intact surface while less than that of water being applied on the wounded surface.
(TIF)

S1 Table RNA samples. The table included the information of RNA samples: treatment, time interval, t260/280, concentration and usage.
(XLS)

S2 Table Primers and amplification efficiency of genes for real-time PCR. The table presented the information of real-time PCR: genes names, primers, optimal annealing temperature (Tm), amplification efficiency and correlation coefficient(R^2^).
(XLS)

S3 Table The identification of non-redundant clones from the forward SSH library. The table provided the information of unigenes in forward SSH library, including putative functional classification, unigene number and cDNA length, and the information of matched gene accession No., E-value, putative identify and species from blastx or blastn analysis.
(XLS)

S4 Table The identification of non-redundant clones from the reverse SSH library. The table provided the information of unigenes in reverse SSH library, including putative functional classification, unigene number and cDNA length, and the information of matched gene accession No., E-value, putative identify and species by blastx or blastn analysis.
(XLS)

S5 Table Number of unigenes mapped to KEGG pathways. The table listed the numbers of unigenes and names of the mapped KEGG pathways.
(XLS)

S6 Table Functions of unigenes mediating JA signaling, stress/defense and the development. The function annotation was based on available literatures which were attached in the file.
(DOCX)

S7 Table The sequences of all ESTs in the forward and reverse SSH libraries. The table included a total of 256 ESTs sequences that the vector and adaptor sequences were detected, of which 147 ESTs came from the forward SSH library, while 109 ESTs came from the reverse SSH library.
(DOCX)
