# Supplementary figures and images for: Analysis of Differentially Expressed Genes Associated with Coronatine-Induced Laticifer Differentiation in the Rubber Tree by Subtractive Hybridization Suppression
Source: PLoS One. 2015 Jul 6;10(7):e0132070. doi: 10.1371/journal.pone.0132070 (PMC4493031; doi:10.1371/journal.pone.0132070)

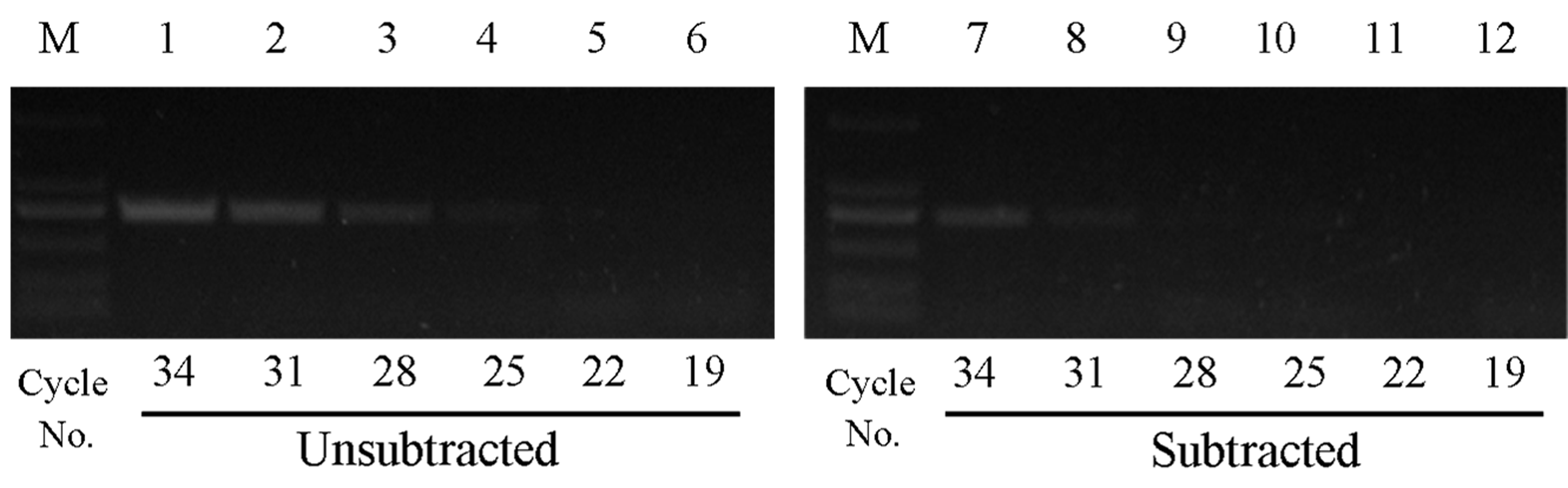

Supplement: S1 Fig — The figure showed a light band after 25 cycles of PCR by using unsubtracted sample (31 cycles of subtracted), the results show that 18sRNA is effective for the test of subtracted efficiency. (TIF) [file pone.0132070.s001.tif]

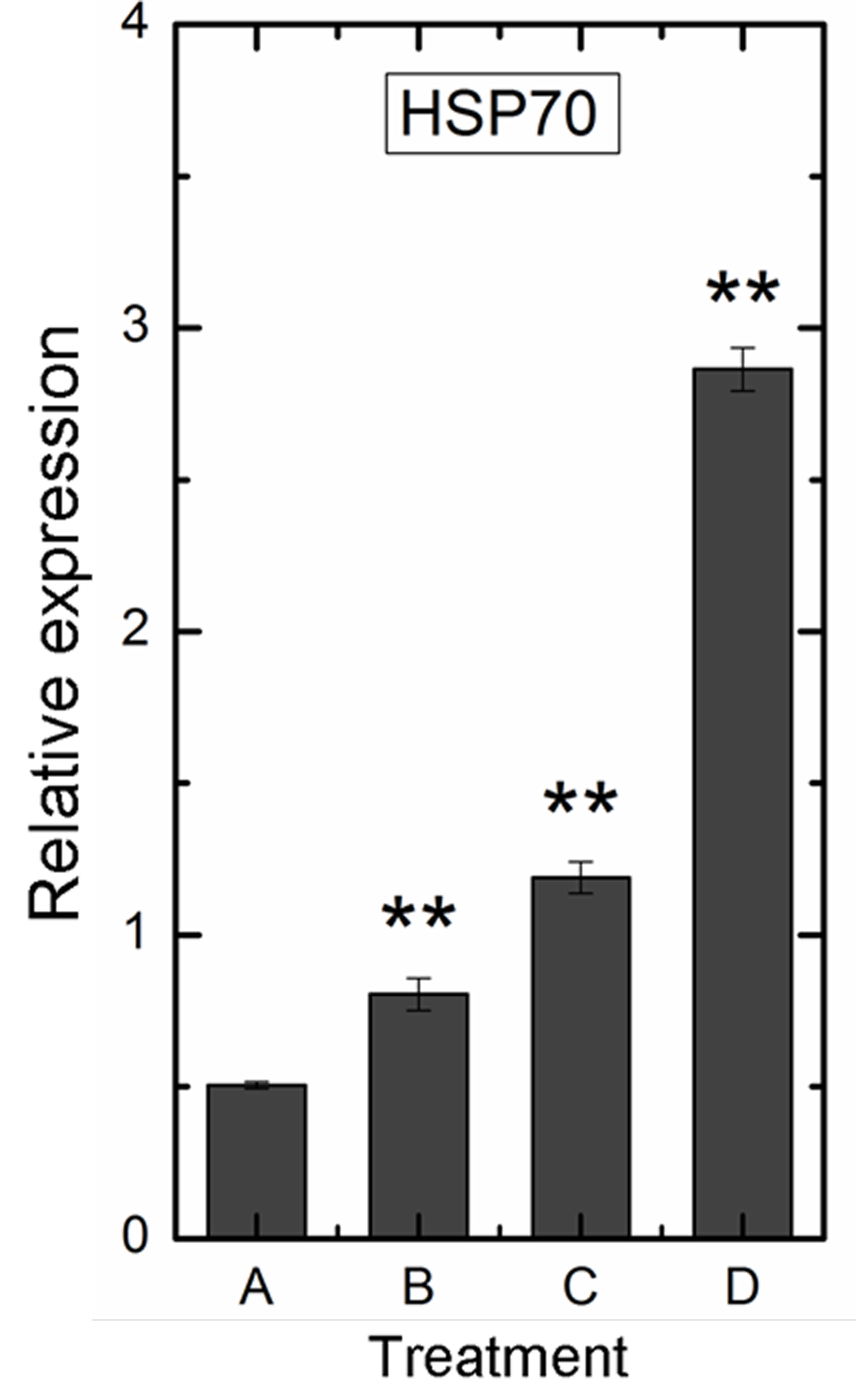

Supplement: S2 Fig — The figure provided the comparison of the HSP70 (heat shock protein 70) transcripts level in the cambia-containing tissues from the intact stem of epcormic shoots without any treatments, all of the application of COR on the intact surface, scraping per se, and application of COR on the wounded surface of the stem of epicormic shoots influence the expression of HSP70. The effect of scraping on the gene expression was stronger than that of COR being applied on the intact surface while much less than that of COR being applied on the wounded surface. (TIF) [file pone.0132070.s002.tif]

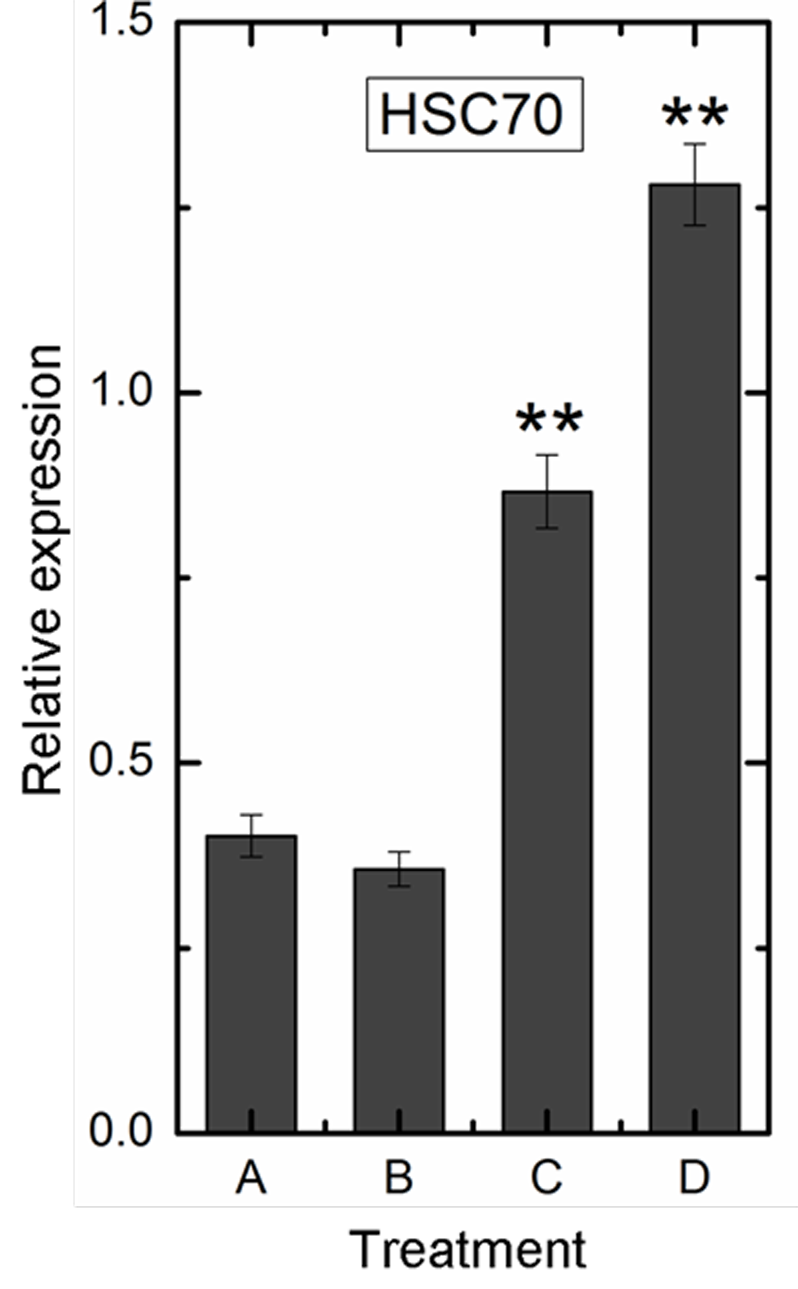

Supplement: S3 Fig — The figure provided the comparison of the HSC70 (heat shock cognate 70-interacting protein) transcripts level in the cambia-containing tissues from the intact stem of epcormic shoots without any treatments, all of the application of water on the intact surface, scraping per se, and application of water on the wounded surface of the stem of epicormic shoots influence the expression of HSC70. The effect of scraping on the gene expression was stronger than that of water being applied on the intact surface while less than that of water being applied on the wounded surface. (TIF) [file pone.0132070.s003.tif]
