# Supplementary material for: Analysis of Differentially Expressed Genes Associated with Coronatine-Induced Laticifer Differentiation in the Rubber Tree by Subtractive Hybridization Suppression
Source: PLoS One. 2015 Jul 6;10(7):e0132070. doi: 10.1371/journal.pone.0132070 (PMC4493031; doi:10.1371/journal.pone.0132070)
Supplement: S6 Table — The function annotation was based on available literatures which were attached in the file. (DOCX) [file pone.0132070.s009.docx]

**S6 Table. Functions of unigenes mediating JA signaling, stress/defense and the development**

| Gene name | | Function | Reference |
| --- | --- | --- | --- |
| SSH-A1(bHLH1 transcription factor) | | Regulation of laticifer differentiation and latex biosynthesis adjusted by JA | GenBank: FJ839963.1, [[1](#_ENREF_1)] |
| SSH-A10(Ethylene-Response Factors, DREB1p) | | JA and ET signal pathway, drought and low temperature response | [[2](#_ENREF_2)] |
| SSH-A11(Plasma membrane intrinsic protein, PIP1, 2) | | Transport and water stress | [[3](#_ENREF_3)] |
| SSH-A13(Ethylene response factor, ERF) | | Regulated to JA and ET signal pathway | [[4](#_ENREF_4)] |
| SSH-A19(RNA polymerase beta subunit) | | JA–mediated defenses that protect against fungi are related to pol V | [[5](#_ENREF_5)] |
| SSH-A32(Tau class glutathione transferase, GSTU52) | | Salicylic acid, jasmonic acid and the auxin K-naphthalene acetic acid triggered osgstu4 and osgstu3 expression. | [[6](#_ENREF_6)] |
| SSH-A33(TIR-NBS-LRR type disease resistance protein) | | JA induced detectable expression of the five NBS-LRR-encoding genes and one TIR-NBS-encoding gene | [[7](#_ENREF_7)] |
| SSH-A63(Calmodulin-binding transcription activator, CAMTA) | | SR1, a calmodulin-binding transcription factor, modulates plant defense and ethylene-induced senescence by directly regulating NDR1 and EIN3 in JA and ET signal pathway | [[8](#_ENREF_8)] |
| SSH-A64(Calcium-dependent protein kinase 1 gene, CDPK1) | | NaCDPK4 and NaCDPK5 have a role in the early steps of JA biosynthesis | [[9](#_ENREF_9)] |
| SSH-B5(Phosphoinositide 5-phosphatase, Putative, 5TPase) | | At5PTase11 gene is regulated by jasmonic acid, suggesting a role for phosphoinositide action in JA signal transduction pathways. | [[10](#_ENREF_10)] |
| SSH-B19(Phenylalanine ammonia-lyase 3, PAL) | | MeJA up-regulation of phenylalanine ammonia-lyase (PAL5) in tomato | [[11](#_ENREF_11)] |
| SSH-B35(Metallothionein, MT2) | | Exogenous jasmonic acid (JA) type-2 metallothionein gene (*AmMT2*) expression in seedlings of *Avicennia marina* | [[12](#_ENREF_12)] |
| SSH-B45(MatR gene product, Mitochondrion) | The Mutants *nMat2* plants demonstrated growth to flowering defect phenotypes in Arabidopsis | [[13](#_ENREF_13)] |  |
| SSH-A2(Vesicle docking protein P115) | P115 promotes growth of gastric cancer cell | [[14](#_ENREF_14)] |  |
| SSH-A3(Secondary cell wall-related glycosyltransferase family 8) | Cell wall biosynthesis | [[15](#_ENREF_15)] |  |
| SSH-A5(Auxin-responsive protein IAA19, Putative, IAA19) | lateral root formation and IAA singal pathway | [[16](#_ENREF_16)] |  |
| SSH-A14(Fructokinase, Putative) | Fructokinase as a hexose receptors and signaling molecules influence the life cycle to regulate metabolism and develpment in plant | [[17](#_ENREF_17),[18](#_ENREF_18)] |  |
| SSH-A24(Cysteinyl-tRNA synthetase) | Cysteinyl t-RNA synthetase SYCO ARATH (SYCO) is expressed and required in the central cell | [[19](#_ENREF_19)] |  |
| SSH-A36(Polygalacturonases, PGA) | PGA are believed to be responsible for various biological processes, such as seed germination, organ abscission, pod and anther dehiscence, pollen grain maturation, fruit softening and decay, xylem cell formation, and pollen tube growth | [[20](#_ENREF_20)] |  |
| SSH-A39(Ubiquitin 11-like, UL) | UL regulated plant growth and development by 26 s proteasome pathway | [[18](#_ENREF_18)] |  |
| SSH-A41(O-methyltransferase, Putative, COMT) | COMT could regulate lignin biosynthesis | [[21](#_ENREF_21)] |  |
| SSH-A59(Galactono-1,4-lactone dehydrogenase, GLDH) | GDP-d-mannose pyrophosphorylase could play an important role in the regulation of ascorbate accumulation during radish fleshy taproot development | [[22](#_ENREF_22)] |  |
| SSH-B3(Heat-shock protein) | Hsp affect chloroplast development and meristematic tissue abnormalities were happed in Hsp mutants | [[23](#_ENREF_23),[24](#_ENREF_24)] |  |
| SSH-B5(Phosphoinositide 5-phosphatase, Putative, 5TPase) | Primary root protophloem differentiation | [[25](#_ENREF_25)] |  |
| SSH-B14(Cytochrome c oxidase subunit) | Flower development | [[26](#_ENREF_26)] |  |
| SSH-B19(Phenylalanine ammonia-lyase 3, PAL) | Overexpression of PAL in transgenic tobacco plant could increase lignin biosynthesis | [[27](#_ENREF_27)] |  |
| SSH-B33(Translation initiation factor-like protein, EIF) | *AtEIF3f* is required for pollen germination and embryogenesis | [[28](#_ENREF_28)] |  |
| SSH-B47(Hypothetical Chloroplast RF2) | Chloroplast ribosome release factor 1 (*AtcpRF1*) is essential for chloroplast development | [[29](#_ENREF_29)] |  |

**Reference**

1. Zhang Q-Q, Zhu J-H, Cai Y-B, Zhang Z-L (2009) Cloning and Sequence Analysis of HbbHLH1 Promoter from *Hevea brasiliensis*. Molecular Plant Breeding, 2009, Vol7, No3, 531-536 7: 531-536.

2. Liu Q, Kasuga M, Sakuma Y, Abe H, Miura S, et al. (1998) Two transcription factors, DREB1 and DREB2, with an EREBP/AP2 DNA binding domain separate two cellular signal transduction pathways in drought-and low-temperature-responsive gene expression, respectively, in Arabidopsis. The Plant Cell Online 10: 1391-1406.

3. Bae EK, Lee H, Lee JS, Noh EW (2011) Drought, salt and wounding stress induce the expression of the plasma membrane intrinsic protein 1 gene in poplar (*Populus alba*xP. tremula var. glandulosa). Gene 483: 43-48.

4. Velivelli SL, Lojan P, Cranenbrouck S, de Boulois HD, Suarez JP, et al. (2015) The induction of Ethylene response factor 3 (ERF3) in potato as a result of co-inoculation with Pseudomonas sp. R41805 and Rhizophagus irregularis MUCL 41833–a possible role in plant defense. Plant signaling & behavior 10.

5. Lopez A, Ramirez V, Garcia-Andrade J, Flors V, Vera P (2011) The RNA silencing enzyme RNA polymerase v is required for plant immunity. PLoS Genet 7: e1002434.

6. Moons A (2003) Osgstu3 and osgtu4, encoding tau class glutathione S-transferases, are heavy metal- and hypoxic stress-induced and differentially salt stress-responsive in rice roots11The nucleotide sequences reported in this paper have been submitted to the EMBL, GenBank and DDBJ nucleotide sequence databases under the accession numbers AY271619 and AY271620. FEBS Letters 553: 427-432.

7. Tan X, Meyers BC, Kozik A, West MA, Morgante M, et al. (2007) Global expression analysis of nucleotide binding site-leucine rich repeat-encoding and related genes in Arabidopsis. BMC Plant Biol 7: 56.

8. Nie H, Zhao C, Wu G, Wu Y, Chen Y, et al. (2012) SR1, a calmodulin-binding transcription factor, modulates plant defense and ethylene-induced senescence by directly regulating NDR1 and EIN3. Plant physiology 158: 1847-1859.

9. Hettenhausen C, Yang D-H, Baldwin IT, Wu J (2013) Calcium-dependent protein kinases, CDPK4 and CDPK5, affect early steps of jasmonic acid biosynthesis in *Nicotiana attenuata*. Plant signaling & behavior 8.

10. Ercetin ME, Gillaspy GE (2004) Molecular characterization of an Arabidopsis gene encoding a phospholipid-specific inositol polyphosphate 5-phosphatase. Plant physiology 135: 938-946.

11. Król P, Igielski R, Pollmann S, Kępczyńska E (2015) Priming of seeds with methyl jasmonate induced resistance to hemi-biotroph Fusarium oxysporum f. sp. lycopersici in tomato via 12-oxo-phytodienoic acid, salicylic acid, and flavonol accumulation. Journal of plant physiology 179: 122-132.

12. Yan Z, Li X, Chen J, Tam NF-Y (2015) Combined toxicity of cadmium and copper in *Avicennia marina* seedlings and the regulation of exogenous jasmonic acid. Ecotoxicology and environmental safety 113: 124-132.

13. Keren I, Bezawork-Geleta A, Kolton M, Maayan I, Belausov E, et al. (2009) AtnMat2, a nuclear-encoded maturase required for splicing of group-II introns in Arabidopsis mitochondria. Rna 15: 2299-2311.

14. Li X-J, Luo Y, Yi Y-F (2013) P115 promotes growth of gastric cancer through interaction with macrophage migration inhibitory factor. World journal of gastroenterology: WJG 19: 8619.

15. Ulvskov P, Paiva DS, Domozych D, Harholt J (2013) Classification, naming and evolutionary history of glycosyltransferases from sequenced green and red algal genomes. PloS one 8: e76511.

16. Trevisan S, Pizzeghello D, Ruperti B, Francioso O, Sassi A, et al. (2010) Humic substances induce lateral root formation and expression of the early auxin‐responsive IAA19 gene and DR5 synthetic element in Arabidopsis. Plant Biology 12: 604-614.

17. Granot D, Kelly G, Stein O, David-Schwartz R (2014) Substantial roles of hexokinase and fructokinase in the effects of sugars on plant physiology and development. J Exp Bot 65: 809-819.

18. Rolland F, Baena-Gonzalez E, Sheen J (2006) Sugar sensing and signaling in plants: conserved and novel mechanisms. Annu Rev Plant Biol 57: 675-709.

19. Kägi C, Baumann N, Nielsen N, Stierhof Y-D, Groß-Hardt R (2010) The gametic central cell of Arabidopsis determines the lifespan of adjacent accessory cells. Proceedings of the National Academy of Sciences 107: 22350-22355.

20. Kim J, Shiu S-H, Thoma S, Li W-H, Patterson SE (2006) Patterns of expansion and expression divergence in the plant polygalacturonase gene family. Genome biology 7: R87.

21. Tsai C-J, Popko JL, Mielke MR, Hu W-J, Podila GK, et al. (1998) Suppression of O-methyltransferase gene by homologous sense transgene in quaking aspen causes red-brown wood phenotypes. Plant Physiology 117: 101-112.

22. Xu Y, Zhu X, Chen Y, Gong Y, Liu L (2013) Expression profiling of genes involved in ascorbate biosynthesis and recycling during fleshy root development in radish. Plant Physiology and Biochemistry 70: 269-277.

23. Cao D, Froehlich JE, Zhang H, Cheng CL (2003) The chlorate‐resistant and photomorphogenesis‐defective mutant cr88 encodes a chloroplast‐targeted HSP90. The Plant Journal 33: 107-118.

24. Ishiguro S, Watanabe Y, Ito N, Nonaka H, Takeda N, et al. (2002) SHEPHERD is the Arabidopsis GRP94 responsible for the formation of functional CLAVATA proteins. The EMBO journal 21: 898-908.

25. Rodriguez-Villalon A, Gujas B, van Wijk R, Munnik T, Hardtke CS (2015) Primary root protophloem differentiation requires balanced phosphatidylinositol-4,5-biphosphate levels and systemically affects root branching. Development 142: 1437-1446.

26. Curi GC, Chan RL, Gonzalez DH (2005) The leader intron of *Arabidopsis thaliana* genes encoding cytochrome c oxidase subunit 5c promotes high-level expression by increasing transcript abundance and translation efficiency. Journal of experimental botany 56: 2563-2571.

27. Howles PA, Sewalt VJ, Paiva NL, Elkind Y, Bate NJ, et al. (1996) Overexpression of L-phenylalanine ammonia-lyase in transgenic tobacco plants reveals control points for flux into phenylpropanoid biosynthesis. Plant Physiology 112: 1617-1624.

28. Xia C, Wang YJ, Li WQ, Chen YR, Deng Y, et al. (2010) The Arabidopsis eukaryotic translation initiation factor 3, subunit F (AteIF3f), is required for pollen germination and embryogenesis. Plant J 63: 189-202.

29. Motohashi R, Yamazaki T, Myouga F, Ito T, Ito K, et al. (2007) Chloroplast ribosome release factor 1 (AtcpRF1) is essential for chloroplast development. Plant molecular biology 64: 481-497.
