# Supplementary material for: Analysis of Differentially Expressed Genes Associated with Coronatine-Induced Laticifer Differentiation in the Rubber Tree by Subtractive Hybridization Suppression
Source: PLoS One. 2015 Jul 6;10(7):e0132070. doi: 10.1371/journal.pone.0132070 (PMC4493031; doi:10.1371/journal.pone.0132070)
Supplement: S7 Table — The table included a total of 256 ESTs sequences that the vector and adaptor sequences were detected, of which 147 ESTs came from the forward SSH library, while 109 ESTs came from the reverse SSH library. (DOCX) [file pone.0132070.s010.docx]

**S7 Table. The sequences of all ESTs in the forward and reverse libraries**

>SSH-A1

ACATAGAAACTGAGGTTACAGAGAAATTTAAATTTAAGAGAGAACAATCATTAATGTCTTCACTTTGTGGCAACCAACCTATAATTGGATCGTTGGAACATGAACATGGTTGCCTAATAAAGCTGCCATCATCATTCACCTAATACCAACCAGCCAAGAGAGAGGGAAGCCAACCGGTGCATAATTGCCCGTATCTTAGACATGGAGTTAAATACCAGACGAAATTAAAACTTAATTCTTAAAATTCCCAGCTCCAATCTTCTGCACTTCAACTTTGAGAGACTTTAGATATCTGACTGCTTCATCAATAACCATTACTGTATTCATTTGATCACCACCAGGT

>SSH-A2

ACTTAACAAGGAAAGACACAAATTCTCATGTTAAAACATACTTCAAAAATTTTGCTCTGATACCACTAAAACATGTCACACCTTACTCCTCTGTAAGGCATAACATGATCCCGTAGAATACCTAATGAACTACCGAACATCACCTATCGATAACCCATTAAGTACCAGAATTTAGCTGGGAAGATGGAGTCAGATCTAAAGAGTCTGTCTGATGCTTACAACAGCCTTGAACAGGCCAACTTCCATCTAGAAAAGGAGGTGAGAGCTTTGAAGAATGGTGGAGCATCGACAGTTCCAGATATTGAAGCACTAAAGGCAGAAGCTAGGGAAGAAGCCCAGAAAGAGAGTGAAGCAGAACTGAATGATTTGCTAGTGTGCCTTGGGCAAGAACAGAGTAAGGTAGAAAAGCTGTGTGCAAGGCTGTCGGAGCTAGGAGGGGATGTAGATAAACTGCTTGAAGGTATCGGAGATGCCATGGGGCTGCCTGAAGATGATGAAGCAGAGGAAGACTGACGAAGCACTTATTCATGATGGTTTGAAGCCTCTTCTAATAGACTTTGGCTTCGTACTGGCCCGGGCGGCCGCTC

>SSH-A3

ACAAGGAAAAATCTGCAAGTAATTCAGCATCAGAAGTGATGATTTCTGTCAAAAACAGAATGAGTCTAAAACACTCTTATGCTTCAAAAATGAAGAAAAAAAAAAAAAAGGGAACCCATGTCTGGATTCTGTGTCATTTAAATAGAGAAGGTAAAAAACTCATAAGCCAGGTAAGGAAGCTGTGGAAAGTGCAGGTGGTGTTGGATTGAAGAATTCATCATCAGTCCAATTCGTCTCTCCCCAATGTCGTAGCATACTCTTCCA

>SSH-A4

ACATGTCCAGCAAGAAGACCATGGAGATAAATCCTGAAAATCCCATCATGGACGAATTAAGAAAGAGAGCTGATGCTGACAAGAATGACAAGTCAGTGAAGGATCTTGTGCTGCTGCTATTCGAGACTGCTCTTCTCACATCTGGTTTCAGCCTTGATGACCCAAATACCTTCGGCAACAGGATCCACAGGATGTTGAAGCTTGGACTGAGCATTGATGAGGATGCTGGTGAGGGAGATGCTGACATGCCTGCCCTGGAGGAAGCTGATGCAGATGCTGAGGGTTCCAAGATGGAGGAAGTTGATTAAGCTTTTAGACTTATCATTTCTGATTAGCTTTTCAATCGGTGGCTACTTTTTTTTCGAAAAAATCATATGGTGATGTAGT

>SSH-A5

ACCCAGTCCAACACCCCATTATTCCCTGCTCTCCATGGAGGAAATTTCAACACCAATGTGCTGGACAGAATTGGCTGGCTAGTTCCGGTTCTTGCATTTCTCTACAGCTCTTGGGGCTAGGCCAATGGCATCAGAGCTCTTCATGATCCTCAGCCTCTTGCACGTTTCAACAAACATCTCCCATGGAACATCACCAACAAGCATCCAGTCACCATCTTTATCCTCGTAGGTAAGAACATATTCTGAGCCATGAAGAAGATCCTTCAGCTTGGTCTCGCTTAGCACCTCTCTCCCCAGAGCTCCATGAGATCCATATTGACCTATAGTAAAGCAGCTGAACATCTTTTCAAGGGCAGAAGACAGTTCCTGATATGCAGGGTAGTTTCTCAAGTCTACTTTCCGTAAATAAGGGGCACCGTCCATGCTAACCTTGACAAACAAGGGACCAGACCCTGCTTTTCCATCCACTTCTTCAGTATTCTTTGATGTGGTAGCAAGAGAGTTCTTCCTAAATGATCTGATAGGTGGCCAACCCACAACCTGCGCCTTGGTAGCAGGTGCACTACTGTTGTTGTTTGCAGAAGCATTATGATTTGGTCTTGTATCATT

>SSH-A6

CACGCATGGACTGGAAGGAGACCCCTGAGGCTCACGTTTTCAAGGCTGATCTTCCAGGATTAAAGAAAGAGGAAGTGAAGGTGGAGATAGAAGAAGGCAGGGTTTTGCAGATAAGTGGAGAGAGAAGCAAAGAGAAGGAAGAGAAGAACGACAAGTGGCATAGAGTTGAGAGATCAAGTGGGAGATTTTTGAGGAGGTTCAGGCTGCCAGAGAATGCGAAAGTTGATCAGGTTAAGGCAAGCATGGAGAATGGAGTCTTGACAGTGACTGTGCCCAAAGAGGAGGCCAAGCAACCAGATGTCAAGGCCATTGAGATCTCCGGTTGAGTGAAATGCTACGCAAACTCGATCTTTGGTGTGTTATACAGTTTGCCGAATAAATAAGTAGATGTGTGTAATCTGCAATGTAATGGGGTTTTATGGGCTTGTTTGAATGGAGTTCAGCTTTTAATGT

>SSH-A7

ACATTGTGCCCATCTCTTTCTTGACTAGGCCAGAGTTTCAAAGCTTGCTTCAACAAGCAGAAGAGGAATTTGGGTTTGATCATGATATGGGTCTCACCATTCCTTGCGAAGAAGAAGTTTTTCTATCCCTAACATCCATGCTCAGATGAGGACATTGTTAATTTATTACGTTGTTCAGGTTTTATTAGGCTTATGATCTAGACTGTAGAGATGAAGCTGCTTCATTGCTTCTCTTGTTTAATTTTCTCTTTTATAATTATTTTCTTTCCTCGGGTTTTTTCCTTAACTCTGACCCATGAAAGAACAGAACCCATGAATACTATTTGGGTTTCAGAAATGTAAATCTCCAAATTTTTCCTGACAGTGGTGCGGTGTTTGTCAACAAGCAACTACCAATGTTGTATCCTGCAGGAAGAGAACTAATATATTATTAAGGACACATCCCTGTTCTTTTC

>SSH-A8

ACCAAGGTGCTGGTGCTGACATGGGTGGTGGCATGGATGATGATACTCCTCCAGCGGGTGGCAGCGGTGCAGGCCCCAAGATTGAGGAAGTTGACTAAGGAATCTATTTGATGTTGTAGCAGGGTGATGAAAAAAATCATTTGGCCGATCTATTGATCGGCATGATTTTAATGCTTTAGTTTCTATTTTTACTTAGTTTGCTAGACATTATCATCTTTTCTGTCATCCATTTTTATTTAAGATTTTATGCCATTAGCGGGACAAATTTTCTGATGCTACATTGGCGGCTTAATGCTGCATTATTTATAAAACAGTTGCGAGAATTTATTCC

>SSH-A9

ACCAGGGTGGTGCTGGTCCTGACATGGGTGGCGGCATGAATGAGGATGTCCCATCAGCTGGTGGCAGTGGTGCTGGCCCTAAAATTGAAGAAGTTGATTAAGCTGCAGTTTGAGCAAGAAGTCGCGTTGGATTTTCTGTTTGTAGTTTTGATGGGTGTTTGTTTTTTCCCTTTTCTGTGTGTGGTTTGAATGGGGTTTTCGGAGTGGAAGACGGTGCAGTATTTTGCCCATCCCTCTAGATATGAAGTTCCTCTCTCAGTCTTATGTTAGTGTGCAGTATTTTCTTTTTCTTACTGGCACTCTTTACATGAAGTGATATTTTAATTGAATCATCTAATGGTTGAGATT

>SSH-A10

ACAAATTCTCCATGTTTAAGCAGGAAATAAAAATACAGCTATCCATGGAGGCAGATTTACATGAACATAAACATTATTTATCCTAACAATGACACTCTACCCAAGACCTTGACAACCTTGGCTCTGCAACTGCTAAAAACTTCATTGCAGCAACCAAATCCCTAAAATTCTGAACACGAAAGAACT

>SSH-A11

ACCACCAAGTTGTGATCAGAGCCATTCCTTTCAAGAAGTGATCATATGATGCATATATGTGGCCGTTCGATTATGTTTTCCGATCAAGATCAAGATAAAGCTCAGTTTGTTTGCATTTTCTATGTTATATTACTTTGTATTTGT

>SSH-A12

GCCAAGCCTAAGGCTGCGCCTGCTAAGAGTAAGCCTGCTGCAAAGCCTAAGGCTGCTCCGGCTAGACCAAAGCCAAAGGAGAGACCGGCTAAGGCGGCTAGAACATCGGCGAGGACGAATCCAGGAAAGAAGCCAGCGGCTCCCAAGGCGGCATCGAAAAAGGCTCCTGTTGCAAAGAAGACTCCTGCCAAAAGCGTGAAGGCGAAGAGCGTAAAGTCGCCATCGAAGAGGGCTTCAGCTAGGAAGGGAAGGAAGTGATGGTGGCGGAGACATACCATATGTTGTAGTGTTTGTAGGGTAGTTTTGTAAATTCGGTGGCTTTGAGGTGAATTTGGATCCATGTATGCCAGTTGGACAAAAATGAAGACATCGGATCTTTTTCTATTTTATTTTATTTTAATTTCTTAATTTTATTTAATGGATTTCAAACAATATAAGTTGT

>SSH-A13

ACCAAAAGCTCCTTAGAACACTTTCCACAAAGGATTTAACATTTAAGAATGTTCAGATGTTCTTGATACTACATATCAGACACAACCATCCCTTATTTACAGAAGCTTACAGGCAATTAAGGGTTTTTTCAATAAACCAGATTAGGCAGGTCATCAAAGCTCCACAGGTCCATTGGATTTCCACCATCCTGAGTCGAGTCTCCATTAAGGAAGTTATCTAGTGAGGCCTCCCAACTTCCCTCAAGATATGGCATCTGAAAGTTGACCTGGTTGTCAAAAGCCAACAGCTCTTCAGATAGAGATTTTCCATTGTTTTCTTCTACCGGCACTACATTTCCAGAGTCAGGCTTCATCTTCTTTAAAGGGTTAGCATCCTCCACAAACAAAGATTCATCAATTTCTGGAGTAGCTG

>SSH-A14

ATCAAAATAATTGAACTCAATAAATTATGGAAAAACTTGCAGCAGCATGAACAGACTAGCAAATGTTTGAATTATTTAAGAAAATTAGATTCTATGCTGGCATCCAGCACTAGCATTATCACCATTCACTAATCCAAAGAATGGGCATAAGTCTGGGTTGTAGACATCTTGTGTAAACCCTATGCTACAAACTTCAGGATGGCATTCTGCACAACTTCTCTGGTTGGCAAAGCTGGAAT

>SSH-A15

ACATGAAATAAAAGGTAATAGAATAATAGCAATAATGAACTAGTTACATCTTTTCACAACTTTATTATACCTCTAAATTACTGAGACCCAAAATTATCAACACCAAATCATCTGCTCCCAGGTTCCATTCCCACAAATGTAGCATATTCGAGAACCGTGTAGCCTTCATGAAAAGTCCTCCTTACATTAGACTCATAGCTCTCTATTGAATTTTAAGGTCATTGTGAATACAAGAGGTGATAAACATGGCAATTCAGTTACATTGAAACTTTAACATGTTGCTCATCCTAAAAAGGTTAAAAAATTGACAATAAAAGAAATATAATATTTTAAGGAAAAAAATACACCTAGCCTGGGCTCAATTTCTTTAGATTCTAACCCAGAAGGTTATCTCACCCTTTTTTTCCCTAACTTCTGTTGTAACAATAAAATGGTGAATGAATATTTTTCCCTTTCCAAAGTGAACTTTCTCACTTCTCCTTCCTGTGTTGTTAATGTTACCCTCAACGCCACTAACAACAAAACCCCAAAAAAAAACATGAAATTA

>SSH-A16

ACTACGATCATTGGAGGAACAATACCTAAACCTGAAGATGCTCCAGAATCTTTTCGATTGCTCGTTCGAGAACTACGATCTTTGGCTCTGGAACTGAATCATTTCCTTGTATCTGAGAAGAACTTCCAGATTAATAGGAAGGAAGCTTAATCGAAATGAATCAGAATTTTTCTTCTATGATTGATCGGTATAAACATCAACAACTCCGAATTGGATCAGTTTCGCCTCAACAAATAAGTGCTTGGGCCAATAAAATCCTACCTAACGGAGAGATTGTTGGAGAGGTGACAAAACCCTATACTTTTCATTACAAAACCAATAAACCTGAAAAAGATGGATTATTTTGTGAAAGAATTTTTGGGCCTATAAAAAGTGGAATTTGTGCTTGTGGAAATTATCGAGTAATCAGGAATGAAAAAGAAGACCAAAAATTTTGTGAACAATGCGGAGTCGAATTTATGGATTCTCGGATACGAAGATATCAAATGGGCTACATCAAACTAGCATGCCCAGTAACTCATGTGTGGTATTTGAAACGTCTTCCTAGTTATATCGCAAATCTTTTAGATAAACCTCTTAAAGA

>SSH-A17

ACACAACTCCACTTCAGCACAGCCTTCCATCCTCAAACGGATGGGCAATCAGAAAGAGTAATCCAGGTCCTTGAGGATATGCTGAGGAGTTGTGTCATTGAGTTTGAGGGAAGTTGGGATAAATACCTCCCACTGGCAGAATTTGCATACAACAATAGCTACCAAGCTAGTATCCAAATGGCCCCGTATGAAGCAACTGTATGGGAGAAAATGTAGAACTCCAGTGTGCTGGACTGAATTGGGCGAAGACAAACTGGTGGGGCCAGACCTGGTGAAACAGACTAAGGAGAAAGTGAAACTAATCAAAGCTAATCTGAAGGTTGCCTCAGACAGACAGAAATCTTATGCCGATCTAAAGAGAAAAGAAATAGAATATGCGGTTGCCGACAAAGTGTTCCTCAAGGTGTCACCGTGAAAGAAGGTATTGAGGTTTGGAAGAAAAGGTAAGTTAAGCCCTAGGTTCATTGGCCCATATGAAGTCATTGAACGTGTGGGTCCAGTGGCCTATAGGCTAGCTTTACCACCAGAGCTGGACAAGATCCACAATGTGTTCCACGTGTCCATGCTCAGAAGATACCGCTCAGATCCTTCACATGTCATCTCCAGGGAAGAAATTGAAATACAGCTGGATTTGACATATGAAGAAGAACCTCTACGGATCCTGGCTCGGGAAGTAAAAGAATTGAGGAACAAGCAGATTCCACAGGTGAAAGTGCTTTGGAGACACCACGACACCGAGGAGGCA

>SSH-A18

ACCTTTAACATTTTCTTTGTCATATATAAATATGAAGAGGAAGGGAAAAGAGTGTGAAAAGACCCGATACCTTGGGAATAATTGGAAGGAGACGCTGAAACCCTGCATGTTGCAGACAACCTCATAGATTGATTAGAGATATCTACATTGTTCATTGGCACAGGGTATGGCCGTGGCCTGTTAGATGAATTTGTCGCAGACATGGATCCACTGGGAGGATTTTGAGATGGTTTGCCTTGCCCAGAAATCGCAAATTTCAAAGT

>SSH-A19

ACGGATGTTTTATTTATCGCCAAATAGAGAGGAATACTATATGGTAGCGGCAGGAAATTATTTGGCGCTGAATCGAGGTGTTCAGGAAAAACAGGTTGCTCCGGCTCGATATCGTCAAGAATTCCTGACTATTGCATGGGAACAGGTGCATCTTCGAAGTATTTTTCCCTTCCAATATTTTTCTATTGGAGCTTCCCTCATTCCTTTTATCGAGCATAATGATGCGAATCGGGCTTTAATGAGTTCTAATATGCAACGTCAAGCAGTTCCACTTTCTCGGTCCGAAAAATGCATTGTTGGAACTGGATTGGAACGCCAAGTGGCTCTAGATTCAGGGGTTCCTGCTATAGCCGAACACGAGGGAAAGATAATTTATACTGATATTGACAAGATCATTTTATCGGGCAATGGGGATACTCTACGCATTCCATTAGTTATGTATCAACGTTCCAACAAAAATACTTGTATGCATCAAAAAACCCAGCTTCGGCGGGGTAAATGCA

>SSH-A20

ACCTATAGGGAACAAATGGGTTTTCAAGAAGAAAATTGGTTCTGATGGAAAGGTAGAGACCTATAAGGCAAGGCTAGTAGCGAAAGGGTTTCGCCAAAGGCAAGGAATAGACTATGAGGAGACTTTCTCACCTGTTGCCATGCTTAAATCAATTAGGATTTTATTAGCAATAGCTGCATACTATGATTATGAGATTTGGCATATGGATGTCAAAACAGCTTTTCTCAATGGATACATTGAAGAAAACATTTTCATGGAACAACCTAAGGGATTTGAATCCCAAGATGGTTCCAAGGTATGCAAGCTAAAGCGATCCATTTATGGGTTGAAACAAGCTTCGAGGAGTTGGAACATCCGTTTTGATGAAGCCATTAAATCTTTTGGTTTTATCAAAAATGAGGATGAGCCATGTGTATATAAGAAGGTTAGTGACAGTGCTATCACTTTCCTTGTCTTATATGTGGATGACATACTGTTGATGGGTAATGATACAGGTATGTTGACGACTATAAAGATATGATTGTCAAATACATTCTCCATGAAAGACTTAGGGGAGGCAACCTATATTCTTGGGATTCGCATCTATAGAGATAGAGAGAAAAGAATAATTGGTTTATCCCAAAGTCTATACTTGGAAAAGGTGTTAAAGAGGTTTAACATGCTTGATTCCAAGAGAGGATTGTTACCAGTGAGACATGGTATCCACCTTTCTAAAGAGATGTCTCCAAAGACACCTGAAGAAAGAGATAAGATGGCCAGGATTCCACATGCTTCGGCTATTGGAAGTTTAATGTATGCAATGTCGAACCCCCTGCCCGGGGGCGGGCCGCTTCGAA

>SSH-A21

ACTATACCTTCAGGTGGGTCAACAAGATCCCAAACTTGATTCTTATACATGGAATCAATCTCAGATTTCATAGCATCAATCCATTTTGAAGAGTCTATAATTGATATAGCTTCTTCATAGGTGAGTGGATCATCTCTATGATCTACTTCTTTATGAGTAGACAACTCTTGTTCTTCTTCAAGAAGGAAACCATATCTCACTAGTGGCTGAGATACCCTAGTTGTTCTACGAGGAGCTACTGTAGATGTTTCATCAATGGGTATAGGTTAACTAGATGGATCTACATCCATCTGATCTGTTGATTGGTCAGAATTCTCCAATTCCAACTCTATTTGCCTTCCTTTGCCTCTTTCCTGAACAAACTGTTGTTTAAGAAATGTGGCATCTCTACTTATCACAACCTTTTGTGAAGTAGGCAAATAAAAATAATATCCAAAACTATCTTTTGGGTATCCAAAAAATCGACCTTTTTCTGATCTGGTTTCCAATTTATTAGTGTTCAGCTTTTTGGTATAAGCTGGACAACCCCAAATCTTAATATGCTTAAGACTTGGTTTTCTTCCATGCCATATCTCATTGGGTGTAGAAGAAACTGATTTTTGATGGAATTCTATTCAGAATATACAAAGCTGATTCGATTGCAAATCCCCAAAAGGAGATTGGCATATCAGTATAGCTCATCATACTACGT

>SSH-A22

GTTGGTTGCATCTTATTGGAATTATGCTCCGGTGAGGCATTATTCCAAACCCATGAGAATTTGGAGCACCTAGCGATGATGGAAAGGGTCCTTGGGCCACTGCCTCAGCATATGCTGAAGAGAGTCAATCGACATGGAGAGAAGTATATCAGAAGGGGTAGACTGGACTGGCCTGAAGGTGCGACTTCAAGGGAAAGTATTAAAGCTGTTCTGAAGTTGCATCGACTTCAGAATCTAGTAATGCAGCATGTTGATCATTCGGCTGGAGATCTGATACATCTCTTGCAAGGATTGCTTAGATATGATCCCTCTGATAGGCTAACAGCTCGTGAAGCCTTGAGGCATCCCTTTTTTACAAGGGATCATCTAAGGGGGTGACTGCCAGATAGGCGACAACTAACGCTGACGCAGGCTGCTGTGAATGATGAGACGGCAGGGTTTACTAGCCAGGGAAAATGATACTGTTGTGCCCAACCATATTAAAGGCAGCGGATGTAAATAATGTAATTCCCATTGCCATCGAAAAGTTTGGCCTTTGCCTCTGAAATGGGGGCCGCCCTGTCATCTTGGTAATTTGCACCCAGTGCGCTGTCAAACCAATACTAACCCCAACTATACGAGTGGAACATGAACACAGAGAATCCACTTAAAAGGAAATGTATAAATTTTAACAGAAAAGCATCCATTATATCTAAATGAGGAAATGAAATACACCATGCATCATGCAAAAATCTCAAAATACCTCTTCAAAACTGACAGT

>SSH-A23

ACAAGATACATACTAAATGATATGCTCTAGGGCATACTACTAACAATCTCCCACTAGCACTAGAGCCATTCATTACAATATCTTAGACCCATCTTCTCAAGATGTCGGTTTGGCTGAGTCTGTGATATAGGCTTAGTGAATGGATCAGTTGGATTTTCAGCTGATGCTATTTTCTGTAAGGCTACATCGCCTTGTCCAACTTTTTCCCTGATAATGTGGTAGCGCCTTTTTATGTGTTTAGATTTCTGGTGAGACCGAGGTTCCTTAGCCTGTTTGACTGCTCCATTATTGTCACAGTGAAGATGAACTGCTGACTCAATGGAAGGAACTAATACAAGTTCTGTCACAAACTTCTTTATCCAAACAGTTTCCTTTGCAGCATCTGATGCAGTAATATACTCAGCCTCGGTAGTGGAATCTGCAGTCGTGCTCTGTTTGGAACTCTTATAACTGACTGCACCTCCATTACAAATGAACACATACCCAGAGGTAGACTTTCTATCATCGATATCTGATTGGAAATCGAAATCTGTATAACCATCCAATTACAAGTCTCCACCTCTATAAATCAAGAATAAATCCTTAGTTCTTCTCAAGT

>SSH-A24

TTTGCAGCAGCTAAAAGAGAAGGCGTTGAAGAGGGCTGAACTAACAGAGCAGGATGTGCTGGGGCTTATTGAGGAAAGAGCAATGGCGAGGAAAAACAAGGATTTTTCAAGAAGTGATCAGATCAGGGCTGATTTGACTGCAAAAGGCATTGCACTTATGGATGTTGGGAAGGAAACAGTTTGGAGACCATGTGTCCCTGTTGAACCAGAACAAGATGCGCCATCTGTTGTAGTAGAACAAAACCCACCACCATCTGTATCAAGTTGATGAATAGCTTTGTTCTGGCCCCAGCCCTGTTATAGAAGAGCAAAACCCCAGTTGCAAAAATGATAACCATAACTGTTTCTACGCTAGTAGAATGAAAAATCAAGAAAATTAGT

>SSH-A25

ACATCTGAAGGAGTCCAATCTCCAAGGGAGTTAAGTTTGGGTTTGGATTCAGATTCAGAGTCAAATTTCGAAAACGAAACCATGGCTGCTAGAACTTTGAAAGAGTTGACTACTCCTAATCTAAACCAACAGCCTTTGTGTATTCAATACCCTGCTTTAAATATTGCTTTTGAGTTAAAATCTGGACTAATCCATTTGTTGCCTAAGTTTCATGGTTTTGCAGGTGAGGATCCACATAAGCATTTGAAGGAATTTCATGTTGTGTTTTCCAGCATGAAACCTCAAGGAGTTTCAGAGGATCAAATCAAGCTTCGAGCTTTCCCCTTCTCACTGGAAGGCACAACTAAGGATTGGTTGTATTACCTTTCTTCTGGATCTGTCAACTCATGGAATGGGATGAAACAGATATTTTTGGAGAAGTATTTTCCTACTTCCCATGCTGCCAACATAAGAAAAGAAATTTATGGCACCCGGCAATATAATGGAGAGAGCTTGTATAAATATTGGGAACGATTTAAGAAACTGTGTGCAAGCTGTCCCCATCATCAAATAAGTGAGCAGCTATTGATTCAGTATTTCTATGAGGGACTTCTACCAATGGACCGCAGTATGATAGATGCTACTAGTGGAGGAGCTTTGGTTGACAAGACACCAGAAGAGGCAAGGAGGCTGATTGCTAACATGGCAGCAAATTCTCAGCAGTTTGGAATGAAAATAGATCACACACCTATGAAGGTTAATGAGGTAAGT

>SSH-A26

ACATACGACTTTCATCGAATACGGCTTTCCACAGAATTATATATGTATCTATGAGATAGAGTATGGAATTCTGTTTACTCACTTTAAATTGAGTATCCGTTTCCCTCCTTTTCCTGCTAGGATTGGAAATCCTGTATTTTACATATCCATACGATTGAGTCCTTGGGTTTCCGAAATAGTGTAAAAAGAAGTGCTTCGAATCATTGCTATTTGACTCGGACTTGTTCTAAAAAAGTCGAGGCATTTCGAATTGTTTGTTGACACCGACAAAGTCAAGGAAAACCTCTGAAATTATTCCAATATTGGACCTTGGACATATAATAGTTCCGAATCGAATCTCTTTAGAAAGAAGATCTTTTGTCTCACGGTAGCCTGCTCCAGTCCCCTTACGAAACTTTCGTTATTGGGTTAGCTATACACTTTACATGTTTCTAGCGATTCACATGGCATCATCAAATGATACAAGTCTTGGATAAGAATCTACAACGCACTAGAACGCCCTTGTTGACGATCCTTTACTCCGACAGCATCTAGGGTTCCTCGAACAATGTGATATCTCACACCGGGTAAATCCTTAACCCTTCCCCCTCTTACTAAGACTACAGAATGTTCTTGTAAATTATGGCCAATACCAGGTATATAAGCAGTGATTTCAAAACCAGAGGTTAATCGT

>SSH-A27

CAAAAAAGTTGTCAAGGCCCCAGTTTTGGCAAAGAAGAAACCTGAAAAGGTTGTGAATCCTTTGTTTGAGAAGCGAACAAAGCAGTTCGGAATTGGGGGTGCACTGCCACCAAAGAAAGACTTGACTAGGTTTGTCAAGTGGCCTCATGTTGTTCGTATTCAGAGACAAAGGAGGATTCTGAAGCAACGTTTGAAGGTTCCTCCTGCTGTTAACCAGTTCACAAAAACACTAGACAAGAACCTTGCTACACAACTCTTCAAATTGCTTCTCAAATATAGACCTGAGGACAAGGCAGCTAAGAAGGAAAGGCTTCTTAAAAGGGCACAAGCTGAAGCGGAGGGTAAAACTGTTGAATCAAAAAAACCCATTGTTGTTAAATATGGTCTTAACCACGTTACTTACCTCATTGAGCAGAACAAAGCCCAATTAGTGGTTATTGCACATGATGTTGATCCAATTGAGCTTGTTGTTTGGTTGCCTGCTTTGTGCCGGAAAATGGAGGTCCCTTATGCAATTGTGAAGGGCAAATCGCGGCTTGGAGCGATTGTTCACAAGAAAACAGCTGCTGCTTTGTGCCTTACATCTGTTAAGAATGAAGATAAATTGGAATTTAGCAAGATTTTGGAGGCAGTCAAGGCCAATTTCAATGATAAGTTTGATGAGCACCGCAAGAGGTGGGGTGGTGGCATCATGGGCTCCAAATCTCAGGCTAAGACCAAAGCTAAAGAGAAGCTCTTAGCTAAGGAGGCTGCACAGAGAATGTCTTGAGGTTCTAGGTGGTAGTGAAGAGAGTTATAATTATAAAGGAACTTGAGCTGATGAATTTGCATGTTGATTTTGATGGCAATGT

>SSH-A28

ACAAAACAGGTTGACGACCTTACAAAAGCCCTAAAAGACCCAATACCTCGGCCCGCCATCAAATACCTATTCAATACCTATGAGAAGTCCTATACATAAAATAAAACAGTATATCCTATTAGGTAATCTTCTAAGAACGGGAATGCTAGACCCTACCAAGTAAAGCAACTCCAACTGTCACTGACAACAGATCGGGTTCCCTAGCAATTAGGGTGCCAAGCTCTCTGAGCCCTCCATTTATTAGAACTGGGGACGCAAAGAGAAATTTTTTATACCAGGGTTTCATCAACGCGGGTATGGTAAGTTTGCTTCCTTACCTTTAGCTTAAGTTTCACAGAGTCAATCTCGTCTCTTCTCTAGAAAGAAAGCTGGCGGAATCAGATTAAGGGGACAATGAGTTAAAATTTATTCTTTGAGAAATCAATTGCAAAATGCTTTTCTATTTCTAGAATTCCCAATATTTGTTTTACATCTTCTATACGAAAATGTTCCATTTGAATAAGGTCTTCCTGGCTCTTATTCAAAAGGTCCGATAATGTATGTATATGAGAATAGGTTGCAGGCCTTTAAGCAGAAGAAGAATAGATGTTTTCTAGGGCGGCAAGGGCTCAAAGCTAGTCTCTTTCCATGGTGTGTCGGCAAAAGCTACGTTTCTTAATAGAGTTCGCTTTCTTTATCTCTCCGGTAGCAAGTCAAATAATGTAGGAATCGTTCTGTAGCAAGAGTCTTTATCCGGGGAAGAGCTTTACAGGCTGT

>SSH-A29

ACTTAAATATTATTTAATGGATGAAAACGGGAGAATTGTTAATCCCGATCCATGCAGTAACAGCGTTTTGAATCCATTCAATTTGAATTGGTATTTTCTCCATCATAATTATTGTGAAGAAAGATTCACAATAATTAGCCTGGGACAGTTTATTTGTGAAAATTTATGTATGGCCAAAAAGGGACCACATCTAAAATCGGGTCAAGTTATAATTGTTCACATTGACTCTGTAGTAATAAGATCCGCTAAGCCTTATTTGGCCACTCCAGGAGCAACCGTTCATGGCCATTATGGAGAAATCCTTTACGAAGGAAATACATTAGTTACATTTATATATGAAAAATCGAGATCTGGTGATATAACGCAGGGTCTTCCAAAAGTGGAACAAGTGTTAGAAGTGCGTTCAATTGATTCAATATCAATAAACCTAGAAAAGAGAGTGGAGGGTTGGAACGAGTGTATAACAAGAATTCTGGGAATTCCTTGGGGATTCTTGATTGGT

>SSH-A30

TATTTGCTTATGGAGTTGAGTATTTGACGTTAAAAAGAGAAGATTTAAATTTCAGTGAAATATAAGGTCTTACTGATGGATTTCAATAATCCGAATTCACAGTTTTTCAACAAACTCAAACAATAGATTTGGGCATTTAGATCCAAATCTTAAATTCCTATAATCCAAGTGTACACTACACAGTGAGGAAAAACAGAAAGACCCCACTCCCATATATCAGTTCATCTTTCCTTTTATGTATGCATTATCTATTCATTGTAAGTAGTATGTATTGATGATGATGGTGATCTTGCTGAGATCTTGTCCACAGATCTTTTTCTTTGTTGTATGGTTTTTGGCTATACAGCTAGAGGATTTCTTTTTCTTCCTTGTATAATGCTTTTCCAATATTTCCTAGCAAAGTCAATCTCCCCATTGCTGGCCATGCTCACCTCTGCCAACTACATGCCAATGGTTCCATGCCCGGAAGAAATCCTGGGCATTTGGAGAAAACACGAATCAAGTGAAGTCATGCTGAGAGTGGAATTGGTGGGCATTATGGGGTTGTTTGAAGATGTTTATGTCATGTTAAAATTTATCAAGTATTTGAATGAATCTGTTATAGCTACAAATGTTGCGTAGAGAATCTCCAAATTGCTAGAACATATGTATGTACTGGAAAAATCAATGAATTAATTGGTTGTTGTTTGAGTGAAAAAAAAAA

>SSH-A31

ACTTTGTCTCTTCTATTCTTTTGATAAGGAAAAGCTTAGCAAATGAATACAGGTATGATGATGGTTTGGCCCAAAGGCTATATCATTTTTTTTTTTTTTTTTTGCCTTTTTGTATAATAACTTCATTTTTTACTTTCTAGTGAACACCTGAAATTTGTCGGCTGTGTTCAATTGGGCTAGCTAACATACATCAAGAAGATGCTATTTGAATACTATTGCCAGAGGAACCAGAAATTACTCCAAAACATATGTGCAATCAGGAAAAAAAAAGGGCTTGTGTTGGAAAGCTTATCAGCCAAATTGCAGTAGCATTGCAATGTTCAAGTCCCAAGAACAAAAAAATGTTGATTCAAAATTTCTTTTTAATAGTTTCCAGCAGTGAAAAAGACCCCCAATCCCTGTTTTTTGGTTGATAAAAGCAAACTAAGTAAATGTGACGCCAAAATCACAAGAGTAGAGATGAGATTTCCTTCCCTTGAATGCATATATATAAGTAGGATCAGGTGGCTGCATATATATGAGTAGGATCAGGTGGCTGCTAATTAATCATTCGATGCATGATTATAAATGACAAGGACCTTATTCCCACATAATCATACTGCATGTGAAGTTGCAAACTGTTCTTGATTTTATCTAATGGGGTATCTCTTGGCAGGATGATCATAACAGATGTATTGGGAGGAGATATTCAGTTTGATTTCTATCACCGGTGGTTTGATGCCATCTTCGTAGCAAGTGCTTTCCTTTCTCTGCTTTTGCTTTCTGCACATTATACATCACGACAATCTGACAAACACCCAGTTGATTGATATATTCATCGTGCAAGGATCAGTCAATGAAATATCTTAGTTCTTGATCAATTGAAGCAGCTGTAAAAAATTTCCCAAATTTGAATCATCACATGACCAGTTCTTAAGCCTTTAATCAATATGACAGT

>SSH-A32

ACGTCTCGGCGACCCTTGAAGTAAACAACCATCTTATCCCTGGGAGGCAAATTGTCCTTGATCAAAGGATGGTTGATAAATCTGTGCATCCATGAAGAAATTGCTGGAAATTGCTGGCTCTCGAGGATCTTCATAGATCCAACTTCTTCCCATACAGGCAACCAGTAAGAGATCCATCCTACAGCAATGTCCACATATCCAATTCTTTCTCCTCCAAAAAATTGCTTCCCTTTTAGTTTTAGCTCTTCCTCTGTCTTTTGCAGGGCTTCAATGGTCAATTTTATTGCTCTTTCTTTTTCATCTCCAAAGGAGCACAGGGCATTCCATGCAGCTTCCGTAATCTTCTCTTCTGCAAATTTAGCCCAGAAGCGGGCCTGGGCTCTTTCATGAGGATCTTGAGGCAGTAACGTATACTTGTCCTTCCATGTCTCGTCGATATACTCGAGGATAATGAAGGACTCAGCGATTATTTTCTTGTTGTGAATAAGT

>SSH-A33

ACAGCCATACGACCGGCTATGTTATATGGTAGTGAGTGTTGGGCATTGAAAGAGTCGTATGCGTCTAAGATAAGAGTCATAGAGATGAGAATGTTAAGGTGGATGAGTGGCCATACTAGACTAGATAAAGTCCGTAATGAAAGTATTAGATTGAGGTGGTTTGGTCATGTGAAGCGTAGAAATACGGAGACTCCAGTTAGATAAGTAGAGCACATTAGGTTAGAAGATAGAAAGAAAAAAAGGGGTAGATCTAAATTAACTTAGAGGAGAGTAGT

>SSH-A34

GAAGTCATAAAAAAGGCCATTGAAAAGCTTGGATTAAGGCACAGGGAGCATATAGCTGCATATGGGGAAGGCAATGAGCGTCGTTTGACCGGTCGACATGAAACAGCTGACATTAACAACTTCTTATGGGGTGTGGCAAATCGTGGAACCTCAGTTAGAGTTGGTAGGGAGACGGAGAAAGCTGGGAAAGGCTATTTTGAGGACAGAAGGCCTGCTTCCAATATGGATCCTTATGTAGTCACGTCCATGATTGCAGAGACTACTATTCTATGGAAACCATAAGGAAGAGAGAAGCTACATTCTTTTCTGGTTGATTTCCTGAAGGGGAAACTTGGACAATTTTCCTTTGCTCTCTTTTGTTCAACCTCATCTTCAGTGTAGTAGTTTTTCCTTGATTGTTATGGTTGTATGGGCAAGGATTTCCTTCCATTTTGCCTATAATAATAGGATGATAATAACATTGGAGACAGATTATAATAATTGTTTGTCCTTTTTTCGGTTAAATAAAGAAAATTAATATTCAAATTTCAA

>SSH-A35

TGCAGTTAGGGAAGGACTGCATAAGCATATTCTCTACACATCTCAAAAGCTTGACAAGGATATTGGCTTTGCAGTCAAATGTTGTGAGAATTATTCTTCAGAATCAACTTTTGGATGGGAATCTGAGATATCTTTTCCCAAGGTTCTTGGAGATGTGATGGAGTCTCTGGCAGGAGCTATTTTTGTTGATTCTGGATACAACAAAGATGTGGTCTTTAGGAGCATAAGGCCACTTTTGGAGCCTTTGGTTACTTTGGAGACACTTAGATTAAATCCTACGAAAGAGCTTAATGAATTATGTCAAATAAAACATTTTAAAAAGAAAAACCCCTTTGTTCCTCTCAACAATGGCATGTCTTCTGTCACTGTTGAGGTTAAAGCTAATGGGGTAAAATTTAAGCACACATCTACAGCTGCAAACAAGAAGACAGCTGAAAAATTAGCTTCTAAAGAGGTTTTGAGGGTCTTGAAGGAAAAATTTAACATCTAGAGAAGATCAGATTGGTGTGCAATTAAAAGCTAGGATCAACTTTCTGATGCTAGCAGAGTCGCTTATCAGCTTAAGCAGCAGCCATTGGTGTTGCTGTCAAGACTGGGGCTATAAGAGTTAAAATTCTGCCATAATGT

>SSH-A36

ACCATTTGTATGCTACCTTGACACTTATTATTATATACAAATTCTGTTAGTGGAAGGTATCTATCCCATCTCCCCTCGGATTCAATGACACAAGTTCTCAGTATATTCTCGAGGCCTTATCACATATTACAGGTTATTATTATCATTTCATTTCATTATTTACAGGAACTCAAGGAGTATTAGAATTTACCTGAATTACTCATTTCAATTGTCCATCTTTCTGAGGATGATAAGTTGTACTCTCAAGTTGTAGAGCTTGTCCTTAGAAAAGAAGCTCACATATAATGCATGCCAATAGAACACTTCTGAAACTTTACCGAGTCAATAGGTATATCCTCCGGCGGAAAATTGCATTCTGTAAGTTGCTCTGGCCTTTGATCAGGCAATAAATCACACGGTCGAGGAGTCACTCCACTTGTGATCCCCTCAATATCAGTGCATGTCCATATTATCTTCTTGTGCTTGGCTGCCATTCCAATTGTGACATTAGAAATAC

>SSH-A37

TGAAGGCAGACAGAGTGGCACACCTAAGAGCAAAGTTGTGATAGCAGACAGTGGCGAAATACCACTATAGTTTGCTCTATGACTTGGCAGATATAACTCCTTAAACTTCCCGTTTTGCCTCTGTATAAGACTTCTTAATATGTAGT

>SSH-A38

ACCAGAAATAACGGTATCTCCAATTATAGCCCCTCTGGGATGTAAAATATATCTCTTCTCACCATCCCCATAGTGTATGAGACAAATGTATGCATTTCGATTAGGGTCGTATTCTATGGTTACGATTCTACCATATATGTCTTTTTCATTCCGTCGAAAATCGATTTTACGGTATAGACGCTTATGACCTCCCCCTCTATGTCTTGCGGTAATGATTCCTCTGGCATTACGGCCTTTACCACAACGATGCTGTCCATAGATCAAATTCTTTCGTGTATTTCGTGTATTGGATTTCGCTTGACTGTCTACGGCTCCATTGCGTGTGCTCGGGGTAGAAGTTTTGTATAAATGTATCGCCATGCTATTAAGTATTTTGATTTAAGTTCTTTTCTTTCTAAGAGGTGGAATAGAATA

>SSH-A39

ACTTACAACCATTCCAAAATAACAAGTTTCGGAGGGTTTAAAACTTATTTAAAGCCATTAAGGCATAACTAGTAGATATTAATAACAAAGTAGGCACAACCATGCAACTCAGAAGCCACCACGAAGGCGAAGGACAAGGTGCAAAGTAGACTCCTTCTGGATATTGTAATCAGCAAGGGTCCTCCCATCCTCAAGCTGCTTTCCAGCAAAAATCAATCTCTGCTGATCTGGTGGGATTCCTTCCTTGTCCTGGATTTTAGCCTTCACATTGTCAATAGTGTCCGAACTCTCTACCTCCAAAGTAATGGTCTTCCCAGTAAGGGTCTTCACAAAGATCTGCATGCCTCCACGAAGACGAAGAACCAAATGAAGGGTGGACTCCTTCTGGATGTTGTAGTCTGCAAGGGTGCGGCCATCCTCAAGCTGCTTTCCA

>SSH-A40

GCCATTCTTGCTTGGCCAATTCAGTTTGGTAGATATAGCCTATATTCCCTTTGTTGAAAGATTCCACGTCTTCTTATTAGAGGTATTCAAATATGATATCATCGCAGGCAGGCCTAAACTTGAAGCTTGGATTGAGGAGATAAACAAGATTGAGGCTTACAAGCAGACGAAAATAGATCCTAAAGAGAATGTTGAAGCATTCAAGAAGCGCTTTCTGGCTCGGTAATGTGCTAGATAGTGGTGGTTTTACTTATAGACTGAATAAGGGACTTGATGTCTCCTACATAATTTATCTTGCTTGAATAAAACATTTCGTGTATTAGTTTTGTAAGGATCAAACTCGTGTTAGTAATGTATTGATGAACAGT

>SSH-A41

GTCTGATCACTCTACCATAACCATGAAGAAAATTCTTGAGACTTATAAAGGCTTTGAGGGTCTTTCTTCTATTGTGGATGTTGGTGGTGGTATTGGAGCTGTCCTTAGCATGATCGTCTCCAAATACCCTTCGATCAAAGGCATTAACTTCGATTTGCCCCATGTAATTGAGGATGCTCCATCCCTTCCTGGCGTAGAGCATGTTGGTGGAGACATGTTTGTTAGCGTTCCTAAAGGAGATGCCATTTTCATGAAGTGGATATGCCATGACTGGAGCGATGAACACTGCTTAAAATTCTTGAAGAATAGTTACAAAGCGCTGCCACCAAATGGGAAGGTGATCATAGCAGAATGCATTCTTCCGGTGGCCCCAGACAGCAGTCTTGCCACCAGGACAACTGTTCATATCGACTGCATCATGTTGGCTCATAACCCCGGTGGGAAAGAGAGAACTGAAAAGGAATTTGAGGCATTAGCCAAGGGAGCTGGATTTCAGGGTTTTCGAGTGGTATGTGAAGCCTTCAATACCCATGTGATGGAATTCTTGAAGAGCGCCTAAAAGCCAAGTTTTCTCAATATTATCTTCTGATTTTTAACTACTCAAGTTGTGAGATGCGAAGATTTTCAAAGGGGGCTTTTACAGTATATGTAATTCTCAATAAAGGAAACAAATAAGAACTATATATATGATTGT

>SSH-A42

TGCCCCATGTAATTGAGGATGCTCCATCCCTTCCTGGCGTAGAGCATGTTGGTGGAGACATGTTTGTTAGCGTTCCTAAAGGAGATGCCATTTTCATGAAGTGGATATGCCATGACTGGAGCGATGAACACTGCTTAAAATTCTTGAAGAATAGTTACAAAGCGCTGCCACCAAATGGGAAGGTGATCATAGCAGAATGCATTCTTCCGGTGGCCCCAGACAGCAGTCTTGCCACCAGGACAACTGTTCATATCGACTGCATCATGTTGGCTCATAACCCCGGTGGGAAAGAGAGAACTGAAAAGGAATTTGAGGCATTAGCCAAGGGAGCTGGATTTCAGGGTTTTCGAGTGGTATGTGAAGCCTTCAATACCCATGTGATGGAATTCTTGAAGAGCGCCTAAAAGCCAAGTTTTCTCAATATTATCTTCTGATTTTTAACTACTCAAGTTGTGAGATGCGAAGATTTTCAAAGGGGGCTTTTACAGTATATGTAATTCTCAATAAAGGAAACAAATAAGAACTATATATATGATTGTACCTGGGCCCGGGCGGCCGCTCGA

>SSH-A43

ACCTCCATTTACACACAAAGATAGAAAGAAACTTCAGGAGAGAATTATCAAAGAGAAAGTCAAACTTCCACCATATCTTAGCATTGAGGCTTACTCTTTGCTCAAAGGATTGCTGCAGAAGGAACCATCAAGAAGGCTAGGCAGTGGTCCTGGTGGAGGGGATGTGGTCAAAGGTCATAAATGGTTTCGGTCAATCAACTGGAAGAAGTTAGACGCAAGAGAATTGCAGCCAAAGTTTAAACCAGATGTGAGTGGAAAAGATTGT

>SSH-A44

CACCGGTGTTCCTCTCCCAGCAACCTCTATCATCTTCTAATCCAATCAGCCTTTGAGCCATGTCGAAGAAGAAGACAAGAGAACCAAAGGAAGAGAATGTCACCCTAGGTCCTGCTGTAAGAGAAGGGGAACATGTTTTTGGGGTTGCACATATCTTTGCTTCTTTTAATGATACATTCATTCATGTCACTGATTTGTCTGGGGGAGAAACCCTGGTTCGCATCACAGGTGGGATGAAGGTTAAAGCTGACAGGGATGAATCTTCACCACATGCTGCTATGCTTGCAGCGCAAGATGTTTCTCAGAGATGCAAGGAACTTGGCATTACTGCTCTTCATATCAAGCTCCGTGCAACTGGAGGCAACAAGACCAAGACACCTGGTCCTGGTGCCCAGTCTGCCCTAAGGGCACTTGCTCGCTCTGGAATGAAAATTGGTCGCATAGAGGACGTGACACCAATTCCAACGGACAGCACCCGTAGAAAGGGTGGTAGAAGGGGAAGAAGGCTGTAAAGTGTTTCCCATGGAGTTCAATTCCAGTTTGGTGGTGGTGCTTTTATTAATTGGTTATGTTCAGT

>SSH-A45

CAAAATTTTTTTAACAATTCATTATGCAGGTTGCTAACCTTGTTGGCTATGTTATTGGGCCATCGGGCATTAATTGGTTGATTTCACAATTTCTCACAAAAAAAGGATTGCCTGTATTGGGTAGCATGTTTATTACATTTTATGTTGGGACAAAGCTTATGTTCCACATTGATGATGCCAAGAAAAAGAAGCACTAAACTAAGTGGACTATGAAGGTTCCATATGCAACCACCAGTTGTTAGAAGTGACAAGGAAAACATGGCTACAAAAAGTAGTATAGATGACCATGT

>SSH-A46

ACATCAAATACCTATCTGTATATTTGCAACACAAAAAAAAACCCTAAAAATTCCAACGATTAAGGGTGCTTTATACTCTTTGAACTGCGGTAGATATCAACTACTCTTCACAGAAAGACAGGCGCCATGACTAGCGTGATTGTTGCTAACATTTTTATAAGCACATGAAGTGAAGGCCCAGCTGTGTCTTTGAACGGGTCTCCTACAGTATCTCCAGTAACTGCTGCTTTATGACAGTTACTCCCCTTGCCTCCAAGAGCCCCTGTCTCAATATACTTCTTTGCATTATCCCATGCACCACCAGCTGTATTTAGGAAAAGAGCCATAAGAATGCCAGAAACTGTTCCAAACATCAGCATGGAAGCCACAACTTTAGCCCCAAGTAGAGGATGCCCCGTATAGT

>SSH-A47

ACTTCCAGATACTGTATTTGAAGCAGTTGTTCGAATTCCTTATAATATGCAACTGAAACAAGTTCTTGCTAATGGTAAAAAGGGGGCTTTGAATGTGGGGGCTGTTCTTATTTTACCTGAGGGGTTTGAATTAGCCCCTCCCGATCGTATTTCGCCAGAGATGAAAGAAAAGATGGGAAATCTGTCTTTTCAGAGTTATCGCCCCACTAAAAAAAATATTCTTGTGATAGGTCCTGTTCCTGGTCAGAAATATAGTGAAATTACCTTTCCTATTCTTTCTCCGGACCCCGCCGCTAAGAAAGATGTTCACTTTTTAAAATATCCCATATATGTAGGCGGAAACAGGGGAAGGGGTCAGATTTATCCCGACGGGAGCAAGAGCAACAATACGGTTTATAATGCTATAGCAGCAGGTATAGTAAGCAAAATCATACGAAAAGAAAAAGGGGGGTATGCAAATGTTTATGGCCATTTCTTGTTTTCAAAATCAAGACCAAGATCGAGCTCAGTTTGTTAGCTTGTTTATCTGTTTCATTACTGTGT

>SSH-A48

ACCATTGACCTAGAGTCACTGGTTACTTGGATTAATTATTCATTATACAAATCATCAGCAATTTGGAAATCAATTCTATATTTGAATGCAGAGCCACAATGCCCATCGAGAAGAAAAGCCTTAAATGGGTCTCCTTAAAAATTAAAATCGTAACTTATTTTGTGGCGAAAGAGCATCATATGATATAGGTGGAGCAGCAACCGTTGCTTGTTGATCAAACTCGGCTGCTGCGTTGCCATAGTTCTTACTGCAAGGAACAATGGTGAGTGATTTCTCATTAGGACATTTCCTGGGCTTGGAGAAGAATTCTCTACACTCTTGTGCTGGTCTTTGATTTGGAAG

>SSH-A49

ACAAAATGTCACGGGTTCAAATCCTGTCATCCCTACCTATTACTTCGTCTCTGAACAATAACGAGGGATCAATTGAGATCAATTAAAATTGGACATACCTAATCTTTAATTTTTATTATCTTATACTTATATATGATAGTATAGGGATTCAAGATATATTGGAGTTAAAAAAAGAATCTTTTTACTTACAGTTTCCTTTTTTGTGATTGTGATAAGAAAGCGCTCTTAGTTCAGTTCGGTAGAACGTGGGTCTCCAAAACCCAATGTCGTAGGTTCAAATCCTACAGAGCGTGATTCTGTTCTTGTTTTGTTAGGTCAAATTTTATACGGAAAAAATGGAAGAGAAATCTGAATTTGACCTCCTGCGGATTAAAGAAAGGAGGAGGTCAAAAAAACAAGGTGTTAATTAATCAAAGGTCCAACTGATCACCACGTCTGTATTGTAAATATGCAGTTACAAATAATCCGGCCAAAGTAATAGGAATTAGACCTAAGACGATTCCAAATAGAAAAACTTCAATCATTTCAATTTATTTGAAAAACATGCATCGTATACTGCAGT

>SSH-A50

CTTTTGTCTACCATTCCTTTCTACAAGAAACATAATTTTGGGGGAGAGAAGTTGAATACAACCTTGTTGGAAGAATGGATGAAAGAAAGCAAGGCACCAGCTCTAGATCACACCTTCAATCTTTTCCATGAGATCAAAAATAAAGGGGTCAAAATCTTTCTTATCTCCTCAAGAAGTGAAACCCTAAGATCTGCTACTGTGGATAACCTTATCAATGTTGGGTATCATGGATGGTCTAGTCTCATATTAAGGGGTCTTGAAGATGAACTCACAAGAGTGCAAGAATACAAAGCTTCAGCAAGAAAAAGATTGATGGATGAAGGGT

>SSH-A51

CGAGTCGATGACTATTGGCATCACTGGCAGGATGTATTTGCTGGGGGTCTTATAGGGCTGACAGTTGCTTCATTTTGTTACTTGCAATTTTTCCCTCCCCCATATGATATAGATGGCTGGGGACCTCATGCATACTTTCAGATGTTGGCAGAATCTCGGAATGGTTCTGAGTCTTATAATAACATGAATTGCCTTAATGTGCGGCAATCAGAGCTTCAGAGTGTATATATTGATTCTCAACATCAGAATGTCCTTAATCGGGACACAAGCCCCATTCTGGAGGGAGCAGAGGGTGAGAGGAGACATTGACTTCAGGTTTTGACAAAAGAAGAAGGGATACCATATGTGACATATCCATACCATGTAAACTGAATAGAATGTGTTCCTTTACATCTGCAGATATCCACACTTTATATGTTAGCTTGGCTCATCGCTGAACCTACTCTTTTTAGTGTGATTCGTTGATTCAATTTAAGCGTTTGGCTGTTTC

>SSH-A52

GGTGTTGGCTGGTTCTTGTTTATAATCGGTTTCTTTCTTGGTACTATCCCCTGGTATGTTGGACTATTTGTTCTGCTATGCGCAAGGATAGATCCCCGAGAGAAACCAGGATACATTGCTTGCACAATTGCTGCTGTTCTTGCCACCGTCGCAATTATTCTTGGTGTAACTAAGGGATCAGAAGATTAGCAATCGTTTGGCCAAGAGTAGCAAAATGGATGTGAACACACATGCATCAGTTCCAAAGTAATTTAGCACTCTTGAGTATGCAATACTGCGATTGATTCTTAGTTGTGTTAGTGGAGTGTTCTGAACAATTCGTTGGGAGATGATTTCCATAAATGTTTAATGCAGTTATTGTTGT

>SSH-A53

ACATCAAGGTTAACGAGATGCCCCTGAAGCAACAGCAACCACTGACACATCCAAGGGCCGGACTACTTGAAGAGATCTGGATTGAGCCAGTCCTTGGTAATGTCATCAAGGTGATCATCAAAATCTACCACATCCTGCCACTTCTGGGATGAAATAAAATCCAACAACACAATATTTGCTGCTGGCTCCTTAAGTATCA

>SSH-A54

ACCAAATTCTCAGACTCTCCCCCTCAGACATGACTAACCATTTGCAACTTGCAACCAATGACCATTTTTCTAGCCGCCATTCAAACCATCTTTAATTGTCATCATCAGCCCATCAAAAATAGCATTTGACTAAACTAGGGAAACTAGCTTTAAAATCCCATTAAATGGGAGAAAAGATAAATGCAGAAGTAGAATGTGGGTGGATGAGGATTCTGCAAGAGAAGGCAACGTTATAACAACTCCCCTTTCCCATGTCTTCGTCATTATCCCTTTTAGAATATCTTGAAAAGCAGGCCACCATGTGTGGCAAAGAAGGGAGCAAAGACGAGAGATTCCACAGCCATAAGCTTGATGAGAATGTTGAGTGATGGGCCTGAAGTGTCCTTAAGTGGGTCACCGATGGTGTCACCAATCACAGCTGCTTTGTGTGGATCTGAACCTTTTGGGCCTAGTGTCCTTGCATGCTCTGAAGCACCAGCCTCAATATACTTCTTGGCATTATCCCATGCACCACCAGTGTTAGATGCAGAAATTG

>SSH-A55

ACTCTGCAGCCTCTGCCTTTGCCTTGGCAATTCTTTTCTTTTTGTCAGCAATTCTTGCTCGCTTCCTTTGCAAAGTCAATGGAGTAACCAGCCTCTGAATTTTGGGTGCCTTACTCACCTTCTTTCCAGATTTTGTTGTGAATGATCTTCGGTAAGTGTTCACATACTTCCGAACATCATCCTCCTTTGAGAGATTAAAGAGTTTGCGGATCTTGGATGCTCTCTTTGGTCCCCTCATTCTTGGTTTCTCAGTATCAGTCAGTCCAGGCAAATCATTCTCTCCCTTCTTCACGATTACCAAGTTCAAAACAGAGAGGTCAGGGCTCACAATGCATCCACGCACAGACTTCCTCCTACGTTCTCCATTGCGCCTTCCATATCCACGGAAACAAGGGGTTCCTCTGTGAAGCAAGAGACGAACACGGCCAGGAGTCAACACTCCCTGCTTCATGGGGAACCCCTGCTTGTCACAACCTCCCATGATTTTGAAAACATAGCCCTTAAATTCCTCCCCAAGTGCATCTCCGCTGACCTCTTGTGAGATCCTCTTGTCAAAAAACGCACGGAGCTTTTGGTCATCGTCTATTTCCAGCTTCTTTTGGCATCCCGTGGTGGGGTTGGCAATGTTGAACTTCATCTTGGTCCGGT

>SSH-A56

CTGAATGTTTTTGAAACGAAATAAAGTTGGGATGGATTATCTTAAGCTTAAGCTTGTTTTATCGGGGGGACCATAGTAGCACTAGGCAATACATTACAACCAGTAAACATAACATGCCCATCCACATGATGACTCCATCTTAGCCAGGAGGCCAGTTCATTTGACGTCCCCCAAGGAGGTGGATGTGAAGGTGATAAACAGATTGACATCCATTCGGCCCGTCATTAATCACAATCCTAAAACCATCTTCAAGTCCTTCCTGCTTAGCAATGAGCTTGGCAGTGT

>SSH-A57

ACCCAGAGGATGATCCATCTAGAAAATTGCTTGAAGAGGTGCAGAGCAGCTACTTTTTGGTCAGCTTAGTAGATAATGATTACATACATGGTGATATTTTTGCGGTTTTTGCAGATTTATGAGGCTAGCTGCTGCGGGCAGCTCCTCTATTTTTTAAGATCCCATTTAAAGCCACTCAAAATGGTGTGTTTATTATAAGTTTAATCATAGCTGTGTTTCATTTTGGTGTTTTCGAACTCTTGATGATATGTAATAACAATTCATTGGGATTTTGATACCTCCCTTTGGGTTATGGTGTAAAAGCCATTTCAAGTGGTAGCAAATTTACTGGCCTAAAGCATATGTATTATTACCATTTTTAGTCATAAAGCTGAAGTTATCGATTTTACCCTTTGAT

>SSH-A58

ACAGAGAAATCAAGATTCTTTTAAGGAGTAAACAGAACATCATAAACACGAAGAGCTTGTATGTGTAAACTGTATAAGAAAGAACAAAAAATTAATAAAATAACATCTTAGCATATGAAAAGAGGAGCACCATTCCACTCCTTGAGGCATTCTAACATGGGGTCAATCAGTTTCCCTGCACACATTGCTGAGAAAACCTTGTCAAAC

>SSH-A59

ACAGTCAAGAACATAGACTGAAGCATTTCCAGCAAACTGGCAAAAGCATGATAAAATATCAAGCATTCACAAATAGCATGAAAGAACAAAATGACTAACCAATCAAAAAAGGATGCTTCTTAAATGGCTTCTGATAATGGGAACAGCTTTTCCAGCTTATTATTAGAAAGGATTCTGTTAGGGTCCAATTCCTTGCGTGCCTTATTATATGCATCCACGGGAAAACGCCTTCTTAGCCTTGCTTGCAGAGCTGTAAGATCTTCAGTGTCCTTAGGAACCTCAATCTTGGCCCAATGTTCAAAAGCAGAATACTTGTCCCACAACAGTGTCTGACTTAAATGCCTGTAGTGGAAGAATTCTTCTGTGATTTCCTTTCTTTGGCGAGCATCCATTGTAGGAAGATACATGATTATACCAACCCATGAGAATATATCATCTTTGGATGAGCTTGAAGCAGGACTCATGGAGCTCTCGCTGCGAGATGTCCAACGCTGCTCTATAGGAGCAGGTGCAGGTATCTCTTCTTTCTCTATGAGTTGCTTCAGCTCTTCTATAAATTCAAGGTCTTTCATGCTTGGTTTTGAAAAGGT

>SSH-A60

ACAATCCAACCTGCCTAGTAATGTGGCTCCAGTCCCCAGGTGTCCCTCTGTCATGTAAAGCATCATAGAGTTGCTGGCGCAGCTTAATAATGCGATCAACCATTGCCTTCAACTCAACAGTCCATTCAGTGACCATTTCCCTATCCTTTAGGATGGTAGCCACAATGGCTGCCCCATGGATGGGTGGGCTTGAATGCATGGGTCTGATCACAAGTTTTAGCTGGCTATTAACCCGGCTTGCCACATCTGCTGTCTTGCACACAATGCTAAGTGCACCAACACGCTCCGCATAAATTCCCATTATTTTGGAATAAGATTGAGCTACAAGGCATTCACCACCATCTGCTACAAATATTCGAATAGATTGTGCATCCATATCCAAATTTCCACTCACAAAACCCTGATACGCACAATCGAAAAAAGGTAACAGTCCTTTTAACCTTATCAGCTGCCTGATCTGCTCCCATTGCTCAAAAGTTGGATCAACACCAGTAGGGTTGTGGCCACATGCTTGGAGAAGCACAATGGCTCCTGATGATGCGCAACTAAGGTCCTCCAACAGTCCTTGAAAGTCCAGCCCATGTGTTG

>SSH-A61

TTTTTTTCAACCAAAAACTTTTAAGATCTTAAACATATATATCCTTAACAAATCTAATAGGTCCAACTTAAAGAGTGAAATTTCAAAATATTCCTTCGAAAATTCCAGTAGTAACTTCATAAGAAAACATGAAACAGAATAAATTCAATTCTTAGGTTCTGGCAGCAGTTCCAACTCCTGCAGAACGAATTCCTTGACCAGGCTGTCCAGGCTGTCCAGGCTTAGGCAAATCATCCCTGCGCCTGAGGAAGCGCTGTGGTGGCTCACGTTTCCTGCGCTCAGAGCGCGAGCGAACCCTCACAACCCTGGAGTGAATGGCACATGAAACGCAGT

>SSH-A62

ACAAACACAATATTGTGACTTTGAATTTTATTTGTTGAAGAACTTAAATATTCTTATCAAATTATGATAGAAATTGGATACTGATCGCCTCCTACAACCAAGAAATGTGTTCCTTCTTCCATCACCTTCAGACCATATTCATTAGCTCTACTGAAGTGCTCACAAGGGCTCAGTTCAAATTCAATTTCAGCTTTTTCCCCCGCGCTTAGTATCGCACTTTTG

>SSH-A63

CAGAGGACGAAGCACAAATAATAGTTGCGGCAATGAAAATTCAACATTCCTTTCGCAATTATGAGACACGGAAAAAGATGGCAGCTGCTGCTCATATCCAGTATAGGTTTCGTAGTTGGAAAATGCAGAAGGAATTCCTTAACATACATGAACATGCTATCAAAATTCAAGCTGCTTTTCGGGGTTTCCAAGTGAGAAGGCAATGCCGCAAGATAGTCTGGTCAGTTGGAGTGCTTGAGAAAGCAATTCTTCGATGGCGTTTAAAGAGAAGAGGCTTTCGTGGGCTTCGTGTTGATCCTGCTGAAGTAGTTGCAGAAGATATGCAGGAAAGCAATGCGGAGGAAGACTTCTACCATGATAGCCGGAAACAAGCTGAGAAGCGTGTTGAGAAAGCGGTTGT

>SSH-A64

ACCGATTGAAAAATTTTTTTAGATATTTTCTTGGCATTCTAATGCCATAGAAACTTCAATGACCCTTCTCTGGAGTCCCAAAAATTATTTTAGGAATTTTTTCACAGGTCTAGGGCTCCTAGTTGCGAGAACCGCAACTTCCCACTAGGTTACCCATCGCTAGGGCACCGGCTCATTTAACTTGGTTGTATTTTATTTCTAAAATTTTTTTCCTAAATTTTTCTTATTAATATTTGAGTTAATTATGGTTCTTCACTTTAGTTTAAATATTTTTCCGGATGTTCTAGCTGTCCGGACGGACACTGGTCACCGGAACAGTAGAATGTACGGAGTTGCTACAGAGAGGGTGTTATAACTCTTCCCCTCTAATTTAAATTTCGTCCTTGAAATTTACCTGATGCAAATAGTTGAGGGAACTGTTGTCTCATCGTCTCTTCATTTTCCCATGTTGCCTCTTTGGTATTGTGGTGCCTCCAAAGCACTTTCACCAGTGGAATCTGCTTATTCCTCAACTCTTTCACTTCCCGAGCCAGGATCCTTATGGGTTCTTCTTCATATGTCAAATCCGGTTGT

>SSH-A65

ACACTCAGCTAATGAAAAAACAAATGAGTCTGAGGGAAGTTCCAACGCTCATATAGAGTCGAGTAGTCCTGCTCCTGCCAATTGGAGGTCACAGGGAAGTAATGGTGTTCCTGGAATATCAGCTGAACACAAAGCCGCTATTCGGATTCAAACTGCCTTTCGAGCTTATATGGCAAAAAAAACAATGCGCCGTCTGAAAGGAGCAGTGAGATTTAATGTATTGATTCATTGCAATGATACTCAAAAGCAAGCTTCAAGCGCATTGAGCTATATACACTCATGGAACAATATACAAGCTCAGATTAGAGCTCGCAGGCACCATATGGTCACGGAAGGCCGGATTAAGCAAAAGAAATTAGAAAATCAGCTAAAACTTGAGGCCAAGCTCCATGAGCTGGAGGTGGAATGGTGTGGAGGCTCTGACACCATGGAAGAAATCCTTTCCAGGATCCAACATAGAGAAGAAGCAGCAGTTAAGCGTGAGCGAGCAATGGCATATGCCTTCTCTCATCAGTGGAGAGCCAATCCTAGCCAGTATCTCGGCCAGGCATATTACAGCATTGGCAAGGAAAATTGGGGTTGGAGCTGGAAGGAGCGTTGGATCGCTGCCCGCCCATGGGAGATTCGGATTCATGCTCAGCCTACTAACCCAAAGAAAGTTCAGAGTGTGGAAGTGAGCAAATCAGAGATGAAAATAACACTCGCAAGCAAGCCTGCTTTGTCAAATGGGAAGGTGAGCACCAAAGCTAAAAAACTGTCCAACACAATTGTTGATAATCAAGCTGCACAAGAAGCCAATAGTTTTTTTTTTTCAACCAAGAATATAGAGTTTCAAAACTCAAG

>SSH-A66

ACCGCGTGCCTTCTATTTTGATTTAATTGTGGTTGATTGACTATAACTTGCTTTTCTATTTTGCGTTTTAGTGTCTTGTTTGCCAAGTTATTTAATCTTTGGATGAGTGTATTCTAAATTTATCAACAATTGGATATTTAGTTTATATAGTTGTTGTTTTGTTCTCTTTCCTATG

>SSH-A67

CAAAGCATGGCCTTTTGGAAGGACAGACTTGGCCACACTACCATTGATTTAGGAATAGCCCCAGAAAAGCTTGAATCTTACCAAAATGGAGACATAAAAAAAACTGATCCCATGGAGAGATTGCAGGCAGTCCGTGGACATCTTGTTTCTTTGCCTTTGGATTTCATGTGCAAGGAAGATTTAAGACCTGTATTTAATGAGAGCGAATATTATGCTTCTCAAGTTTTTTATTGATCACTGACTAGTTGAAGAGCAGTAGCCTAAGTGTTTTTCATCCTTAGGTAAATGCTGTTGTTGCTAAGGCCTATGCCTGAAATTGGGAGGGGGGCTCTATTTATGGCCTCCTTGAGAATGTAATATGAAAACTGAACACTGTTGT

>SSH-A68

ACTCTATTTTACTTTAATGTGAAAACGTAACAATGAATTTATTGTCTTAATAATATTGGCCTTTATAATATTTTTTGCGTGGATTCGATAAGTTTATAAAAAATAAAATAAAGGAAGACAAAACAAATAGAGTCCAATTTTTACGAATAGGTCTTTTGAGTAATGAACAAATCTCTATGCATTTGCTCATATAAAATGGAGTCAACTCCCCATTGCGTATTGGT

>SSH-A69

ACCAGATACGAGATAGATCTTCCAAAGAACAAGGCTTTTTTCGAATAAGCCAATTCATTTGGGACCCTGCGGATCCACTCTTTTTCCTATTCAAAGATCAGCCCTTTGTCTCTGTGTTTTCACATCGAGAATTCTTTGCAGATGAAGAGATGTCAAAGGGGCTTCTTACTTCCCAAACAGATCCTCCTACATCTATATATAAACGCTGGTTTATCAAGAATATGCAAGAAAAGCACTTCGAATTGTTGATTCATCGCCAGAGATGGCTTAGAACCAATAGTTCATTATCTAATGGATTTTTCCGTTCTAATACTCTATCCGAGAGTTATCAGTATTTATCAAATCTGTTCCTATCTAACGGAACGCTATTGGATCAAATGACAAAGGCATTGTTGAGAAAAAGATGGCTTTTCCCGGATGAAATGAAAATTGGATTCATGTAATAGGAGAAAGGTTTCCCATTCCTTAGCCTGAAAGATATGTGGCCATGAAATAGGGATTAAGCGGAACAGAATTGACTGGGTGGTAGAGTCGTGGAAACACCTGTTTCTTCCATATTTTGGACATGGAACAATATGTTACTGCTGAAACATGGAAGAATTGAAATCTTAGATCAAAACACTATGTATGGATGGTATGAACTGCCTAAACAAGAATTCTTGAACAGCGAGCAACCAGAACTATTCCTCACTACATCAAAAAATTTCCATTAATGAAAGATGTAAATCCATTGGAAAATCAAAAATACGTATGTCGGATGAAATGGTGGTTGTTGCTATCTGCTCCAATAACGAATCATTGGTTTAACTGAATAACTAAATGAATAACTAAATAAAACAGATAGACCCTTCTCTTCGTCTCAGGTCGATGGATCTTCTCAATTGGAGGATCCCCTATATGGATAATACACATTCCAGTTGACCGGGCCTAATTCTAATTGTTTTGTTCCGAAGCAAAGATATCCACGGGGCGGTTCGTCCTATTCAGATATTCACGACCAAGAAGTACATGCATATGAATTGGATCTATGTATGT

>SSH-A70

ACCGGCTACGAAGGAGGGATCTGTATGATATCACCTCTGGTTCCCTTATACCGTTCGTTGAGTCACTTTCCTTCGTTTTCTTCCCAATCTATGACGAAAAGGGTCTTCTTCGGAAGCTCACTTTTGATATGGGTTTTCTTGTTCTTAAGTGATAGAGGGTCTTTAGGCACTCTCGTAAGGTTTGGAGCCTTTGGCGATATTCGGATTCGGAGAAGGATCCCACTTTGTCTTTCTTTGACTTTCCCTCCACGCGTTGGCTGGTTATCTTTGGGAGTAGAAGACTTCCTCTCTTTTCTTTGCCATTTCAAACATTGCTTTCTTTATTAACTTTCACTTCTTTCAGAATGGTATGCTTTTGTCTTCTTTCGTTGT

>SSH-A71

ACACAAGCAATCGCCACAGGAACCGGTATATATGAGTAATCGTAATGCACGCAATCAAAGGAACCGTAAACTGGATCTCCTTCGCTTCGTCCCCTCCACCGAAACCGCTTTCAATCGGGAAAGGAAGCCACCCGAACAACTTTTTCTATTGAATCTCCGAAAAGCCCTCTCCTTAGCCGGCGCAGCGTTGATATTGTATAACCTTAGGTAGCAGTTGAGTCACTCTCGTCCCTGGGTGGGGTTTAGTAATAAGGGTTCGAGGGGCTTGTTTAAAGGTTTGACATTCTCTTTAGCAAAGAAGAAAGCACTACTTCATAGGAAGTTGCCAACGGTAAGTCCGACGTCTTCTTAAGGTAAGAGATGAATGAGGCCTCTCACACACACACGCGTAC

>SSH-A72

ACTTCGAACTTGGAACGAGCATGAAGAAATTAACGATACTTCTTTATCTTTTGAGTTGTTCTGCCGGATCGGTTGCTCAAGACCTTTGGTCTCTACCCGGACCCGATGAAAAAATGGGATCACTTATTATGGACTTGTTGAGAATGATTCTGATCTAGTTCATGGCCTATTAGAAGTAGAAGGCGCTCTGGTGGGATCCTCACGGACAGAAAAAGATTGCAGTCAGTTTGATAATGATCGAGTGACATTGCTTCTTCGGCCCGAACCAAGGAGTCCCTTAGATATGATGCAAAATGGATCTTGTTCTATCCTTGATCAGAGATTTCTCTATGAAAAATACGAATCGGAGTTTGAAGAAGGGGAAGGAGAAGAAGTCCTCGACCCGCAACAGATAGAGGAGGATTTATTC

>SSH-A73

ACCTTGTGTCAGAATTGAGAAATTCTATGGCTCGAATCTTTAGTATTCTCTTATTTATTACCTGTGTCTACTCTTTAGGCAGAATACCGTCACCCATTTTTACTAAGAAACTGAAAGAAACCTCAGAAACGGAAGAAAGGGAGGAAGAAACAGATGTAGAAATAGAAAAAACTTCCGAAACGAAGGGGACTAAACAGGAACAAGAGGGATCCACCGAAGAAGATCCTTCTTCTTCCCTTTTTTCGGAAGAAAAGGAGGATCCGGACAAAATCGACGAAACGGAAGAGATCCAAGTGAATGGAAAGGAAAAAACAAAGGATGAATTCCATTTTCACTTTAAAGAGACATGCTATAAAAATAGACCACTTTATGAAACTTTTTATCTGGATGGGAATCAAGAAAATTCGAAGTTAGAAATATTGATAGATAAAAAAAATAAAGATCTTTTCTGGTTTGAAAAACCTCTTGTAACTATTCTTTTTGCCTCTCTCCTTTGATGGTGGAAAAACTGGCCCTGTCTCAAGGACTGACTTTTCTTCTTTTCCTAGTGCAGTGACTAAGTATGAGACTAAACTCATTCTCCTTCTTTCGGGGAAAGGGCTGAAGACTAAGGTGGGAGCAGGAAGGGGGATGATCTTTCGGGTCTAGTCCAATTGACGTAGCCTTTGTAGTAGGGGCTTTCCTTTCTTCATTTTTTCTAGCTTCTTTACCGGATAAAATGACAACCGCTTTATTATCGAGGA

>SSH-A74

ACTCTACTCTACGGGGGAAGGGATGGGATTTGGCACGCTGAAACCCTAAGTCCCCTGTTCCCTTTCTTATGATAAAAGAGATTGTTGAGTCCAATTGTGGGTGGAGAAGGCGTCTATGCTTGAAACTGATCCTTAAGATTGGAATCGTTTAGCGTCTATCTATTCCGTGTATTCTTGTGAGGAGAATCCCCCTTTCCCTGGTTTCTATATCTTTATGACGAATAAGTTTATTCCGTTGTCTCTCCCTCTCTCTCTGGATGGATGAGAGACCCTTGCCTGAGGGCACGAAGGAATTAACAAGAAAATCAGGGGCAACTGTCAGCAAATCAAATAAGGGGT

>SSH-A75

ACTTACTTACACTACTTTTAGACCTTCGAATGAAAGTGAAATGGAGATTTCTATCAAAGCAAAGAGAGTTGGAAGGCTGGAAAGCAATAGCTTACAAGATACTCGAGACTAAGGTTCTTAAAGAAGGCAGCCAAATCAGCAATAGAAGAGAACTCCAGATAGTTGAATAGCCGAAAAAGAGCCTAGCTCGATTACTGGAACTAAACAGCAAGCCCCTACCTTACTTATCGAGAAGGGATATTCTATTAGTAAAGTAAAGCACCTTTCTTTATTAGTAATTCGCTCGCTTAAGGAAAAACAGCCTTAAAGGTATAGCTAAAGAAAGGCTGTAGAGAAGAAAGAGAGGGTGGTCGTAGATCAGGCTTCAACTCGAGAAGCAGCCCCCCCAAATCCTCTTCAGAGGAAACCATTCCTTTGGCTCTGGAGTTCATAGTTCGAAGAGCTCGTCCCTCTTGGGAAGCCCTATAGCTTACTTAAGGCGGAATTGGCAAGCGAAGCGGAAGATTAAGGCTAGGAAAGAACTCTTCCAAAAGATATTCTAAACAGCTTTCCGGTCAATCCATGAGATCAAGCACGTAGGGATTTTAGGAAAGAGCAAGCCTTTCTACAGCGAAGGCAAGCAAATGATCTGCATATTCCCTAATATAGAAGTAGCCCATTGGCTTGTTTGGACCGCTCGTATATAAGTATTAGCTCTTAGGGACCACCATCGAAGGTATGAACTAAGTCGAGAGCAACCATGGACTAAATAGAAGGCAAGCAATTG

>SSH-A76

ACCAGGGTGGTGCTGGTCCTGACATGGGTGGCGGCATGAATGAGGATGTCCCATCAGCTGGTGGCAGTGGTGCTGGCCCTAAAATTGAAGAAGTTGATTAAGCTGCAGTTTGAGCAAGAAGTCGCGTTGGATTTTCTGTTTGTAGTTTTGATGGGTGTTTGTTTTTTCCCTTTTCTGTGTGTGGTTTGAATGGGGTTTTCGGAGTGGAAGACGGTGCAGTATTTTGCCCATCCCTCTAGATATGAAGTTCCTCTCTCAGTCTTATGTTAGTGTGCAGTATTTTCTTTTTCTTACTGGCACTCTTTACATGAAGTGATATTTTAATTGAATCATCTAATGGTTGAGATT

>SSH-A77

ACTCGCTCTTAGACTTCCACTTCCACGGCTAAGCCGTCAAGAGAGTTCTTTTTTTCTAAAGAATTCAAGCAATTCGGAGTAGTTCAAACGCTCGGGACAAGGGTAGAAGGGAAGAGGAGTAGTTCAAGCCTGTGTGACCCAAGCGGGAATTCATATTCTTCGCCAAGAAAGGGATTGATCCCAGACCTAGGAGCATGAGACTTAATACGTCTGGTTTAGCGCTATCCTTTCCTTCTGATGAGATGCCTGTTCTCCGGTGCAGATGAAGAAAAGAAAATAAGGTGCCGGTTTTTTAGGAGATTGATTTGGAGACGAATTCATTCCTCTGCTGGAAAGCAGTCCTTCACGGATATGGGAGCTTACAAAGCTCTTACTGGTTTAGTAAGCTAAGCATTTCCTTCCTTTATGATTATGAAAAAGGAATGGATAGAGAGGAAGGCTCCAGGGTTTTTTCTCTTGGTGTCAACATAGGAATTGGAGCAGTTAGAATGGATGCCTTTTCCCTTGTTGAATCAGCCAAGTCAGTAGCCTTTCTTTTATGCGGGATTGCCTGTTGATGCAAGGAATAAGGGCTGGCTTGATGCCAAGAAGAAGCTGGTGGAGGAATAACGGCAGCTAAATCATCTGCTGCACAGTAGT

>SSH-A78

ACCAAAGGCTGCTTTCGCTTGCTTTCATAAGGGGGCTGTTCACTACAAGCTATCAGCGCTGTCTTAGATTGAACGGTAATAAGATAAGTCGACGTTCAATCTCTAAGGCGGCTTTCCGCTGATAACGGAATAGTCTTCGGTGAACAAGGCCCTAGCTTCTAGAGTTCGCTGCTTTTCCCAGGCCGGAAAAGGGCTTATACTGCTCGCCTTTGTTTGATATATTCATTCAGT

>SSH-A79

CTTTCACTAAGATTCTATTGGAAGAAAGTCTAGATTCATCCAACTATTGGAAAAAGTCTAGTCTAATCCAATTTCAGGGTCAAATCCGTTAGGATTGATGTCTCGTAGCGGTGACTGGAATCGATCCGTTATGGCTCGACCCTCTTCCTTAGCAGCTCTATTGGGGTGACCAAGGAGTCTATGCCATTAATCAGACGATGTCAACGTCTCCGATCGGGCTTTCTTTTTCGTCTTCTAATGCTAATCGACCTAAGGACGTGTGGTGTGGATTCAGTGGATTCTGAAGAGAGTCTCTTAGCTGATCTTGACGGAGATTCTCTCTTTTCTATTTTACATAGT

>SSH-A80

GGGGGGAGGGACTTCCTCCTGTCAGATAGAAGCTTTTCAAAGGGTGCTTCGCTTCCACCGGGAGGATTTCCTTGATTGCTCCTATTCCACTACGGCTTTTAAACCTATGAAACTACGGCTATTTGCTCACTGACTCCTATTATTGAGATGGAAACTTTCCGGGTGCGCAAACGAATCACTCCCTTGGAATGCCTCTTTCGAGATTGGAGACTTCTGAAATATCAGCTATATCTGTCGACCCTGAAGTGTGACTTTCCTTGATTGATGAATTGCCCTTTCCGAAACCGTACGGT

>SSH-A81

ACCGAATGGAAGGCTTGCAATCCTATTTAGAAAGAGAATGAAAGTAGTCTCCCCTTAAGGGGCTTCGGTGCCAGTTAGACAGGAAGGAAAGAGAAAGAAGAGATCGGCTTTGGATGATTTTCTAAATGAGAAAGACCCTCACGGAACAAAAACTATAAGGCTCTTTCCATTTTCGGAAACATGCTATCAGAGATAGGGCAATGGGACTAGACAAGGAACAACCCCTTACCCTCGATCTGATGCCCTGACTCTGATTGAAGGAAAAAAAAGGACTTGCTTCGATTCGGGTCAAGCAGGAAGCCAATGCACCTTACTTAGACGAATAAGAAAGCCAATCGCACTTCCACCAATAAGAGGTGCGGGGGATGGTGATACCCCTTGAAAAAAATAACTCACTTACAAGACAGAAAAGTCAACTAACGGAACAAGGATTATCTCGCTCCTTCCTCATAACTAATGGT

>SSH-A82

ACGATTGAAGGGGTTAAGTCCAATCTGTCTGGCCTCGCTTACACCAACAACTAAACTATATGGGTCAAAAGACGGCTTGGCCAAGCAGCGCTCTAGTTAGCTCTGATAGAAAGAAAATCATCATGTCGTCAAGGCAGTTCATGAATGAATGGGAATCGTTGGAGTGTCGAGCGCCCATACCGGCTCAAGGACCAGAATTCGTATAGGATATAATCTTTTTCTAAAAGAATCTTTCTATCGGCGGGGCATCGCGTATTGACTGAGGTTTTTTCGACTTCAATAATAAGTAGAAAGATTGTCGCTACCGCTACTGTAACTGTAAGCAATTTCCATAGTTAAGGGGT

>SSH-A83

GCATAAAGAAATCATGGGCCTAGCTCATAGGGAAAATGATGGGGTGGCCATAATCAAATGGTTCATTTCATAATCCAAAGTTCAGCAAAGGAAATCTAGTTCCCCCCTTCCTCCATGTTGACACAAGAATCCACAAGAATTCTCTTCATATCATCAATAAACTTCTGCATTTGATCCCTTGAATGCAATGGCGATGGATAAACACAAGCAGAATCCAAGCTTCCATCACGTATGGTGTCAAATACGGCGATGGAAGGGCCAACGCCATGGACTGAAGCACAACCCACATAATCCTCAACTCCAAGCTCTCCATACGTTTCATTAGAATCATCAATCACAGGGTCCTCAAACACTGATATGCAGGCAGTTCTCAATGCTGAAGATGGAGTTAAACCTGGATTATCAATTGCCTTACACATTAGGAAGTTGAGGTCACTCATGTCTATAAAGTGCTTGTTGTTGTTCTTTGCATTTGAAAAGGCCAAGTAACATCTCTTTGCTAGCTCCCACAGCTTGTCACCTCCACTTACATCATGTGTATTCATGATGCCAGAGTGGTAAAAT

>SSH-A84

GTTAAACCTTGAGCAGACACTGTGCCTGTGGGAGGTGATGTGGGCTGACCAAGCAGCAATATGTGCAGGGATTGCCAAGTCTGCCTGGGGAAGGATGAGGTTGAGAGCCCCTCCTACTGATGATCTGCTGCTTTATGCAATAGCGGCCTGTGTATTGCAGAGGAGGAAACTGATCATCGAGAAGTATAGCAGTGTTGATGATATCATGAAAGACTGCAACAATATGGCTGGACAATTGGATGTATGGAAGCTTCTTGACGATGCACATGACTTGGTTGTCACGCTGCATGACAAGATTTGGGAAAACTTCCTTTGCTTTGGCCTTGTTTTCAATCTTCTCACTAATTTCCCATTTTCTTTTCATTTGTTATGAACTGCCCCATTTGGATCTCCCTTCTAAAACAATGGCCTCGGGTTCAGCTTTGAAGCTCTGCTCGAGACGAGCATTGATAGGCAATTTGTTTATAGGGCAAATTATGGAGTAATGTATGCTGTGCCCGCACCCAACCGTCCTTGGAGTGATAGTGGCATCATTGCAAAATTACTGTCTTAATGATCTTCTCACTTTCATTTGTATGT

>SSH-A85

ACCTATAGGGAACAAATGGGTTTTCAAGAAGAAAATTGGTTCTGATGGAAAGGTAGAGACCTATAAGGCAAGGCTAGTAGCGAAAGGGTTTCGCCAAAGGCAAGGAATAGACTATGAGGAGACTTTCTCACCTGTTGCCATGCTTAAATCAATTAGGATTTTATTAGCAATAGCTGCATACTATGATTATGAGATTTGGCATATGGATGTCAAAACAGCTTTTCTCAATGGATACATTGAAGAAAACATTTTCATGGAACAACCTAAGGGATTTGAATCCCAAGATGGTTCCAAGGTATGCAAGCTAAAGCGATCCATTTATGGGTTGAAACAAGCTTCGAGGAGTGGAACATCCGTTTTGATGAAGCCATTAAATCTTTTGGTTTTATCAAAAATGAGGATGAGCCATGTGTATATAAGAAGGTTAGTGACAGTGCTATCACTTTCCTTGTCTTATATGTGGATGACATACTGTTGATGGGTAATGATACAGGTATGTTGACGACTATAAAGATATGATTGTCAAATACATTCTCCATGAAAGACTTAGGGGAGGCAACCTATATTCTTGGGATTCGCATCTATAGAGATAGAGAGAAAAGAATAATTGGTTTATCCCAAAGTCTATACTTGGAAAAGGTGTTAAAGAGGTTTAACATGCTTGATTCCAAGAGAGGATTGTTACCAGTGAGACATGGTATCCACCTTTCTAAAGAGATGTCTCCAAAGACACCTGAAGAAAGAGATAAGATGGCCAGGATTCCACATGCTTCGGCTATTGGAAGTTTAATGTATGCAATGTTCTGT

>SSH-A86

ACGGGACAATTTTCAGAGTGCTGCAAGTCTTGCTCAATACCAGCAAGCCCTAAATCATGAGTTTTTCGAAGTTACAATCCTGGAGCTATAAATTCAAGGATAGGCTCTTTAACGCGATGATATCTCTGAGCCGCTAAAATTTTAGATCTTTCGCCTTTCAGTGACATCCAAAAGGAGGCATTCGACTTTATGGTCCGACCCGTGAGCTCTATGCGGCATGACCCTAAACGAAATGTTCTGGAAGGGACACTAAGGCAGTATATTCGGGAGATCGAAGAAAGGGGGAAAAAAGCCTCCATATACAAGGACTTCTATTCGGACTTCACGGACGAGGAATTCCGTCGGTTGATTGACCCC

>SSH-A87

GCTTGTACAAAAACTGCATTTCACCCACAGACAGATGGCCAGTCTGAGAGGGTAATTAAGATCTTGGAGGATATGCTACGGGTTTATGTGATTGAGTTTGAGGGCAGTTGGGATACACACTTGCCTTTGATTGAGTTTGCTTACAACAACAGCTACCAATCAAGCATTGGGATGCCTCCATATGAAGCTTTGTATGGCAGAAAATGTAGAACCCTGTTGTGTTGGGATGACGTGGGTGAAAGAAAGATGATTGGACCCGAAATTGTTTAACAGACTGAAGAAAAAATCAGGGTGATTAGAGATCGACTTAAGACTACATCAGACCGTCAGAAGTCCTACATTGATATGAAGAGAAGGGATATTGAGTATGCAGTGGATGAGAAAGAATTCCTCAAGGTTTCCCCATGGAAGAGGATTATGAGATTCGGAAGAAAAGGAAAACGGAGTCCTTGTTTCATTGGGCCATATGAGGTTCTGGAAAGAGTGGGTCATTTGGCATATCGATTGGCACTACTTCCAGAGTTGGAGAAGATACATAATGTCTTCCATGTGTCTATGTTGAGGAGGTATCGATCAGACCCATCTCATGT

>SSH-A88

ACACTGGGGTTGACTTTTGCAAGAAACTATGTGGGATTTCCATTGTTCGTAGTGGAGAAAGCATGGAAAATGCACTGCGTGCATGTTGCAAAGGAATAAAAATTGGAAAAATTCTTATTCATCGTGATGGAGACAATGGGAAACAGCTTATATATGAGAAGCTTCCCAAGGATATTCCAGAAAGACATGTCCTACTTCTAGATCCTGTTCTTGCTACAGGTAACTCTGCCAACCAAGCAATAGAACTACTCATACAGAAAGGAGTTCCAGAACCACACATTATATTCCTAAACCTAATCTCTGCTCCTGAGGGTATCCATTGCGTTTGCAAAAGGTTCCCATCCTTGAAAATTGTCACCTCAGAGATTGATGCTGCATTAAATGAAGAGTTTCGAGTCATACCAGGTATGGGCGAGTTTGGCGATCGG

>SSH-A89

ACTCATATTATTGCTAGCTAGCTATGTTATTCCTCATTAGCCATCGGCTTTTGGGACGAATTGAATACCTCATTAGCCATCGACTTTTGGGACGAATTGATCTTTGGAACATGATTAAGCTGTGTGTGATTTATTGATATGTATTATGAGATTGTGAAACGGAGTTGAAATTCTTATTGACATTCACTGTCTTTTGAATTTAACCATGATTTGATTTATTGAAATTTATTGTGATATTGAGAAATGGAGTTTAAATTCTTGTTGACATTTACTGTCTTGAATTTAACTATGGTTTTAAGTATCCACTATTTATTTAACTTATGATTTGTGATTTAAAGTTGTATTTGGTTAATGTTGTGCACCACTGAGACATTGTCTCAGCGATAGCTTTTCATTACTATCACAGGTAGGCAGACTAACAGGGCAGCAGACTAGGCTGTTAGT

>SSH-A90

ACAGAACTACAAACTAAAAGAAAAAAAATAGAAGATCGAAATCAAGAAGAGAAAAAACTCAGGCAGAGAAGAACCCATTAGAAGCTGCAAGACCAATTTGGGTCTTGGGCTCCAAAACCATTTTGGCAAGTTCAATATTCTCTTGCCTAACCTTATTCTTGACACTCCGAGACAGGAAAGAAACACAAGAAACCCAGTTAAAAACCTTGATGTCTGAACCTGAATCTTCAGACAACTTCCTTGAATTCTTGACTCTCCTTGAACCCTTGACTCTCCTTTCATACTCCTCAGCAACCAGAATAGCAATGTCAGCCATGGAGATTTGCTCTTTTTTTTTAAGAAAATG

>SSH-A91

ACGTATACGTATGTATATACGGTTATCCTTTCTGAAGTTTTGACGGAAGGATTTCTTTACTTTACTAATGCAACGCAGTCAACTCCATTTATTAGAACAGCTTCCATTGAGTCTCTGCACCTATCCCCCCCTTTTTTTTTATTCTGTCTTTACTTTGTTTGTTTTCGGAAAACAGGATTTGGCTCAGGATTGCCCATTTTTAATTCCGGGGTTTCTCTGAATTTGAAAGTTATCACTTAGTAAGTTTCCATACCAAGGCTCAATCCAATTAAGTCCGTAGCGTCTACCAATTTCGCCATATCCCCTCTTTTTTTGTTTTGAAATTAAGATCTTATTATTATCCCCCTTTTTTCATTTCTATTCTACTGGAACTGTTGAAGTCATTAGATACCGATTCCTATAAAAAGTCTTTCCTCTTATTTTTTATTTCTTGTCTATCTTCTTTTATCTCTATCAGAGACCCTTATTTAGTCTAATCAGAAAGGATTCTATCATTTCTCTATCCGCAATTCAATATAGATGTATATATACCACATTCATTACATATACTTTTCTTGCTCTCATTTTTTTTTTCTCGTGCAATTTTGAATACTCGAACGATCAATTCTTTTTCTTTTTTTTCTATATTCATAATAATTAATTATGAATAACTAAGAATGAAAAGAATAAAAAATTAATAGCCAAGATTCCATTAGTTTCTAAATTCCTATACATGT

>SSH-A92

ACTGTGCCCAAAGTGTATGCCACTGGATACTATCACAATTTGAAAGCTTTCACCAATATATCTGTTTGTTACATATGGTATATTAAGCTTGAGAATTCACAGTTGGGCTGATCGGTTTCTTAGCTGCTTCAATTCTTATATATAGGCCAAAAGAATTTGAGGGCTGATAGGTTTGAAGGCTGATTGGTTTCTCTCTCAAAAACACAATTTTCTTGAGGTGTTGTAGTATTAATTGATTGCAGGATGCAATGGGCTTGATCAATCTTGGAGAGGAACAAGTTCTTGAATTGGGAGAAGGGTATTATTGGCAAAAAAAAAAAAAAAAAAAAAAAAAAAAAGCTTGTACCTTGCCCGGGCGGCCGCTTCGAGCGGCCGCCCGGGGGGTTTTAAAAACCTAAAAAAAATAAAAAAAAAAGGGAAAAAAGAGCTTCTTTCCAAAAAACCCAACTTTCTTTATTTTCTTGTTAAGGCGACCTTTATTTTTTTTTTATTTGGGCATACCCCCCCCCTCCACATAGTTATGAAAATTGGT

>SSH-A93

ACAAGGTTTACTGATCTTGTAAATCTTCTCAAAGCACTTGGTAAAAGATTTGAGGAAGCTGAACTTGTGAAGAAAATTCTTAGATCACTTCCAAAATCTTGGGAAGCAAAGACTACAGTTATCCAAGATACCAAGGATTTTAAAACATTCACTTATGATGAACTCATTGGATCTCTCATTGGACATGAGATGGTATACAAGAAAGATGAAGTTGAAAATGAGCAAAAGAAGAAGAAAAGCATCGCTCTCAAGTCAAATAAGGATGAAGATAAGAAGAAAGGAGTTGCATTCAAAGTTGACTCAAGTGACAACTCAAGTGCTTCGAGTGAAGATGATGATGAAATGTCTATGCTTGCAAGAAAATTTGAAAGGCTTTCAAGAAAGGAGGAAGCAAATACAAGAAATTCTTGAAAAAGTATACTCCCAAGGATAAACACTTCAGAGATTCAAAAGAAGAAATTGTATGTTATGAGTGTCACAAACCTGGACATATCAAGCCCAAATGTCCGTTACTCAAAAAGAAGAAAGGAAAAGAAGATAGGAGCAAGAAAGCTATGAAAGTAGTATGGAGCAACAGTGAAGAATCTTCAAATGATGAATCAAGTAACAAAGAGGCTACTCTTCTTTGTATGATGGCACTTGAAGAGAAAGTCGAGAGTTCACAAAAGGAAGAAGAAAGTGACAATGAGGTAAATTCTTATGAATCTCCTAATGTAGAAGAACTTGAATTTGCATTTGCTAGAGCATATGATGAGTATAGAAATTACAAGAGAAAGTGCACTAAAATGAATTTAGAAATTGAATCATTGAGATCACAAAATATTGCCATGTCTAATGTGTATAAAGAAAATGAATTTTACAAAGGATAAATTGCCTTGTTTAAAGAACTAGGTTTAGAGTTGAAAATCTCAAAAGATACTTGTGAAATGCTTATTGAGAAAAATAAAGTTCTTGAAGCTAAAGTAGAGTCTTTGACAAATGATTTGGCAAAATTTACAAAAGGACAAGAAACTCTAGATGT

>SSH-A94

ACATTAAGGGCTGATCCGTTATTGATGAGGACCTTGGCCACTATACAACCCCTACACTTCACAGTTACATGTAGAGCTTTATTGTGCCCTAAGCCTGCGGGGTCTACCTCCTCTTCAGAAAAGGCTACAAAGCTGGATGCCTGGATTTGTTATACTATCTTCTCAAACTGCCCCGGAGTAATGTCGGGGTTTACAAAGGCCTGATCCAGGATTCTCTGCAAGGCTTGTCGGTGCACTTCCGAGCTCAGGATTAGTGATAACAGTGAAATCCGGGTGGGTGTTTTTCTCAACTGTTCCACCACATCATACTCGCTCTGTTTCATTATTTGGAGCAGCAGTTCTTCCTCTTGCTTATGTTCAGCAGGGTCTACTTCCCCTGCTTTCTCAACCTCCTCTTCTGTATTTTTTGATGACTCTCCCATTTTTACTTTCCCTTTCCTCTTTTCTCTTTCTTCGTTCCCGTAACACCTCCCACTTCGAGTTATAAACTCGACTTCTTGCTCAGATGGAGAGGGCTTTGTTGTGATAACGGGCTGAGGACTAGTTTGGGGAGTAATGTTGCTGGAAGGTCCAGCATAAGTCATCCTGAAATGCTCATGAGGAGCGAAACATGGTTTTGGTGGTGCCGGAGGTGAGGCGAGACTGGGAGCGGACGTACTGCTTGACGCTCCCTGAGCGAAAACTTGGAAATTATAATTCCATGGGACAGCGTGAGTATTGGTGACGGAGAGCTGAGGCGGAGTTCGGATAACCGCTACTGGCGGTGAGGCCACCGGGGGTGAGAAGGTGATCCTGGGTTTGGAGGTAGAAGCCGTTTGGCTGAATCTATGGTGTTTACTCCTTCTTCTGTCTTTGACTTCATCTGGCATCTCAGAACCTTCAGAGACAATAAGTTCCGGACCTCTTGCCTAAACCCCTCACAATCTCGCAGCTCATGGTCAGCCGCCCCTCCATGGTAAGGACACCCCCTCTCTTCGAATCCCGCTACCTGAGGGCAAAGGTAGCCTTCCCTCGTTGCTACAGCAAAAATCTCCTCAAAGTATGGAACCAGCTTATCCACTTCTGGTGTTGATCCCTCTCCCTTAGACTCTATCATATTTACACCACTGCCCGCATTATGGTTAGGCAGTGGGTTTGAGGTAACATTGGGGAGGGAACCATTTCCCTCGATCTTCAACCACCCGTTTCGGATTAAAGCGTGCACTCTCCCTCTGAGTGCCCCGCAGTTATCAGTTGAATGCCCTTGTGCCCCTCCATGATACTCACACCGGGCAGTGGCATCATACCACCTGGGATATGGAGGCTGGATTGGGTCTAAGGGGACTGGT

>SSH-A95

ACTGCATTCCACCCACAGACAGACGGCCAGTCTGAGGGGGTAACTCAGATCTTGGAGGACATGCTACGGGCTTGTGTGATTGAATTTGAGGGCAGTTGGGATACACACTTGCCTTTGATTGAGTTTGCTTACAACAACAGCTACCAATCAAGCATTGGAATGCCTCCATATGAAGCTTTGTATGGCAGAAAATGCAGAACTCCCCTATATTGGGATGATGTGACACGCCTTATCCGTCTACAGTGTAGCCGAGTAAGTTATGTCACTCAGTGTGTCGGAACACTAATTCGTGTTTCAATTTCAATTAATCCCTTTTAATTGATTTATTTAAAATTTTTGATAAATCTAAATTTTTCCGCAAATTTTATAGAAAATCCGGCAGAGTGCCGGCTATAAATTGGAAAAACAGTTCTTCTGAACCTATTAAAAACACTCCCAATAATATCATCACATTATTCTCACTCAACCTCCAATATTTTTCAATCATTTCTCAAATCAATTCAGTATTCCAAAATTTACATTCATCTCATATTCACCAAATTCAATGATAAATACTCATATTTAATTCTGAACACAGTTCATAGATAATTACAACAAAATATTATTACATGAGTTCATAATGT

>SSH-A96

ACAAAGGGAGACCAAAAGTGACTGTCTTCAGTCTACAGTCAATCATAGCATGATGCCTGGCTAACCAATCCATGCCCAAGATAATATCATACTCTCTGAAGGGCATTTCAATCAAGTCTGACAGGAAAACATGTCCTTGGATCACCAAAGGACAGTCTCTATAGATTCTGTTGACCCGGACCTCTTGTCCTAACGGACTAGTTACTAGCACTTCAAAACCCATTTGCACACATGGAACAGCAAGTGAACAAACTATGCTAGCACTAACATATGAATGGGTTGAACCCGGATCAAACAATACAAATACTTCTTGATCAGAGATAGAGAATGT

>SSH-A97

AAATGGGACTGAAAAATACTATGGCAAAGTGAGGTGAAATCTGCCCTATGGACAGCAACATAGGGACTGAAATTTCAGTCCCTTTGCACAGCCATAACTTGGGCTGTGTAAGTCCAATTGAAGTTTGGCCAATTGGACATGAAACTAGGCTTATAATGGCACATTTTTGCTGAAGAAACCATGCCCAAAAGACCAAAGCAAGAGGACCAAAACTTGGCCCCAATCCGGAACCCTAAAACTGCCTCTGCAGAATTTGACCAAATGAACAGTAACTGTTCATTTGGCCATAACTCACTGTAGATATGGTCAATTGACCTGAAATTTTTACAGCAATAAGTTAAGACATATACAAACAACTTTCATGAAGGAACTTACCCCAAATTATGGCCAGAACCTAACCTAAATGGCAGTGGAATTCACTGTTCATGTTACTGTAGATATGGTATTTTCTGCAGAATGAGAATCCGGCCAGCTGTGGTTTTTAGACCATATCTAGAGCCAAAAAACTCCAAATGGAGTGATTCAAAAAAGGAAATTCAACTAGACAAAATAAGAAACAACTTTCATGTTTTACATTTCTTCAAATTCCCACAGCAACAGTGTCCAATGGAACAGTGAAGTTGACTCACCAAAACTGAAAATTCTGCTTGTGTTAGTTTAA

>SSH-A98

AATTCTAAAAACAACTTTTAAAACATCTAAAAAACCCATCAACCCCCAAAAAAAAAAAACACATAGCCAAACACCCTGCTCATAGTCATAATCCATAAAGGGGACTATGAGGATAAGCGGGGCGAACAGACTCATGTTTTGGGTGAAAGGGATCATGCTCTTAAAAAAGTCCAGGCCCTCCAGGCCAATGAAGCTGCTCAGGGCGCTCCCCTTGGTGAGGAGCTTAAGGGAAAAAATGAAAAAATGGGGACAGGAATAGCCGGGGCCTATGGGAACGCTAACAAGGATCTCTTGGCCGAACTCCAAAAGCGTTACCCAAAGGAGGACTTCTCTTGGATGGCCGACCTGGCTCCCGAAGGGGAGGAGGAAAGCGAAAAGGAGGCCGAGGGGGAAAAAAAAAATGAGCAAAATGTAAATAAGGGTGGGGGTGATCCCCCAGCCGAATGACTTTGT

>SSH-A99

TTTTTTTTACCCAAAAAAATACATTAGAAAGGAAAAAACCAAAAAGAAAAAAAGGATAAAAGAAGATACTGAATAACAAATTAACCACAGCCATAGTTTATATCTGGTTTTGCAACTAACGATTCAAAAGGAATTGCAACCCTCTCTTTCACAACGCATCGACCCTCCCCACCTTCCATAACCAGAACTCTCCCAGGTGGACATTCATTGCATCTCTTTTCTTTAGTTGTTGGCTGCTGAAACAAGTCCATCTTATTAAAAGGTATACTTCTTTTCCCAACATTAGGTCTAAAACCAGACAGGCTTAACACTGATATCACACCAAGAGTGATCGCTATCTTAGCAGCTGCACCAATGAAATGTCCTAACCATCTGCTTGAATTTTCAGATGCCTTCTGTGCCTTGCTTTCTGACTCATTCAGATTCTTCTTTGCTTCACCAGCTATTAGCCCATTCCGGACGGACTTGCGTGTATAAGGAAAGT

>SSH-A100

ACCAACCCTAAAAGACTATTGAATGTGACATATTAGTAACATTAAAACCCATTTTACCTAGAACGCATCGAGGGTCGACATCTTAGGTTAACTAAGGAAATAATAAAATTCAGTGAAATAATTATCAAGCATTATAGTTAGAGACAATTTAAATGAGTATTGAAACACTTCAAATTGTGTTTCTCAGTTAGCAAAGACTCAGGGAAGGGAAGAAAACACTGAGTCAGAGCCAAGGGACAGTTATCAGAGGTTTGTGCACAATATTTATTTCTTTTGAATTTTTCAATTGAAATAAATTATGATTATTTATATGTTATTGTTTTAAATTGTGTTTGTGCACAATAAGTATTTCTTTTGAATTTTTCAATTGAAATAAATTATGATTATTTATATGTTATTGTTTTAAATTGTGTTTGTGCACAATAATTTTGATATTTACTGCTTTTACTAGCAAATTTATTTGGAGAAATGAGTTATGAATAATTTTTTATTGCTTTGGTTTTGGGAATATTATTTGGAACTTATTGTGTTGCCAACTTTGCAAATGGAAATTTTGAGATAAAATGTGATTTATGGTTTGTATGAAGTATTGTGATTTGAAATTGTTTTGATTTACACTTGGCATGACACTATTAATATATTCCTCCCTCTTTGACTTATCAGTCTGGGGTGAGTGTGATTATGTTCCTCCCTCTTTGACTTCCCAGTCTGAGGTGAGTGTGGATGAGT

>SSH-A101

ACCTGTTGTGCTTAAGGCTCTAGAGGAGATTGGTTGTCAACTCAGGGAAAACCCACCAAAACAAGTGATTTCTATCAGTCCAACATACAACTATGGCGCAGTTCGACCAACCCAACCATATGTCGACCCAACTTCAGGACAAAAAATCTTGCAGCTGATTATGTAACCCTGGAGATGATGGTCTCGGAGACATTGGTTGGTGGCTTGATCGGTAGATGCGGCTCCAACATATCAAGGATCAGAAATGAGTCAGGAGCAATGATCAAGGTTTATGGTGGAAAAGGTGAACAAAAACATAGGCACATACAATTTGGTGGCACTGCTCAGCAGGTAGCATTGGCAAAACAGAGGGTAGATGAATGCATTTATTCTCAATTGGTGATATCAGCTGATTTGGATTCTCATGCTTGTAAAATGTATGTGTTAACACCCCAGAATTCTGCAGGGGCTCAAAATTAAAATTATAGCAACCAGTTAACTTAACTGAATTATGGTATTGGTTGCTTATTTGAGACAAAAAGTTTGTCATACCAAAAATAATTAAATAATCATGT

>SSH-A102

ACCTGATGCTTTTAAGAACATCATTTCTATATCGAGTTTGACTAGAAATGGCTATGAATTTCAATTCACAGGTGATGTTTGCAATATTTATTTTGGAAATAAATATGTTGGTTTGGGTTATATGAATAATGGTCTTTATTATTTAGATAATAATGACAAACACAAATTGAATGCAAGTGATCTAAAAGAATGCAATGCCATGGTGAAAACCAATTCAAGTTCAAAATATATTTGGCACTTAAGGTTATTTCATGTTGTAGAAGATAGGATTGCAAAATTGGAGAAAATGGAGATTCTATCCTCATTGGGCTCTGAGCTTACTCCAACTTGTGAATCTTGCCTTCAGGGCAAAATGACTAGATCACCCTTTGCTGGACAACGGCTAAGGGCTAAAAATATTTTGGAGCTAATACATAGTGATGTATGTGGTCCATTTAAGGAAATGGCTAGAGGCAGTTTTCATTATTTTATTACCTTTACTGACGATAAATCAAGATTTGGGTATTTGTATTTGATGAAATACAAATATGAATCCTTCGAAAAGTTCAAAGAATTTAAATCTGAAGTAGAAAATCAAATAGGAAAGAGTATTAAAGCTCTTCGATCAAATCGTGGAGGTGAATATTTGAGT

>SSH-A103

TTTCAGTTTTCTTATTTCGATCTACCATTCAGTCGCTGTGATTGATTACATGATTAATTTTTCTGTGATACTTTATTATGATGTCATTGCTCAGTCATAGATTCTTAATCAAGAAAACAATGATTTCCAGAAAAAAAATCAAGAAAACAATGACAAGAGGAGCAACTAATAATTAACTTCATCCAAGTCTCATCTAGAGAGGATAATAATGAATCCATCTCTGAAACATATCATAGCATTCTCTACGGTAGTACTAATACTAGTGAACCATCCGTGCATGCTTTGAGAGTGACTGGGTCGTCATCTCTTATTTCTAGCAGATAAACTTACTGTAACATGGATGGAAATTGGTGGTGGACGCTTAGTCGGATGATGGAGGTTTGTGTGGTGGCTTCTTCGGTGGTGGCTTCTGCACCGGTGGCTTCTTCACCGGTGGCTTGTAGTTTCCGTCTTCCGCATCAACTGCAGGAGGGTGTCCTGGCTTGTGAGGTGGCTTCCCTCCCTTACCTTTGGGAGGCTTGTATGCATCAAGGAATGCCTTCTCCGTATCAACCGAGGAGTGTTCCTCTGCTGGAGGACGCTTGTAAATTGGAGGCTTGTGACCTGATCCTGGCGGTTTGTATTGAGCAAGAGAGTGAGTGGTGAGGAGCACCACCACAACAAACAACACCAGCAAGTATT

>SSH-A104

ACAAGAGCAAAGTATTCCTAAAACTGCCTTCAGAACCCATTATGGCCATTATGAGTTTTTGGTTATGCCATTCGAGTTAACTAATGCTCCGGCTACTTTTATGGATCTGATGAACACTATCTTCATACCATACCTCGACCAGTTTGTTGTGGTATTCATAGATGATATATTGGTCTATTCGAGAAATGCAGAAGAGCATGATAGACATCTGCGGATTATACTGCAGACTTTGAGGGAGAAACAGCTATACGCCAAATTATCGAAGTGTGAATTTTGGCTGAAGGAAATATCTTTCTTGGGGCATGTAGTATCAGAAGAGGGCATCAAGGTAGATCCAAGTAAGATTGAAGCTGTCCTTAATTGGAGGCCACCCAAAAATATCATAGAGATTCGCAGTTTTCTGGGTTTAGCTGGATACTATCGTCGATTTGTGAAGGGATTTTCCATGTTGGCATCTCCGCTGACCAAGCTGCTTAGAAAAGATGTGAAATTTCAGTGGACGGATAAATACCAACAGACTTTTAATAAATTGAAGAAATGTTTGACTAAAGCTCCAGTCCTGACTTTACCTACACCAGGTAAAGAATACACCGTTTACAGTGATGCTTCTTGTTAGTAGTATGCCCTAGAGCATATCATTTAGTATGTATCTTGT

>SSH-A105

TTTTTTGTAACAAGTATTAGCTATTCCCTGATTAATAATACTGGTTAATGATCGAGAAATTAATTTGGCCTGAACCCAGTCATCCTGAAGACTTAAAAATTTGTCTACTGATGGATGCTGCTTATCCTCCTTGACTGAGAGAATAAATGCCGGAAAAATTACAAGAAAGTATTACCTGACAAAACAGTCTTAAACTTCTCTCACATCTGTCATTAATTACCTAAATACATCAGCAAAGCCTCTGCTGGCAATAGTTCACACACTAAGTAGCACACCTTAGGCATTTGAGCAATTTCTTGGCAGCAGAAGCCTCTTGTAAAGCCTCCACAGCAGTAAGCAAAACAATTTCTCCCTGCCATAACACCTCCTGGCCTAATTTCACCAAGTTTGGAGGAAAGAAATCCCATGGATATACTAGTATTTGTTGAAATTATGTCATTTGTTGAACTCAATGTCACCCTTTCACTGGTGGAATCTTCACTTACTTTCCCTAACATAGGAACGTTACTATTGCTGGGAATTTTGGATGGTTTTTTAGCTATCCTAGTGACAGTATTTTTTGTTATAGAACTTCGATTGTCATCAGAGCTACTGCACCTATTCGAACGAATTGGAGAAATAGAAACATTAGGATTGCTGCCATCTAGTTTCACTTCGTTCTTTACAACTGCGGTGTCTGAAATTCCTTCCATTCTTTTATCATCCTTCCCACTCTAGCTTCTTCCCTTGAATGTCAATGATGTGGAGGTAGATGAAATGGT

>SSH-A106

ACAAAACAACAAAAGCTGCAATACGTAAAACTTACTAAAGCCTATTTCCAGTCAGTCATACACCGTTGACAGGATAACAGGCATGAACAGACTCGAGTTGAGAGCTTGATTACCAATTCAGTTGGAGACAACAATTTAATTGATAATACATTGTCTACCAATTCTAGCATCAACTATTCGGTTGACATAAGCATGTAAAATATTTACATTGTCCTGTAAAGCATAAACTCTGATTGATGTAATCTAGCAATGTGGGAAGTGGAAATGCAACCATTCTAAGGTCACATTAGAATCTTCCTTTGCTGTCTTTTTCCATCTAAGCATCCTGAACTAACCAATGAGGTTATCCAAACTTGATCAGACCTAACGGGCTCGTCATCATCATTTCTGTGCCAGGTCCAGTAAGCATGAGTTGAATTCACTAATTTGAGTTCGCCGTGACCAAAACTTGCCTCCCGGAAGACTGACCATTCTGGCTGTGGATTTATGTATTTGGGGGCTAAACCTTCTCTGTTTCCTCCATCTCCAATGGTTATATGAACAGT

>SSH-A107

ACTTGGAATATTTTCAATTATAAGGTTCGAGGTTGATTATGTCCTGGTAGTGGGAGTTTGTTTGAGCCTCAGCATTGCAAACATTATTGGGTTCACCAAATGTCGCAAAGATGCCAAGAAGCAGATTCAAGCATTTGCCTCCCAGACTATTGCTTCTCATTTCTCATCTACCATACAGTCTGCATTTAGTGTAGTGTGAGTTTCCTGAACCGGAAAGGTTATCCGAATACATCATTCTGTTTGCTTAACTAAGATGCTACTATCGACATTAGACTTTTTTGTTTGTTTTCTATCTTCATTGTGGCAACAACTTTGGAATTTCGAAATCTGTTTTATTTTTGGAAGCTGCAAGGACAATGTGATGTAGGCATGTAAAGAAGAGAAGAGGATAAAATTGTAAAGATATTAAAAGGTTTTTGGAATGT

>SSH-A108

ACTTCTTCATCTTGGAAGTCTAACTGAAAGAAGGAGGATAGGGAAAAGGTTGTTAGCAAATATAAGAAAAATGAGGAAAAGGAAAAAAAGGTGGGGAAGAAATTTGTAGAAAGTAAAGGAAAGGAGGTTGCAACTACTACTCGAAATAGAGATGTGAAGTGTTTTAGGTGTTTGGGCAGTGGACACATAGCTTCTCAATGCCCTAACAAGAGAGTCGTGGTCATGAGAGAAAATGGTGAGATAGAAACGAAGGAGGAAATTGTGAGTGATTCCACATCTTCAGGGGAGGAGGATGCAAAGGTAGAATATGCTGCTGAAGGAAGTGCTCTAGTGATTATGAGAGCATTAAATACTCAAGTAAAGGAGGATGTTGGTGATGAATTGCAAAGAGAAACAATTTTCCATACAAGGTGTCTTATCCAAGACAAAGCTTGTAGTATCATCATTGATGGGGGAAGTTGTGCCAATGTTGCTAGT

>SSH-A109

ACATGGAAAGTCTTATATCATGTTATATTGTTAACCTTGAACTATGCTAGCAAATTTCAAACAGTGCTGGGAATGGTGATGTTTTCTGTTGCTTCTGGATGTAATGGGGTAAAGGGAACAATTTCTCATTGAAACTAATGGGAATTTGTTCACATGGTTGCCTCATAGCCGAAAGCGACTAAAGTGTCATACTGAACCGGTGCTTTTGGGACAACTATATGTTGTCTGCTTGTTGTGTGACTATCTAAAGTGTAATGTTGGCTATTGGGAGGCTTACTGCATATTAATTTTATACATTATGTGATGTGTTAAACATTTGCAAGAGCAATATAGCATGAATCATTGATATT

>SSH-A110

ACCTTGATTCACAATCATTGAACTTCTTGCTCAGGTAATAAATGGCCCTCTCTTTTCGGCCGGTATCATCATGTTGTCCCAAAACACATCCCATCGAGTTCTGCTGAACTGCCATATACAGGATCAAAGGCCTGCCCGCCACAGGTGGAACCAATACTGGTGGATTTGACAGATACTGCTTGATTTTCTCAAAAGCCTCTTGACAAACTGAATCCCATTGAGTAGAGTTGTTCTTCCGGAGTAATCTGAAAATAGGCTCAGCTTTGGCAGTGAGATTAGAAATAAACCTCGAAATGTAGTTCAGCTTTCCCAGAAAACTGCGCACCTCCCTTTCTGTTTTTGGGGACGGCATTTCTTGAATAGCTCGGACTTTGTCTGGATCTACTTCTATCCCTTTCTCGGTTACTATGAATCCCAACAACTTCCCCGATCTAGCTCCGAATATGCATTTAGCAGGGTTCAACTTCAAGTGGTATTTCCTGAGCCTTTCGAATACCCTCCTCATCACCTGAACGTGGCTTTCCTTTCCCCTGGATTTAATGATCATATCATCCACATACACTTCCACCTTTTTATGCATCATATCGTGGAATAATGTGACCATTGCGCGCTGGTAGGTAGCACTAGCATTCTTCAACCCGAAAGGCATGACCCGATAGCAAAACACTCCCCATTGAGTGATGAAAGCAGTCTTATCCTTGTCTTCCTCATCCATCGGGATCTGATTGT

>SSH-A111

ACATTAAAAGCTGAACTCCATTCAAACAAGCCCATAAAACCCCATTACATTGCAGATTACACACATCTACTTATTTATTCGGCAAACTGTATAACACACCAAAGATCGAGTTTGCGTAGCATTTCACTCAACCGGAGATCTCAATGGCCTTGACATCTGGTTGCTTGACCTCCTCTTTGGGCACAGTCACTGTCAAGACTCCATTCTCCATGCTTGCCTTAACCTGATCAACTTTCGCATTCTCTGGCAGCCTGAACCTCCTCAAAAATCTCCCACTTGATCTCTCAACTCTATGCCACTTGTCGTTCTTCTCTTCCTTCTCTTTGCTTCTCTCTCCACTTATCTGCAAAACCCTGCCTTCTTCTATCTCCACCTTCACTTCCTCTTTCTTTAATCCTGGAAGATCAGCCTTGAAAACGTGAGCCTCAGGGGTCTCCTTCCAGTCCATGCGTGTGTTGGCAAAAGCCGAAGTTTCGCTTGCCAACTCAGAGCGAGGAGCTGATACAGCTGTGCTTGGGAATGGGAAGTCGTGGAAAGGATCCCAGACGTCGAGAGAGAAGGGATCGAAGATGTTGGTTCTCCGGCCACCGAAGAAGCTGCTTGG

>SSH-A112

GTGCTTAGTGGGTCAGGCTTTTTTCCCCCTTGTTAGCAAGAAAAATGTGTGTTCTTGAAATAAAAAATGTGTGTACGTTCCTCCTTTATAATACTTCTGTGCATGCATTAGTAGATCCTGGATCTACTCATTCATATATTTGTATCAAGCTGCCCGTAGAAAAGGGAATACTAGTGGAGGAAAGTGACCAAGACATCCTGGTCACAAATCCGCTAGACCACAGTGTGTTAGTAAACAAGGTGTATAAGGGCTGTCCATTGAAGATTCAAGGGAAGGAGTTCTTAGCAGACTTAATCGAATTACCTTTCCATGAGTTTGATGTGATTTTGGTAATGGACTGGTTATCCCATCATCAGGCAATAGTAGAATGTAAATTGAAGAGAATCTCTCTGAAAACTGCTGAGAACGAAGAAATAATAGTTATGGGTGAAAGGACAGATTTCTTGTCCAATGTCATCTCAGCTACAGTTGCAAGGAGATGGTTGAGAAAAGGGTGTGAAGCCTATCTGGCACACGAGGTTGACACTAGGCAGGGTAAACTTAACTTATCTGACATACCCACAGTAAGGGATTTCTCTGAGGTGTTTCTAGAGGAATTGCCTAGTTTGCCACCAGAAAGGGAAGTTGAATTTGCTATTGAGATTATACTGGGT

>SSH-A113

TGAGAGTTCTTAAAAGGAACGTGTGGATTTTAAATTTGGGTTGTTTCTTGGAACTTTTGCAGATCGCATTCAAGATCAAGATCTCCACTTCCATCTCGTCAGAAACGCACGAGTAAGAGTCCAAGAAAACAGAGTGTGAGCCTGAGCCAAAGTGGCAGCAGAAGCAGGAGCAGGAGCAGGAGCAGGAGCAGGAGCAGGAGCAGGAGCAAAAGCTTATCCCGGTGAGATGTGAAAATGAGAAATCCACGAGTCAGGAAGCTTGAATGAGCATATGATGCTTAACCTTGCATGATAAATTGTTGATTGCTGAAGGCCATCAAATTCTGGAAAACTTTTTTTTGCGAGACTTTGGGATTTTTATGGCTTGCACATTTGTAGGCTTGATGTGGATTCTGTTTTCAAATTGATAAAGCTTATTTTAATGGATTCGAGTGGCAATATTGATTCACTGTTAC

>SSH-A114

ACAAGTTTATTCAATCGAAGAAATAACAAATGAGAGGGAATGAACTCAATGAACTCAATTGAACAAGTGAATGATGATACCAGCCAATCAGCAATAGCATTATTAACATTAGGATGCTGCATTGTTTGCTGATGCAAATGAATTTCAAAACAATGAAAGGGCGTGTATAATTATGGACATGTTATCATGATTTACACATGCATTCAGGGCACCTTGGTTGTCGATAAATGCTTCGACCAAAGCCCCTAATGCGCACCCTTTGACCTTCTTCAATCATCTTTCTTACTCTATCTTCAAAACTATTCCATCCTATGGTGCTATTCTCTGACAAAATTGCTGCTAGCCTCTTCTTGTTAGGCCTCAAGTTTGGGGCATGTCCACCACCGTAGTAAGTCCGGAAACCAGACACTAGGGATGATAGTTGACTCCCAG

>SSH-A115

ACAGCGTTTGAATCAATAGAGAACCTTTTCTTCTATATCTGTATGAATCGATATTATTACATTCCAATTCCTTCCCGACACCTCCCAAGGAAAATCCCGAATTGGATCCCAAATTGACGGGTTAGTATGAGCTTATCCATGCGGTTATGCACTCTTCGAATAGGAATCCATTTTCTGAAAGATCCTGGCTTTCGTGCTTTGGTGGGTCTCCGAGATCCTTTCGATGACCTATGTTGTGTTGAAGGGATATCTATATGATCCGATCGATTGCGTAAAGCACGCGGTAGCAACGGAACCGGGGAAAGTATACAGAAAAGACAGTTCTTTTCTATTCTATTAGTATTTTCTATTCTAATTCTATTAGTATTTTCTATTCTATTAGTATTCGATTAGTATTAGTTAGCGATCCCGGCTCAGTGAGTCCTTTCTTCCGTGATGAACTGTTGGCACCAGTCCTACATTTTGTCTCTGTGGACCGAGGAGAAAGGGGCTCAGCGAGAAGAGGATTGT

>SSH-A116

ACTTGAATCTTAAGTCGCGGTCATGTCTCGCCCCCCCAGTATACTGACCGGTTCCATGTTTCCCCCGAATTTCATCAGTTCCAGGCTCAAGTGCCTGCTCATCCATCTTCATTCCAAAGGCGAACATTTCCATTGAGTGAGTGTAACTGCTATGGTTTACAGTCAATAGTTCTTAACCAACCCTTTCTCGCGGAGCTTTATAAAGCTTTAAGAGAGAATAGGGAGTAAGGGGACCTTCGCTTCATTAGAAATTTGACTCGGACCTTCTTTCTTCTCCTGCGGAGCCCTGAGAAAGTCACCGGCGGCAGGTTAGT

>SSH-A117

ACTCTTTCTTAGAAAAGACTTGCTGCGCGTTAGCCTGGACAGCAAATCGCCTCAAATACTACATGTTGAATCACAAGACATGGCTTATCTCCAGGATGGATCCTATCAAGTATGTATTTGAAAGCCCATTCATGCCAGGTAGGATAGCCAAGTGGCAGGTTATACTCTCCCAATATGACATAGTCTATATGACCCGGAAAGCGGTGAAAGGTAGCGTGATTGCTGACCTCCTAGCAGAAAATCCTATCCAGGATTATGAAGCTCTGGTTTTTGAGTTCCCTGATGAGCACATTAATGAAGTAGACTGCGAAGAAGGGGGGCCAGCCGATGTATGGGAAATGTATTTCGACGGAGCTGTCAATTTGTCAGGTAATGGAATTGGAGCAGTATTGATATCCCCTGATGGGAAGCACTTCCCAATAGCTGTCAAGTTGAGGTTTGACTGT

>SSH-A118

ACCATTTGACAGAGTGCAGGCTTCAGTTCAAAATTATTAGCTTCTACCCTTGGTCTTGTAATACTTGGCATGAAATCTCCAATGTTAGGATAGGCATGATCCTTCACAGAGCGGTTGTTATTGTTGTTGTTGTTGTTGTCAGCCATTTCTTCTTGTAATTGCTCTTGCTCTTGTGCTCTTTCTTGTTGTTGTTGAGCTTTAATTTCAGCTTTTCTCCTCTTAGATTCTGCTCTTAAAGCCTTTGCTGTCTTCTCTATTTCTGGGTCAAAGAATAGATTGATTTCTTCACTTTTTGTCCTTTTCATAAAATAAAGCACCTGAAAAACAAAAATAAACAAATTCTCAAAGTAAAATTGTAAAAAGAAATAAAATAAAATGCCCAAATTAACCAAACAAACAATTGTTTAATATCAAACAAAAAATCAAATCCCCGGCAACGGCGCCAAAAACTTGGTGTGACTAATCCGCAAGTATACGGGTCGTCTCAAGTAATAAAGTGATGAATCAAGTATCGTTCCCACGAGGATTTGCTGTTTGATTACTAAACTATGGATGAAGCGATTATTTGGGCTAATGATTAATGAATAAATGGTAAATGTAAATGAGCAAGGTAAACTGATTAATCTAATTGAAATGGGTAAAGAGCAAACAAATAAATTCTAATTCTAGTTTGCAATTAAATTGAAATTAACAATGGGTAATTTAAACGAAAGTCTAATTATGGTAAAAGTGATTCCAGAGTTGGGGATTTATGCATAAATTAATTAGGATTTGTCTTGGGCATTCCAATTTTTTAGGGAAAAATAGAGTTTGAAGGAAATTGATTCTAAATCCCTTTAATATCTTTTTCAAGCAAATCAAAGTGTGTTCTAAAATAACCAAACCTACTTTCGTATGTATTTGATTACTTTAAAACCCATTAAGTTTCGTAACCAATAATTAATTCCTCTTAAAGTCCTAGTTTATTTCTAAATCTAGGTGATTTTAAGTTCCAATCCTTGATTAACTATCAATGACTTTCACCTTTCGGTCCTTCAATCAAAGATTAACACAATACCCAATGGGT

>SSH-A119

ACACCATTAACCTCCATAAACGCTTGCATGGATGCACTTTCAAGAAGAAGGCTCCCAAGGCCATAAAGGAAATCAGAAAGTTTGCCCAGAAGGCCATGGGGACAACTGATGTTAGAGTGGATGTCAAGCTAAACAAGCATATATGGAGCCGTGGAATCAGGAGCGTGCCAAGGAGAGTTCGTGTTCGCATTGCACGGAAGAGAAATGATGAAGAGGATGCAAAGGAGGAGTTTTATTCACTTGTCACTGTTATAGAGGTTCCTTCAGAGGGATTCAAGGGTTTGGGCACCAAGGTCATTGATGAAGAGGACTAGAGTAGACTGTTTTCTTGGTTCATAGTTAAAGAACTCCAGTTTTGGTTTTATCTATAATTAATCCTGTGATGTTGGTTTTGTTTTGGATTCTCATTGAAAATCTTTGGCAGTGAAAATATTATTGACTTTTATTAAGCT

>SSH-A120

ACAAAAATATCAGGTGCCTGGTAAGTTACTTCCTCATGTGGATACAATTTGAAGCAAAGTTATAAACCCTGTTATTGAAATCTTATGAAATACTGCTTCCTACATTTCAACATGGTCCCAGTCAATTGATCTATCTTCAAAGCCTTCTGATTGGTGAAGTCTGTTTCGACCTAGTCTGCTCATCATGGCAAGACCTTCAACCATCTTGTAAAGAGCTGACCACCAGCCCTCTCTGGAAATTTGGTATTGATTTAATGATTCATCAATTACTTTGTCATGCTTCCCTTCCAACACTCCTTGCAAAGTTACCACTGCATCCTTTGTTTTCTTCAGGATACTGTCCTCATCCGCAACTTCCAACCGCTGAAGGTCATTATGTGATGAAGTAAGGCTAACAGCAATGGCAAACTGTGCAGCAGCAGCCCAACGAGAAGAGGCCAGCCATTTCCTCTGCCCATCTAACTGTTTGCATACGGTGAGCTTGGCATAACTTGT

>SSH-A121

ACAGGGATATCCTCCGTTGTCCAGTTGTCAAGCTCCTGCTCCTCAGCCATGGGTGAAACTAGGGCCTCATACAAGGAATCCAGTTGGATGACATCTATGGATGGATCATCTGGCGATTCTTCTGTACTTGCCGGATGTTGGATGATCGGTTTGGCGAAGATCTCCGTAATTCTCGGGACTATTTTCATTTTTCCCGTCCCCTGGTCAAACCTCTGATCCCGAAGTATCCTCCTCATCCAGAATCGCCCATCCACGAGGGCATCCTCCTCATACCCCAAACCACAACGGCCTATTTTCTGAATAGCTGGTATAGGCTCGACGATCCCTTGCAAGGCAGTGCCCAAGCCTTTCCCCTCTTGATACCCGTTTCTTGACATTACTTTGGCCACCATAGCCATAGCCCCTTCCTTCCCAGTATCTTGCAATTCAAGGGCCTGGAAAGAACTCTCCAATGATTCCTCGGCCGCTTCCACATAAGGAAGTGATTGCGGCTTGGTGACTAGTATGGCCTCTTCGCCCCTTACTGTGATGATTTTGCCGTCCTTAATGTATTTGATCTTCTGGTGTAAGGTTGAAGGAACAGCGTTTGCAGAGTGGATCCACGGCCTCCCTAATAACATAGTGTAAGCCGGCTCAATGTTCATCACTTGAAACGTGACGTTGAAAGT

>SSH-A122

ACTTGTTGTCGAATGTATTAGGAGATCGAATATCCAGCACGGTTGTATTGGTAGAGTTTGATGTTGGGCAGATTTCCTTGAGATCATTGGCAAATGTCTCGTCCATTACAGGGTCTTGGGTTGGATACAAACGGTTTGTGAAAGAGCTGCAGTGGCTTATTCCGATGGTGTGGCCACCGGAGAGGGCTACCACGTCCGTGGCATCGAAGTTTTTGGTGGCCAGCGATGTGAGAAGTGTGGTGGTGTTGGAAGTGGGTGCTGGCAGGTTTGCCAAGGTTACATTTTGGGTCGCAAAGGTCAACCCGTCTCGCCTTCCCAGTGGAACATCATAATCAGGCCCGCCTGACAAGAAAACAGAATCGCGGGCGGCAATAGCAACAATATCAGCGCAAGAAACGACTCGGCCACACTCCTTGTGTACAAGC

>SSH-A123

AGAGATTTGCATTGAAGAGAATGGATGGAAGCTTGTTGCTGACCTATGATTTCAAGCATACTCAACATATCATGAGCAAATGGGAGTAAGCTTAGATTTTGTCTGTTTTGCCCTACATATATATAAATGTAAATTTATCTTTATAACTTAAAAAATTCCATATATAAGATTGCAAAATACACCATCTTTTAGTGCTTCAGGGAACCTAATAAGTTCTCTTTCATGTAAAGTTATTATAATGTCATAAAATGAATGTATTGTTGATTGGATAAAGACATTCATTTTTCCTCTTCTTCAATGCTATGT

>SSH-A124

ACCACAATGAACACCTGCAATGCTACCAACCTGGGTTGAACTAGACCCTGCAACTGCAGCAGCAATCATTGTTTCTGCTGCTACCCTATCCTTCTCAGCTACTGCTTTAAGCCTGTGCCTCTCAGCTCTAGAACCAATAACCTGCCCTAGGATGGAAGCTCCAATAGTCAGTGCCATAGCAGTTTTAGGCATCACAACAGACTTCCTAAGCATAGCTATGAAGGGCACAGCTGCATGCACTGCTGCAAACCATGATAGTGAAAACTTCTTAGTATGTTCCTTCCACACGCCTAAAGGCACATTTACTGCCATGCCCAAGATTGCAATGACAAACATCTTCGCAGGTAGTGACCGAGGTCGCAAGGTTTTAACCAGGGCAGTCCGTGCAAGGGCAGCCCGTGCCGCAACTACAGCAGGTGGACATCTAAGCGTCACACCAGGCGGCGGTTGAAGAGTTGATGCCACAAGCGGGACTACATGACTAATAGCTCTATGAGACTTTGCAATTGGACAGTTCCCTGTTTCTAACCACTCATTTCCCAGTGACCCATGTTTTGAGGTATTTCCTTTCTTAGAAGATGGATCACTTTTGTTGGTTGATTCAGATTTCTTCTTCTGATTCTTCCACTTATCATGGAACGAACCAAAACCAAAGGGGCCACCAGGACCAAATGCAGATAAACTAATAGTGGCAGCATTTGCTGCTAAGGGGTTGAACTGGGGTGTAAAGTCAGGTTCCAAAATGTCATCATGGAAATCAGACTTGTTTGAGAGC

>SSH-A125

ACGAGGAACAGTTAAAGGCCGGTCTGAGGTTCCCTCTAGACGATTTCTACAAGGAGGTCCTAAAACTTCACCGAGCATCCATTGTTCAAATTCACCCCAACTCGTGGCGGATCTTGGTAGCTTTCCGAGGTCTATGCCGAGCTAAGGGAATCAGACCTACGGCTAAAGTGTTCGCCGAGCTGCACAGACTAACTCGCCGAAAGGATGACGAACACTGGTTCTTTCAGGCGAAGCCTCATTGCGGGCTCTTTTCCGATCTGCTCTCATCCCTTAAGAATTGGAAGAACCGGTTCTTCATTCTGAGGAGTAAAGATCCGAGCTGCTTTGAGGACTTTCCTCGTAGCTGGTGGT

>SSH-A126

ACACTCGCTTGGGTTGAATCGTGTGCTTGGTCAGACTTGCCTTGTTTGAAGCAATGTTTGATTTTCTGTAGGGAAATAAATAAAAGCTTTTAATGAATTTTGAATTTCGTAAAAAAAACTAAAGGTCCTGGTTCGGACGAAGGATACGGTGGCATTTGGAAGCCAAAATGAAATCTACAGAAGGATGGTTGCATCAATTTTATCATGGCATCATTCGATAGTCTGACTGTGGATGACTGGATTGAATGCGCGTTTATGATACCTGTTCATATGGCACATCCAATTTTTCGCATAACCCTAGTGAGGGGGTTCCTACGGGAGTTATACTATTCATTCACAATTTTGCTAAACAATGACCCAAAAGTTTTCCATTAACTGAATATTTATACACAGCATTCTTTACATCGGCCTAGGCTCAGGGAGGCTCTTTATAATGAGATGACATTTTCTGAGATACTCGTATAACCTTTCTTCTTGTTTGGCAGGGTCAAATGCTTTCAGAGCATACTCGGATTCAGGAAGGAGTTCCTGTTTTCCCTGTGCTATCGTTCTCTCCAGCTGGTCAACATTCTCTTTCAGCTCTTGATAGAGAGCAGCTTGTCTTTCTTTCTCCTTCCTTTCCTTAGCACATTCTTTCAGAATATCGTTGATGAGTAATGCCTGTTCCTTTAATTTGAGCTTCTTCTTTTTGTTCTCTTGTTTATGCTCTTCTACGAGTATCTCTTGGTATTTCATTTTTTCCTGAAGCTTACTATTCTGCACTTCTACTTCTTTTACCAGAGCTTGAAGAGAGCTTTTTTCCTTTTCCCACTGCGCTTCTTGCTCTTCGAACCCCATCTTTTCAGTTCTTGCCTCTTCCTCGAGTTTTCTCGCGTTTCTTTTAAGGTTCTGTTGTTGGTCCTCTAATTGCTTTATTT

>SSH-A127

GGGTCTTTAATTTCTATTTGTCCTGCTATTTATTGCAATTTTCTTGGCTTCCATACAAGCTTGCATTTGCCTACCTGCTGTTGGAATGGTTGAATGCAGAGAAATAATATTATGAAATATTGAAAGTCAAGGGGAACACTGTTTCAGAGGAAAAGGTTTGGTGCTTGTTAAAGTAAATGGATATGCTGATCGCATTGTTATTGT

>SSH-A128

ACTACCTCGTATTCAGGGATTGTCTTGGACCCAGTTTATGGAACTGTTTATCAACAGGTTTGTGCCAAAAAGTTTCAGAGATCAAAAGCAGTGGGCCTTTGAGGCCTTAAGGCATAATGGCAGGTCTGTAGATGAATATGCTACAGAATTTCTGGAACTGAGTAGGTATGCCCCTATAGCAGTAGCTACAGAAACTATGAAGGTTAAGAGGTTCCTAAAGGGGTTTGACAGGAGGTATGCAAACCTGGCCATGATGTCTGATCAGTTTTTCGATGTGGTGGTTGATCAAGCCAAACAGATTGAGATTAGCTATGCTGTGGATGATAGAGGAAGGGCAAAGAAAAATAGAGCAGAGGGTTCTTCAAGTCTTTCCGATATGGGTGCTCGGGATAGCAGAGGCCAGCGTAATTACAGAGGAAGAATTAGGAACAAGAAGAGTGGTTTTAGACACAAATCTCGAGGATTCAGACCAGGGT

>SSH-A129

ACAGTGGACCCTTTAGAGGCAGAGCAATGGCTTGAAAGAGTGGACAGAGTATTTAAGAAGCTGCACTGCCAAGATGAACTAAAGTTTGAGTATTCTGTGTCACTACTGTAAGGGGATGCGTATGATTGGTGGAAGACCATCCCCCACAACTTAGCTGAACCACCAGTGCTGACCTAGGATGACTTCATCAGAGAGTTCAGACAGAAATACATCCCAGATGCATATGTTGATCAGAAATTGCAAGAATTTTTGAGTTTGAAACAAGGGAACTGATCAATGGCGAAGTATGAGAGGGAGTTCTCTCGCTTAAGTCATTATGCTGGGAGTCTCCTTACTACCAGCAAGGCAAGATGCAAGAGATTTGAGATTGGTTTGAAGCCCAGTATAAGGATGCAAGTTGTGGGATTCAGACACAACAATTTTTCAGAACTTATGTCTCAAGCACTTGAGCTGGAGAGAATTGAATCAAAAGCAACCCCAGTGAAAGAAAAATCAGAGAAAGCTGAGAAATCAGAAAAAGATAAGGGGGAAAAGTCAGTAGAGCCGAGTTCTGGTGGT

>SSH-A130

ACCTTCTTCCACAGTGACACCTTGAGGAACACTTTATCGCCAACTGCATATTCTATTTCTTTTCTCTTCAAGTCGGCATAGGATTTTTGTCTGTCTGAGGCAACCTTCAGATTAGCTTTGATTAGTTTTACTTTCTCCTCAGTCTGTTTCACCAGGTCTGGCCTTACCAGTTTATCTTCGCCCAATTCAGTCCAGCACACTGGAGTTCTACATTTTCTCCCATACAGTGCTTCATACAGGGCCATTTGGATGCTAGCTTGGTAGCTATTGTTGTATGCAAATTCTGCCAGTGGGAGGTATCTATCCCAACTTCCCTCAAACTCAATGACACAACCCCTCAGCATATCCTCAAGGACCTGACATATATTTCATGTTATTATTATCATTTCAGTTCATTATGTGTATTTGTTTAATTGGTTTCAATTGTTTACCTGGATTACTCTTTCTGATTGCCCATCTGTCTGAGGATGGAAAGCTGTGCTGAAGTGGAGTTGTGT

>SSH-A131

ACTTTTGATCGAAAAATCAATCTGATTTATTTCGCTCAATGAGAAAACAATGAGAAAATGGGTCAGATTCTACAGGATCAAACCTATGGGACTTAAGGAATGACGGAAGGGAATAAAAAAAGAAATAAAAAAAGAAAAGAGAGGGAAAAATAATAAAAATAAATAAAAATGAAGTAGAAGAACCCAGATTCCAAATGAACAAATTCAAACTTGAAAAAATATTTCTGATTCTCGAAGAATGAGGGGGCAAAGGGATTGGTCGAGAAAGATCTCTTGTTCTTATTATAAGATCGTGATTGGATCCGCATATGTTTGGTAAAAAGAAGAATCTTCTCCTTTCAAAAAAGGAAAGTGTTCAATTGGAACATGAAAACGTGACTGAATTTGTCCTAGTTACTCTTCGGGACGGGGTGGATGAAGGGAGGAACTTCTCGAACGAGGAAAAGGATCCAATGACTTCGAAAGAATTGAACGAGGAGCCGTATGAGGTGAAAATCTCATGT

>SSH-A132

AGAGTGCCTTCACCACCGCCACTAGCTAGCTTAGTCTTGTGGCTCGATGGTGGTTATGGGGGAGGAGTTGGAGAGATGGGTTGGTCTGGAGGGGCGTGATTATTGATGGAGGAAGCTGTGGGCGTCAGGCCAATCCGTTTTGATCAGCACGGGAGGTAATCAGTCGGTATCCCTAATCGGAGTAACTGGATACACAAGTTACACTCCGGCATTGGTAATGAGGCAGTTTGGTTCAACACAGACCAGGATCAACGTTGAAGAATACAACCAAGGTCATTTCTACCATGAGCTCATGGAAGTGGAAGGGTTGAAAATGGCTCGCAAGTCTTGGGAAAACATCACTCTGATGGAGAGATCGCCTAAAGGCCCAAGTGCCTCGGGCGATTATCTTAAATGGAGAGCTAACAGGGACCGAGAATACTCAACTACAGAATTCGAAGACAACGATCTGCGCCAAGACGTCCTATTAGAAGAAGCTTATGATCGCCTGGTTGATTCGCTACAAAAGGCAAATACCGAGAACTCACAACGGCAAGAACGAATAAAACAGTTGGAGGACCAACAGCAAACACTTAAAAGAAAGGTGAGAAGGCTCAAGGAAGAAGCAAGGGCCGAAGAGATGGGGTTCGAAGAGCAAGAGGT

>SSH-A133

ACCATGATGTGAAAGATAGCTATTGGTGGAATGGCATGAAAAGAGACATAGCAGATTTTGTGTCCAAGTGCTTGACTTGTCAGAAGGTGAAGTTTGAACACCAAAGACCATCAGGGAAGCTGCAAGAACTCCCTATCCCAGAATGGAAGTGGGAAATGATCATTATGGATTTTGTGACTGGGTTGCCTCGT

>SSH-A134

ACCCATGTGGGGAACACCTGAAGAACCCTCCGCTCTGTTTTTCTTTGCTCTACCGCTGTCATCCACAGCATAGCTAATTTCAATCTGTCTGGCTCGATCAACCACCACATCAAAAGACTGATCAGACATCATGGCCAGGTTCGCATACCTCCTGTCAAGCCCCTTTAGGAATCTTTTCACCTTCATAGTCTCTGTAGCTACTGTTGTAGGGGCATATCTGCTCAATTCCAGAAATTCTGTAGCATATTCATCTACAGACCTGCCATTCTGTCTTAAGGCCTCAAAGGCCCACTGTTTCTGATCTCTGAAGCTTTCTGGCACAAACCGATTGATAAACAGTTCCACAAACTGAGCCCACGACAAACCCTCCATCCGAGTTAACACGTAGTCATTCATCCATTGTCTAGGCATAGGCCCCATGACATGCTACATACACTCTATAAGTCTTCTGTCAGTCAACTGCAGCTCTGTCCCTGCCTGTCTGCAGGAATCCAAAAACCGATATGCATCGTCTGACACATCATAAGTACCAGGCACCAATTTCTTGAAATTTATGATCTGTTTGTAAGGTTCTCCCCTTGGTGCGGTGGACTGCTGCTGTTGTGGAGGGTGGACCATATACTGCGCCATCATATCGATGGTTCTCTGCAGACCAGCTAGAGTAGCTGCCATGGAGTCCATGGGACCCTGTGCCATAAAAGACTGATCTTGT

>SSH-A135

AGAGATACAAATAGTAGATTAACAAGTTTCTTAAACATTATTTACAATATAAAACGCATGCATTGGCAATTTACAGCACAGAACAAGCACAACATCAAATACAAACATTGTTACATTCAGCAGTTGGCATTTTCAGCAGAGTTGCCATGATATCTAATCTTCACTTGTTGAATCGATGTGAGAATCATCTATTGAATTGATCTCAGAACCGTCAAGAACAATTTTAGGGATATGAGCAATCTTGGACCAATCTTCACCTTTTCCACGTTCACATCTTTCGCTCAATTCATCACAATCCTCAATCACAATTTGTTGAAGAGATACTATATTCTGTGGGCACGCAGGCAATTCTTTGACGTTTC

>SSH-A136

CCCACGTCTACGCTCTCTCTCCGGAGTTTCGCTTGGCTGCTGCGCAATTGATCGACAATGACCCGTGGTAACCAGAGAGAGCGAGACCGGGAAAGGGCCCAAGCTAGGGCTGGCCACAAGCCTAAGAATTCCAAAGACGATGGATTGACCCCTAAACAACGCCGTGACAGGGACGCAAAGGCTCTGCAGGAGAAGGCTGCTAGGAAAGCTGCCCAGGCAGCTGTGGGAGGAAACAACGCTGGCGGTGGAGGTAAAAACAATAAGAAGTGATGGGAAGATTGTAAGGGAGGTGTTAGTTAGCCATATGGGGATGTTTGT

>SSH-A137

AATAAACAACCATATAATTAACAATAGAAAACAATAAAGTAAAGAAGAGAACCGGAAAGATCAAATTTAGCAACTATCTAGAGCTTACTTTTAAACTGAGCTTAACTTTTGCGGTTCAAAAAGAGTAGAGCCGCCAGTTTTCTACTCAAACTAACCAAAAGCGCAAAACTACACACATATCTCTCTAGTGCTGGCCTCCCGGCATACAGATCATCTTCCTAGCTAGGTGTCTCTTCAAAGACCATAGTGATGCAAATTCTCAACATCAACCCAGAGGTCTTTACCCATCAACCTCATAATCTTCCTCTGAGCTCTGACTGTGCATCGCGATGGTTTCCCATTTCCTTGGTCATGCTCTACAACGAACATTACCCTGTCATTGGGAACAAGACCCTTCTGCACTACAAAAGGATGCCACGACGAACGGAGAAGAACCGGTTTTGGATATGGCCCTGTGCGACGGGTTGATAGTTGATATGTCCATATGAATCCAGTAATGTCAATTGCTTTAAATTCCTTACCATGAGTTCCTGAAATGCGAGAGCCATCTGTTGCTTCATATTCTCCTGCTCCTTTCATCTCTTCTGTGTCCTCTTCTTTTCTTTCCCCCTTTTTCTTTCTTTTG

>SSH-A138

GCAATTTGCAGTCCAAATATGAATAGGTCGGATCCTATTCCTGTTAAAAAATGTGAGAGGTGCAACTGTGGTGTCAAGGAAATGGAGATTGAGAGCTTGGAGGATTATACTTATGTCACAAGCCATGGACCTGACAAGTCCTTGACTAAAGTGTATTATGATGATGGAAATAAAAAAGGGCGCGATAGAATTGGTGTTGATGTTGTTTCTATACCTAGAGAATCGCCGGCGAGATTTGTCGATGAGTTTGCTATGTATCCCACTTCAGAATTTCTCAGCTCTTGTCACTTGTGCAGGAAAAGGCTCCATGGCAAAGACATATATATGT

>SSH-A139

ACACCAACACTTCTAATTTCTTTCATTTTAGTATTGAATTCTCTGATCCAGTCTGGGTTGTCACTTCCATAAATGCAAAATATTTCTGCCTTCTTCTACCTGAAGTCTCTGAACGGGTGCGATACTCACCTATATGATGATAACAAAAACTGAGTTGTGTCATGAGATGTTCATGAATGCTTCTGACCTTGAGTGCCAAACTTGATAGTTCCCATGCTTCCGTTATCG

>SSH-A140

ACTTTTCTAGCTACTTCTCTAATCTTTGATGCCATCTGTATCCACACATTATTGGCCTCCATATCCAGCTTCCATACTTCGGACTCGAGAAGCTCATTTTTGAACTTCACTTGCTTTACTCCTTTGAACTCCCACCACTTTATTCGAGCTACACTATTTCTTCTGATCTTACTTGAATTGTTCCTAAATTTGACATTCAAGACTACCAATCGATGTTGACTTGTTAAAGCCTCTCCTGGAATGACCTTGCAATCCTTGCATAGAGCTCTATTTGTCTTCCTAGTTAAGAGGAAGTCGATTTGGCTTCTATGTTGGCCACTTTTGAAAGTCACTAAATGTGACTCTCTTTTTATAAAGTAGGTATTTGCTACTATTAGGTCGTATGCCATAGCAAAATCCAGGATGCTTTTTCCCTCCTCATTTCGACTGCCAAAACCAAAACCTCCATGAACATTCTCATAACCTTGTCTATCACTTCCTACATGCCCATTCAAATCTCCACCAATGAAAACATTCTCTTTATTCGGTATGCTTTGCATTAAATCATTCATATATTCCCAAAACCTTTATTTACTTTCACTGTCTAGTCCTATTTATGGGGCATAAGCACTAACTATATTTATTGTTTCTCTTTCTAGT

>SSH-A141

ACACAGATTTCATTAGGCTCTATTAAAACAAAACTCGGTCTTGTGAAGCAGGGATACCTGAACCTGCAAAACTCATTAAAAATGTAAGCTCTCAACTGATTCTGGTTGATTTTTTGTAAAATAATATAATCAAAGCTGGAAAAAGAGAGTTCACCAAAAAACACATCCAATACAGAATGGAAAATTGATTGGTTACTAGCCTGAATGTGTGGTGATCACAGAATCCCAGTGTAATGCTCACACATGTTAAAAACAAGCACACAATGAGAAAACAAAAGAATTTCAAACATCCATGCAATCAACAGTAACAATTCAAATTAGCCACATTTTCATTCTTGGAGAACATAGTTCCGGCTAACACTAGT

>SSH-A142

ATAATTAACTTCATCCAAGTCTCATCTAGAGAGGATAATAATGAATCCATCTCTGAAACATATCATAGCATTCTCTACGGTAGTACTAATACTAGTGAACCATCCGTGCATGCTTTGAGAGTGACTGGGTCGTCATCTCTTATTTCTAGCAGATAAACTTACTGTA

>SSH-A143

ACCATTGACCTAGAGTCACTGGTTACTTGGATTAATTATTCATTATACAAATCATCAGCAATTTGGAAATCAATTCTATATTTGAATGCAGAGCCACAATGCCCATCGAGAAGAAAAGCCTTAAATGGGTCTCCTTAAAAATTAAAATCGTAACTTATTTTGTGGCGAAAGAGCATCATATGATATAGGTGGAGCAGCAACCGTTGCTTGTTGATCAAACTCGGCTGCTGCGTTGCCATAGTTCTTACTGCAAGGAACAATGGTGAGTGATTTCTCATTAGGACATTTCCTGGGCTTGGAGAAGAATTCTCTACACTCTTGTGCTGGTCTTTGATTTGGAAG

>SSH-A144

ACTAAAAAAGTCTGCAGTATAGAGATTCTGACGTGTTATTGGTCATAATTTTTTACAGAAAGCTTCTTTATTTTATTTTTTTTTGTGTGTGAATTCATTTTCATTCCAGTTAGATGTGCATGCACAAAAAGAGCTGTAAATGCTGGATAACATTCCTATATGAAATGAAAATATTAG

>SSH-A145

TCGCACCAGCGCCGGCTCCATAAAATGGTGAGACCGTAGTACTTATGTGTACTACATTCACCTCCACTGTTCGACACCCTCGATCGTTTCTCTACAATGAACCGGAAAAGTTTACGTTTCTTGTTGTTGTTCGTGAAATATCTGTACTCACTGGAGTTGAAGGATTACACATTCCGTTAACTATTAGGTCCAAATTGAGGTAGAAGTCGTAACTCTTGACGGACGTATAGTCACAAACTCCTGGGACACTAACTACTAAGATTACTTTGCATACCTCTCGAACCTCAACTCCTAATACACCCAACACGAAGTCAGGTACCGCAACCGGGAAGGTAGCGGCATAAACTGTGGTATGCACTACCTTCGAACCTAAGACGAACACAAATAGGTAGCGGTAACGTAAGTTCCCTAGTTTACGTCTTCAAATAACTACTATACTTCTCTTAAGAACACCTAAGAACACAGTTGTACCTCCTTCCCCCCTTGATCTAAAGGAAACGACTTGAAACCTAATACTTTACTTGGTAAACTAATACCGGTGGGGTAGTAAAAGGGATACTCGATCCGGGTACTAAAGAAATACGTGGACGGGCCCGCCGGCGAGCT

>SSH-A146

CTTTTAATGCTTAGATAAGAAGAAAATTATTTTATATGGTTTCGTTCTGATCAGTTGTTCAGAACTGTTAAAAAAATTCCATTCTTTTAAATGTAATTTTATTTTAAGAAACATTTGTGGCTGATTCTTAACAAAACAATGATTTATGTTTCTATTTCAGAATATTATCTCTGTCACGAGTATTGATAAATAGAGAAAGTGAAGAGTGATCCTAACCTGAGCTGATTATAACTTGTATAGTAAAGATATTGCTGCATACGAAAGCCTGTCATCTCTTAATGAATTAAATTTGTAAGGGCTGT

>SSH-A147

CAGAAGAGTAAATTATTTTGCTGCCTTAAAATGTATAATTATTTCATGTAATCTTTTTACTCAAAACTTTTGAGGAAAAAAAGAAAAAGAAATTATACAATTTAGTCATGTTTTAAAATATTTGTATCGTAGCTGATGTTGTGTTACGTAGGACTTTTTGTGCACGGAGTTGCAATCTATGTGCAATTTTTTTCTTTTTGGAATTTGGTAAAAAAAA

>SSH-B1

ACCCTAAAACTGTATTCTTGAAAGTTGACATCGATGAGGCTAGGGATGTGGCTGCGAAATGGAATATTAGCAGTGTTCCTACATTTTTTTTCATAAAGAATGGCAAGGAGATCGACAAAGTTGTGGGTGCTGATAAAAATGGACTTGAGAGGAAGATTGAACAATATGCATCCTAGGCCTGAGTTAAATATCTGTAAGAACCTCATAACTTGTGTTTCTTCCGTCCTTTAGGCTCTGTGAGCATCACTAGTCTTACAAATGGGATAAACTTGTGAGAAGATTGTCGTGTTTCATAATAAAATGACCTGCCCTAAGGTATGTTGTATGGATGAAAATAAGCTAATTTTACTCTGCATTTTGT

>SSH-B2

ACTCTGAGGTCAAGCCTGCTCTCAATAACATGGTTGCAGCAGCCAAGCTCCTCCGCACCCAGCTTGCTAGTGCTAAGTGAGTTGTTCAGGAGATGTTGGGAGTATAAACCTCCCAAAACAGTTGATTCTTACTGAGAGGAAAAAGAAAAATGAATTATTGTTGATTCAATAACTGTGGTTTATGATGAAATACCAATTGGGCATGAAGTGCCCAGAGGGCTTTTTTGTTTTTTTTGGCTCATTCAATATTTTGTATTTTGTTTTTGCCTTCAACTTATTTGAAAGGCGTGAAAATTGCTTACTGT

>SSH-B3

ACGACAAGGCTTTGGAAACATACCAGGAAGGTCTTAAACATGATCCTCAGAACCAGGAATTGCTTGATGGCGTGAGAAGATGTGTGGAACAACTTAACAAGGCAAGCCGTGGTGATATAAGCCCAGAGGAATTGAAAGAGAGACAGGCCAAGGCAATGCAGGATCCGGAGATTCAGAACATTCTATCAGATCCTGTCATGAGACAGGTGTTGGTAGACTTCCAAGAAAATCCCAAGGCAGCGCAGGAGCACACGAAGAACCCCATGGTGATGAATAAGATTCAGAAGCTCGTCAATGCAGGAATAGCCCAGATCCGATAGAGTCAAGGCAAAGAACCGATTCCATTATCGTGTAGTTTTTTCATTGCTGAAATGTAATCTAGTGTGTGGATTGTTTGATAAAACTATTCAAGTTGGAAAAATTTTGAATGTTTCTTTTTGTTAAAATATTCACGTTTAGAGATGTTTTTTCTGTTTCACGGTTAGGGTGTAAGTTGAGAACAATTTTTGCGCGCTGAGACTCTCAGCGCGATTGCACGCTTCTTTACTTACTATTAAGAAATAGCACTTTTTGGAAGTATATTAATT

>SSH-B4

ACTCTAGAGTCAAAGGGTTTTAAGTTAAGTAGAAAGAAGACAGAATACATGCATTGCAAGTTCAGTGAAGGCCAAACTGGTGATAGGGAAGGAGTTAGTTTGAATGGAGTGGCACTGTCCCAAAGTAATCACTTTAAATATCTAGGCTCAGTCCTTCAAGTAGATGGGGGATGTGAGGAGGATGTTAGTCATAGGATTAAAGCCGGATGGTTGAAGTGGAGACGTGCCACGGGAGTTTTATGTGATCGTAAGATTCCCAATAAATTAAAAGGAAAATTTTACCGTACAGCCATACGACCGGCTATGTTATGTGGTAGTGAGTGTTGGGCACTGAAAGAGTCGTATGTATCTAAGATAAGAGTTGCAGAGATGAGAATATTAAGGTGGATGAGTGGCCATACTAGACTAGATAAAGTCCGTAATGAGAGTATTAGAGAAAAGGTAGGAGTGGTGCCAATTGAAGATAAGTTGAGAGAAGGGAGATTGAGGTGGTTTGGTCATGTGAAGCGTAGACATACGGAGGCTCCAGTTAGACAAGTAGAGCACATTAGATTAGAGGATAGAAAGAAAAAAAAGGGGTAGACTTAAATTGACTTGGATGAGAGTAGT

>SSH-B5

ACATTACTTGGAGATCAAAATGAAGACAGCGAAATTTACAAACATTATGCCGAATTATGTCAGGGCTCAGCAATGGAACCCTTCCAGCATGATCTGGACAGGGAGGAGCACTATGCCAACATTGTTCGCATGAACATGATAGATGTCATAGATGATGCCACTGTTGAAGCGGAGATGGAAGCGGCTCTGAAGGAGT

>SSH-B6

ACATTCTCTATCTCTGATCAAGATGTATTTGTATTGTTTGATCCGGGTTCAACCCATTCATATGTTAGTGCTAGCATAGTCGGTTCACTTGCTGTTCCATGTGTGCAAATGGGTTTTGAAGTGCTAGTAACTAGTCCTTTAGGACAAGAGGTCCGGGTCAACAAAATTTATAGAGACTGTCCTTTGGTGATCCAAGGACATGTTTTCCTGTCAGACTTGATTGAGATGCCCTTCAGAGAGTATGATATTATCTTAGGCATGGATTGGTTAGCCAGGCATCATGCTATGATTGACTGTAGACTGAAGACAGTCACTTTTGGTCTCCCTCTGTACGGTGATGTGGTAATACACGGGGAGAGGCATTTACTGCCATCAAACATCATTTCGGCTGCACTAGCCAGAAGAATGATCAGAAAGGGGTGTGAAGCATACTTGGCACATGTGATAGACACCCAAGTGGGGAGTCCAGCACTAAGGGACATCCCTACGATATGTGACTTTCCGGATGTATTTCCTGATGATTTACCAGGATTACCTCCAGAAAGAGAAGTGCAATTTGAGATTAATGTTATGCCTGGTGTGGATCCAATCTCCATAACGCCATATAGAATGGCACCTGCAGAATTGAAAGAGTTGAAAGTGCAGTTGCAAGAATTGCTTGACAAGGGCTTTATCCGCCCTAGTGTGTCACCTTGGGGAGCGCCGGTATTGTTTGTAAAGAAGAAGGATGGCACTCTCCGCTTGTGTATTGATTATCGGCAGTTGAATAAGGTGACAATAAAGAATAGATATCCATTGCCCCGCATTGATGACTTGTTTGATCAGTTGAGGGGTGCAGCTGTGTTCTCCAAAATTGACCTGAGATCAGGTTATTATCAGCTGAAAGTTCAAGAGCAGAGTATTTCAAAAACTGCCTTTAGAACCCGTTATGGCCATTATGAGTTCTTGGTCATGCCATTCGGGTTAACTAATGCTCCGGCTGCTTTTATGGATCTGATGAACACTATCTTCAGACCATACCTCGACCAGTTTGTTGTGGTATTCATAGATGATATATTGGTCTATTCGAGGAATGCAGAAGAGCATGATAGACATCTGCGGATTGT

>SSH-B7

ACTGGACGGAACAAACTCGGCGGGCAGGGCCAAGTTAAGAGTAAACCCAAGGATCCATCCAAGCGGCATTATCTGTTCAAAAGACGCTATGGCTAAGATGACAAATGAACACCATAATGGCTGTTCTTGTGTTCTTTTTACATACCAAGAGATGAGTGTGAAGGACCAAAGGTGCTCTCAAACTAGGATTTTCTTACGGTTTGGTATGTATTAAAAAATTATGTATGCTGATGCTATACTTGCAAGATTCCATACAGAAGTATAATGCTTGCAATTACTTCTTTGCACATTCTACTTGAGTGTGCCATATTGAAAGT

>SSH-B8

ACCTGATGCTTTTAAGAACATCATTTCTATATCTAGTTTGACTAGAAATAACTATGAATTTCAGTTCACAGATGATGTTTGCAATATTTATTTTGAAAATAAATATGTTGGTTCAGGTTATATGAATGATGGTCTTTATTATTTGGATAATAATGACAAACACAAAATGAATACAAGTGATCTAAATAAATAAAATGCCATGGTGAAAATCAACTCAAGTTCAAAATATATTTGGCACTTAAGGTTATGTCATGTTGCAGAAGATAGGATTGTAAAACTGGAGAAAATGGGGATTCTTTCCTCATTGGGCTCTAAGCCTACTCCAACTTATAAATCTTGCCTTCAGGACAAAATGACTAGATCACCATTTGTTGGGCAAAGGCTAAGGGTTGAAAATATTTTGGAGCTAATGCATAGTGATGTATGTGGTCCATTTAAGGAAATGGCTAGAGGTGATTTTCATTACTTTATTATCTTTACTGATGATAAATCAAGGTTTAGGTATTTGTATTTGATGAAATACAAACATGAATCCTTTGAAAAGTTCAAAGAATTTAAATATAAAGTAGAAACTCAAACAGGAAAGAGTATTAAAGCTCTTCGATCAGATCGTGAAGGTGAATATTTGAGT

>SSH-B9

ACTTAAATATTATTTAATGGATGAAAACGGGAGAATTGTTAATCCCGATCCATGCAGTAACAGCGTTTTGAATCCATTCAATTTGAATTGGTATTTTCTCCATCATAATTATTGTGAAGAAAGATTCACAATAATTAGCCCGGGACAGTTTATTTGTGAAAATTTATGTATGGCCAAAAAGGGACCACATCTAAAATCGGGTCAAGTTATAATTGTTCACATTGACTCTGTAGTAATAAGATCCGCTAAGCCTTATTTGGCCACTCCAGGAGCAACCGTTCATGGCCATTATGGAGAAATCCTTTACGAAGGAAATACATTAGTTACATTTATATATGAAAAATCGAGATCTGGTGATATAACGCAGGGTCTTCCAAAAGTGGAACAAGTGTTAGAAGTGCGTTCAATTGATTCAATATCAATAAACCTAGAAAAGAGAGTGGAGGGTTGGAACGAGTGTATAACAAGAATTCTGGGAATTCCTTGGGGATTCTTGATTGGT

>SSH-B10

ACTGCTTCTTTTCAAGCTAATCTCTATTTCAACTGCATTCCCTATTGAAAAGAAATCAACCTCGCTTCTTTCTGTTGATAGGTTAGTTTGATTCCAGGTGTGAGCCTCTCCAGTTGCAGTGAAAGTTCCCGACTCTCTAGTGCTATCCCCCTTTTTTCAGCCTGACATTACAAAGGTAGACTTCATCCACCGGACGCCCTGGCTTTCTTTTTAGTTTTCCAATCTTCTGTCCTTCATTCTTGCAGATGCAGATCATAGATGACTACGACATAACTCTTTTGACTTCGTCTCCATATTACGCTCAAGCAAACGGACAGGCAGAAGCTTCAAACAAGGTCCTGATCCGTATCCTGGAGAAAATGATTGAAGATAAGCCAAAAAGATGGGATGGCATCTGATGTTACCAGAAACTTTGTGGGCATACAGAACTTCTAAAAGGGATGCCACAAAGACAAGCCCTTATGCTCTTACCTATGGGCACGATGCCGTCTTGCCGATGGAGATTATGGTTCCTTCACTCAGAGTAGCTAGGCAACATGGCCTTACTCCAGATGATTACACTCAGGCCATGGTGATGGAACTTGAAGACCTGGATGAAACCAGGATGCAGGCCCTCAACCACATGGCTGCACAGAAGAAGAAGATAGCTAGGATCTACAATAATAGGGTCCGAAGACAAACCTTTCAAGAAGGAGACATAGTGTGGAAAACGGTGTTACCTGTCGGTGCCAAAAGTAGAGAGTTTGGCAAGTGGTCCCCGACCTGGGAAGGACCATTCCGGGTGCACAAAGTCCTCAGGGGAAACGCCTATTGATTGTCGAGTCTCGCGGGAGAACTGCACAGAAGT

>SSH-B11

ACCAACTCAAGATATGCTTATTGGGCTCTATGTATTAACAAGCGGGAATTGTCGAGGTATTTGTGCAAATAGGTATAATCCATGTAATCGCAGAAATTATCAAAATAAAAGAATTGACGGTAATAACGATAAATATACGAAAGAACCCTTTTTTTCTAATTCCTATGATGCACTTGGCGCTTATCGGCAGAAAAGAATCCATTTAGATAGTCCTTTGTGGCTCCGGTGGCAGCTAGATCAACGCGCTATTACTTCAAGAGAAGCTCCCATCGAAGTTCACTATGAATCTTTGGGT

>SSH-B12

ACGTAGTATGATGAGCTATACTGATATGCTAATCTCCTTTTGGAGATTTGCATTAGAAACAGCTTTTTATATTCTGAATAGGATTCCATCAAAATTAGTTTCTTCCACACCTTATGAGATATGGCATGGAAGAAAGTAAGTCTTAAGCATGTTAAGATTTGGGGTTGTCCAGCTTATGTCAAAAAGCTGAACACTGATAAATTGGAAACCAGATCAGAAAAAGGTCGATTTGTTGGATATCCAAAAGATAGTTTTGGATATTATTTTTATTTGCCTACTTCACAAAAGGTTGTGATAAGTAGAGATGCCACATTTCTTGAACAACAGTTTGTTCAAGAAGGTGGCAAAGGAAGGCAAATAGAGTTGGAATTGGAAAATTCTGACCAACCAACAGATCAGATGGATATAGATCCATCTAGTCAACCTACACCCATTGATGAAACATCTACAGCTGTTCCTCGTAGAACAACCAGGGTATCTCACCCACCAGTGAGATATGGTTTCCTTCATGAAGAAGAACAAGAGTTGTCTACTCATGAAGAATTAGATCATGGAGATGATCCACTTACCAATGAAGAAGCTATATCAGATATAGACTCTTTAAAATGGATTGATGCTATGAAATCTGAAATTGATTCCATGTATAAGAATCAAGTTTGGGATCTTGTTGACCCACCTGAAGGTATTGT

>SSH-B13

ACAGCCTGTAAAGCTCTTCCCCGGATAAAGACTCTTGCTACAGAACGATTCCTACATTATTTGACTTGCTACCGGAGAGATAAAGAAAGCGAACTCTATTAAGAAACGTAGCTTTTGCCGACACACCATGGAAAGAGACTAGCTTTGAGCCCTTGCCGCCCTAGAAAACATCTATTCTTCTTCTGCTTAAAGGCCTGCAACCTATTCTCATATACATACATTATCGGACCTTTTGAATAAGAGCCAGGAAGACCTTATTCAAATGGAACATTTTCGTATAGAAGATGTAAAACAAATATTGGGAATTCTAGAAATAGAAAAGCATTTTGCAATTGATTTCTCAAAGAATAAATTTTAAATCTTAATGCAGGCATTTCATATCTCGTTGAATCAGTCTTTTTCGCCACATCGCGTATAACGGGAATCGAACCCCCTCTGCTGCTGGCGGGCGGATGGAGAATGTTCTACTTTGATCCAGCAACTTGTTAGGAGTAGGTTTTGGCTTTCCCCTTTTATGTTATTAGTAAAGCGCTAACGAGCAAGAAAACGGATGCGCGTTAGCGCAACGGCTTTCGCGCAGCTCAATCCGTTGCTTGTTCTATAGTAAGGGGCTTTTTCAATAAGGCTCGCGTAGGGGCGCGCACGTTTTTTGGCTCTCTTCGCTCCACTAGCCTTGATTGGCGGATGGAAGT

>SSH-B14

ACGCGGCTAACCTTTCAAATTGAGCTGTTCTTCTTACTCCCATAAACTCAAGAAATTGGGTTGGGTAAGATAAGGACTCCTATAATAAAGCTCTTTTTCTTGCTCTCGTGCTGGATGTCATCTCGGCATATCGGTCGGTTTAAGAGGAATCAGGGGTCATTGGCCGTTGACTTTTTATTCTCTTCTCTGAACTGCTCTTAGGCTAAGATTGAATCCGCTTCTCTTCCGTATTCCTCCGCTCGAGTTTCTGTTGCCGAAGCTGTCTTTCAGCCCTTTCTTTCAAGCTTCCGTGAGTCAGCTCGGTAGCTAGTCTAGTCAAATACATTCTCCTCCCTGAATAGGGGTTGCTCCTTCTTCTCGTCCACTCGGGAATGAAGCCTTCCCTTTCGCTGCTAACCTTTTAAATGGAGTTGAGCTGGCTACTAAATCAATTGCTTCAGTGAAGGTTCCTTCATCAAATCAAATATAGGTGGCAGGGTCAGACAAAGAGTCCACTGCTGTCCAGTCATAGTCAGTAGAAGCAGCGTAGGCTCAGCTCGATCCATTTCTTTTCAATGATTTGATGATAAGCAATTCATGCACAATTCAATTCTTTCCACGCTCGGTTCAAACGAGAGACCTCAAACCAACAAGACGGGCAATCCTCTCGTAGACTGAGCACTATTAGCTGTATATTATTCTGT

>SSH-B15

ACGATGACTTCGTCTGGATGAAGAAGTTTGGTGACCCCATGTTTCAAAGACACATCGCAGCGGCTAGTGTTTGGGGTTTGGTTGCTCTTCAGTTAGCAGATGAGGAGTTTTTACCTTTTAATTATCTATCCTATGCATATGAGCTCCAGGTATGTCTATGTTGCAATGGTTAGAAAAAAAAAATTGAATACAGTTCACAACTTTGTGTTTAATGTCAAAAGTTTACAACAGAAAAGTGCAAAGGACTTGGAAGATGAGATCTCAGATCAAGGCATACGCCTTGATCCCTTATTCAAGTCCATTGACGATCTAACAAAAGCAGCCACCAAAATATATAATGAGAGAAAGGCAATAGAAGAAAGCAGAGGCTGGGGATCAATATTGAAGAAAGACCATTTAAGAGTGAGAGAACTCAATGACAGACTCATGATGGCAGAACGAGCATTTACAGACCAAGATGGACTCTCTGGGAGGCCATGGAATAAGCATTTGATATACGCACCCTCAAAGCATAATGACTATGGATCCAAGTCCTTTCCAGGAATTGATGATGCCATTGACAAGGCAAAGAAGCTCAAAACTCCAGAGTCATGGCACTATGT

>SSH-B16

AGGTTTACCTGGATTACTCGTTCCGACTGTCCATCCTTCTAAGGATGAAAAGCCGTGCTAAAGTGGGGTTGTGTGCCCAAGGTTTCTTGCAACTTTTTCTAGAATCTTGATGTAAACCTTGGGTCTCGATCAGATATGATGGAAAGTGGGATTCCATGTAGGCTAACTATCTCGCTGATATACAATTCTGCTAGCTTCTCCAGTCAATAGTCAGTCTTAACTGGCAGAAAATGTGCTGACTTCGTTAATCTATCCACTATCACCCACACTGCATCATGCTTCTTCTGGGTAAGAGGTAGACCACTAACAAAATTCATAGTGACTCGATCCCATTTCCATTCAGGTATGCTTATAGGCTGTAGCAATCTTGATGGAATTTGATATTCTGCTTTAACTTGCTGACATGTCAAACACTTAGTCACATAGTCAACTATATCCCTCTTCATACCAAGCCACCAATAATGAGGCTTCAAATCATTATACATTTTTGTGCTTCCCGGGTGCATAGCATAAACACTAGAGTGTGCTTCTTTCAGAATACTAGTCTTCAATTCCCCATCATTTGGT

>SSH-B17

ACAAATTGCTGTTAATCGGAGTTTCAACCAAATAACAGTAGATGGAGATACTAGCACCAACGATACAGTCATTGCTTTGGCTAGTGGTTTATCTGGATCAACCCCAATATCATCTATCAACTGCAATGAGGCAGTGCAACTTCAAGCTTGCCTTGATGCAGTAATGCAAGGTCTTGCCAAATCAATAGCTTGGGATGGAGAAGGAGCTACATGCCTAATTGAGGTAATAGTGACTGGTGCAGAAAGTGAGGTGAAAGCAGCAAAGATCGCACGCTCAGTGGCATCTTTTTCACTTGTCAAGGCAGCAGTGTATGGCAGAGATCCAAATTGGGGACGGATTGCTGCTGCTGCTGGCCATGCAGGGATTCCTTTCCACCAGAACAACCTCCACATTATGCTGGGGGATATTTTGCTGATGGATAAGGGGCAACCACTTGCATTTGATAGGGCTGCAGCTGGTAACTATCTCAAGAAGGCTGGTGAGACCCACGGT

>SSH-B18

ACACTCAAGGCTTGGGCTGGCAAGGAGGAGAATCTAAAGAAGGCTCAGGATGCATTCCTCGTCAGGTGCAAGGCCAACTCAGAGGCAACTCTTGGAACTTACAAGGGCGATGCCACTCTTGGTGAGGGTGCTGCAGAGAGCCTCCATGTTAAGGACTACAAATACTAAGAGGTTTCAAGTGTGTTTGAAAGTTGTGCAGAGTAATAGGCTTTCTTGTCTCATGCAATAGTATTTGTAGGGTTTTTTCTATTGTTTCCCTTTCGGCCCAAAAAAAAAAAAAAATTGAAGTAGCGTAATAAAAAGCTGAAGGAGTATTGTTGTGTTGAATTTGAATGGTTTTGGACAGTTTCGGTGGAGAACTGTTTATGTTTTTGTTTCCAACAATTTTAATGAAATTTGAATGATTTGTC

>SSH-B19

ACATAAAAAGGGATTGGAAAACAAATCTTGAAATGTAAACAGAACAAAAAGTTACAGCTTAACAGATTGGAAGAGGGGCACCATTCCATTCCTTGAGACAATCTAGCATTGGATCAATGATCTTTCCCTGGCACATGGCGGTGAAAACCTTATCAAATTCCTCTCCAGGTGACCGGACCTTCTCACCAGTGAGCAACCCAGTTCCTAACTCCTCCCTCACAAACTTGT

>SSH-B20

ACAACCAAAAAGAATGAATAATAAGGAAATTAAGAAATAATGGCAAGGGGAACAACTCAACTCGGGGTATTGTGATATAAAATAATTTATTTAAATTCTATAGCCTAACTATAATTGATTTCTCCATATGGGAGCAATGAATTGAATAAATTATAGAATTTAATCATCTTTATTTTCATCTGGTCTGTAGAGTTGCACTGGACGTCCATTTGTTGGCTGTGTTGGTCTTGCATTTGTGTCGGAGTCATCATGAACCGCTACGCGAAGCAATCTCACTTGTTCTTTGGTAACAGTCCTCACCTTTCTGACAATGGTCCAGAGGACATTTTCAGTGCAAGGAGGAGTTGTAAGAGATCCCATGTATCTATAATACTTTCTACTACCTATCTTGATGTCTTTTGGGTTAACGACACCCACCACTGTCTCTGTTTCTCCGTTGCCAGCCACCATTCTTAAATGGTCTGTGATAGATGACAAGAAAGAGTCTGGCCTTCCTATTATGT

>SSH-B21

GACCCGGAAAAAGAAAATTTTGAAAATAAACTATAAAAATATCAAAATATAAGCAAGGGGAAAAGCCCAAAAGAACAAATAATCCAAAGCTAATGGTACCTAAGGGCAAAATGTTAAACTTCTAACATCTATGAAATAAAAAAACATGGGCCAAGTCAACAAAAAACCCCCCTGGGTCAACAAAACTACTTTTCCCCAATAAAAGGATTTCTAAAGCTCTTAATTTGGGGCCCCCCTTCTTGGATTTCTTAAAAACCCCCAAAGATTGAAATGCCACAACCGGGTGTGCAACTTTTGGTTTTTTTCCCCCCCCTGGGCCTTGGGCCAATCCCCCCCCCCCCCCAACAATTTTTCTTTCCCCGGTTGCCTTGGTCTTTGCCCCCTTAACCCCCAATTGGGCAAAAAAAATCTTCTCTCTTGGCTTCTCCCCTTTTGACTTGTGAATACTTTCCCTAAAAACTCTCTTGTTCTTAAAGAAATTACCTTTAACCTTCAAGTCCTGGCCCGGGGGGGCGCTCG

>SSH-B22

ACAACCAAAAAGAATGAATAATAAGGAAATTAAGAAATAATGGCAAGGGGAACAACTCAACTCGGGGTATTGTGATATAAAATAATTTATTTAAATTCTATAGCCTAACTATAATTGATTTCTCCATATGGGAGCAATGAATTGAATAAATTATAGAATTTAATCATCTTTATTTTCATCTGGTCTGTAGAGTTGCACTGGACGTCCATTTGTTGGCTGTGTTGGTCTTGCATTTGTGTCGGAGTCATCATGAACCGCTACGCGAAGCAATCTCACTTGTTCTTTGGTAACAGTCCTCACCTTTCTGACAATGGTCCAGAGGACATTTTCAGTGCAAGGAGGAGTTGTAAGAGATCCCATGTATCTATAATACTTTCTACTACCTATCTTGATGTCTTTTGGGTTAACGACACCCACCACTGTCTCTGTTTCTCCGTTGCCAGCCACCATTCTTAAATGGTCTGTGATAGATGACAAGAAAGAGTCTGGCCTTCCTATTATGT

>SSH-B23

ACTGTGTTTCATGTGCCATTCCCTCCAGAGTTGGGAGGGTTTGCTCTCGCTTTGAGGGCAGGAACCGTGAGCCCCCCCAGCGCTTCCTCAGGGGCAGGGATGACTTGCCAAAACCTGGACAACCTGGTCAAACTGGTCAAAGAATTTGTCCCGCAGGGATTGGGGCTGCCCCCCGAGCTTAAAAATTTGATTTTTTTTTTTTGATGTTTTGTCAAAAAGTTACTCCTGGGATTCTCGAAAGATTGTTTTGAAATTTCCCTTTTAAATTTGGACCTGCTAAATTTATTAAGGATTATAAATGTTTAAGAACTTGAAAGTTTTTGGTTG

>SSH-B24

ACTGTTTCTATCAGAGGCCGCATACTTGCCGGCACTTGTCACAGTGCAAAGATGATGAGGACCATCATTGTTAGACGCAATTACCTTCATTTTATTAAAAAATACCAGAGGTATGAGAAGCGGCACTCTAATATTCCAGCACACATATCCCCATGCTTCCGTGTGAAAGAGGGAGATCATGTTATTATTGGCCAATGCAGGCCTTTGTCCAAGACAGTGAGGTTCAATGTTTTAAAAGTGATTCCAGCTGGATCTTCTGGTGGTGGAAAGAAAGCTTTCACAGCCATGTGAGTTTCCCAGGGCTTAGAGATGTTGATTGTTTCTGTGAGGGAAAATGACTTGAGATTCGAATTAGACGGTAGACAGTGTTCTTTCAATTTTGT

>SSH-B25

ACGGGAGAGAAAAGAAAAAGGAAATTGAAAACTTTAACAAGACTCTCAGACCAAGGAAAGGAACTGAACTGCTGGAGGAAAACTACCTCAGGCTTACACTAAGGCTTACAATAACATAGAGTAGGGGGAATCTCATGCAAACTGTGAGGGAGGTGCAGATGAATTGAATCAAGAGTAGTGATATCCAGGAATGGAATTGATGCCGAGAATCTGCTCTTTGACACAGAAAATAGCCTATCTAATCAAGGTATCCCAGGCCAAGTTGGGGGCAAATCTCCTATTACAGAATACGCTCTTCTTGTTTGAGAATCAGCTGCGCATGAAGCAGTGTCGCTTATGCTTCCAGACAGCTAAGGCCACATGAACAGAATTACCCTACCCATGATCTAGAGCTTGCAGCAATTATCTTCGCACTGAAGATATGGAGGCACTACTTATATGGTGAAAAGTGCTACATTTACACAGACCACAAAAGTCTAAAATATTTGCCAACTCAGAAGGAGCTCAACCTTAGACAGAGGCGATGGATTGAGTTCCTGAAGGACTATAATTGTGTAATTGACTACCATCCTGGGAAGGCAAATGTAGTTGCTGATGCTTTGAGCAGAAAATCCATCACAGCTTTGAGATCATTGAATGCCCGTCTATCTTTGGTTCGAGATGGAGCTATTTTGGCTGAGTTGCAAGTGAGGCCAAACCTACTACAGCAGATTTTAGATGGGCAAAAGGCAGATGAAAAGTTAATTGCTATTATGAGCAAAATCTCAGAGGAAAAAGTAACTGACTATGAGGTGAAAGCAGATGGGTGTCTGT

>SSH-B26

GAAACCATTATGTGGAGGGTGGTGATGCTGGAAATCGCGAGGATTACATCAACGAGCTCATTAGGAGGATGAATTAGATCCTATTATGTTTGCTTCAGTTATTTTGGTTTCTTTAGCCTCATGAGTTTTGCTGGCATTACAAGTGATATGAGAGT

>SSH-B27

AACCAGTAGTTTCCACCTAGTTTCATCTTTCTTAGTCTGCTGCTGTATTTTTTCCTTCAAATGCCTACAAAGTGGAACCTTGCACGAATCTGGCTCGTTGCACATGCGGGAATGCAGTTCAAGAAGCTGCCACATGCGCTTGCAGTGAACGCATCCACCAGGAACTCGAGTCTTACAATTGGAGAAATGACGAACTAAGTTCTCAAGCCCTTTGCAAGCAGGAAAATTACATGTGATTTGGGTTCCTTTCAGCACTTTGTCACGCGGACCTATTGTCCTGCATCCGTCCCTGCATATGTGAAGGAGGGCCTCCATTGCCTCATAGAGTTGCAAGT

>SSH-B28

ACTAAATTCAAACCAAGAAAACCATCCATTTTATTACCATTTTTTCCATTTTACAGTTGCCATTATTTCTTACAATTGTAATGGCTGAAGAAACAAGGTAATAAAATTCAAGGGCTCATGATAAACCTAGATGCTGATCTCAGAATGGCCCCAGTTTGCCCTTTGCTGCTACTACCTTGCCAACCAGTAGTTTCCACCTAGTTTCATCTTTCTTAGTCTGCTGCTGTATTTTTTCCTTCAAATGCCTACAAAGTGGAACCTTGCACGAATCTGGCTCGTTGCACATGCGGGAATGCAGTTCAAGAAGCTGCCACATGCGCTTGCAGTGAACGCATCCACCAGGAACTCGAGTCTTACAATTGGAGAAATGACGAACTAAGTTCTCAAGCCCTTTGCAAGCAGGAAAATTACATGTGATTTGGGTTCCTTTCAGCACTTTGTCACGCGGACCTATTGTCCTGCATCCGTCCCTGCATATGTGAAGGAGGGCCTCCATTGCCTCATAGAGTTGCAAGT

>SSH-B29

ACCCAAAGCACTGGAGTGATCCAGTAGCCGGGAAGGGGCCTAGAAGTGCCTACTACTATACCACACTACACTTGGCTCTACACATTTACAGAGCTAACCCCTGTCCAGTGCCTGGCAGAGCTAAGGGGCTTCAATCCTTATTCCTTATCCCCCCCAGGCTAAGGAGGCCTTACTTATTCAGGAGGGAGAGTGGAGCCTCGAGAAGCACTGTTGAGAGGAAGATCCTTGGCTCCTCTTCATTCTCTACAGGTTCTTCAACATAGGTGACAAGGAGCGAGGCAGAGATGGAAGAGATCGAACACGGGAATAAGAAAGACTACGTTCCTTTTTGATCATTTTGATAGAGGGGGATGGAGAAAGTTGACAAAACAGACTCGCATTTCCCGGTCGAAAAGATGGGTTCCTTTTTCGTCTCGCATTTTTTCATAGAACAAATACGGGAAAAAAAATCATATTGAAAACGTTCCTAACCCAACCCCTTCCTTCGTAGAGCTGTGTATTGTAAGTGATCCGA

>SSH-B30

TCAGAAAGGTGGTGAAACCAGCACAAACAGTATAGCTTCTATCTTTGCTTGGTCACGAGGTCTTGCACACAGGGCTAAGTTGGATGAGAATGAGAGACTCTTGGATTTCACCCAGAAGCTTGAAGCAGCTTGTATTGGAACAGTGGAGTCTGGCAAGATGACCAAGGATCTTGCATTGATTATTCATGGGCCTAAGGTTACTCGGGACCAGTATCTCAATACTGAAGAGTTCATTGATGCTGTGGCCGCAGAGCTGAAGGCAAAACTTGGAATCAAGCCATAATTGCTTTGGCAGGCAACCTGGAGGAGGAAGAAACCTATGTTATTGGGAAGAAATAAAAGGAGAGGAGAAAGGAAGGGAAAGGTTTAGGGCACAGTTCTACTTTCCGTCTCCTGTTCGATTCAGTTCAATATCAATTTCAATGT

>SSH-B31

ACACAACCTAAACCCAAGTTCTATGTAGCTGCCTGCACGAGTGAAAGCCCGTCGAGTAGATCTCTATAAGAAGCACTCGCCTGCTCAACATAGTTTATCACAAACAAATCATTATAACATTCTGACTCTAATTTCTGATTCCCCAACACATAAAGATGTTCGGTAATTGAGATTGATACTTAAAGCTACTCTAGCCAGTTTCTTCCCTTGGGATGGTCTGCACAGGAACGCTTTCCGCAATATGAGAAGACTGATGCTTAAGCTTATGCTACACCAGCTAAATTTCGCTCTACCGGTGTATAATCCACCGAGGGACATATTCAAGAGATGAAATCACTATTCTGGGGGGAGAAGAGCTATCTCGATCTCTTGAAGTGATTTTCCTTTCGTTTCCATAACATTCCCTTTTACAAAAACCACTGCAATCATGCAAAATGTGGCAAATATAGTGT

>SSH-B32

ACAAGAAGCTGAAAAGAGAAAGCAAGTTCAAGCCAACTCAATCGTAACAAATTATAAATACCTTTTAGGGGTCCCTAGACCATAAACATACATCAATAGCAGCTGAGATCATAAAGCCACAGAAGATCTAAAAACCGTGTATCTTGATATGTTCCTTCTTCACAAGGTCAGCGTTAATAAGGAAACTCTGAACGTTTTTTCGTTGATCTCCTTGGAGCTGGATTACCTCGCCAAGCTCTTTGTCATGT

>SSH-B33

GCTGAGATCATAAAGCCACAGAAGATCTAAAAACCGTGTATCTTGATATGTTCCTTCTTCACAAGGTCAGCGTTAATAAGGAAACTCTGAACGTTTTTTCGTTGATCTCCTTGGAGCTGGATTACCTCGCCAAGCTCTTTGTCATGT

>SSH-B34

ACCCTGACATCGTAGAGAACACCAGAACTGAGACCCTCATTGCAGGAGTTGCACCTCCTAAGAAGTTCTATGAGGGTTCTGAGATGAACTTTGGTGCTGAGAGTGGGAATGGCTGCAAGTGTGGATCAAACTGCAGCTGTGATCCATGCAACTGCAAATGAGAAAGCAGCATGGAAATGGACCCAGCAAAGCTGT

>SSH-B35

ACTCTCTTTGTCAAGCCACCGGCTCCACTAAAGCAGCAAGACTTAGACTTAGCTTAGCCTAGCTAGCACAGACAGAAGGGAAAGCGAACCCGACTGATGAAGTCGAGTGCTCCCACCCGATTCTTCTTGGCTTGACCACTCTCTTAAGCTTTCCCCCATCGAGAAAGAAAAATCCTATGTGAATCCTTACCTCTCACCTCTCCTCTCTACTCATAGCAGACAATCATAGACTTTCAATCGAGTGAATACCCGGCTAGCTCGGATCTAGGTCTATTGTAAAGCTTTCGCTTCGCCCCTTAGTCCAGAGCGATCGCGGGACACGGAATAAGCCTACACATCGGTTTATCCGAGGCGCATGCAGCTTTCATATCCGCACTCCATAATCTCGTAGAGTAAGATGTCCGGTTTACCCCCTATTATCCCGAGACCCGGCTCACCAAGGAGTTTTTTGGGATATACGACTACTTACTAATATGCCTATTCCGATTTCTTTTTCTGGTGTTGCTTGTGCCTGTTGCTGCTCCTGGTCCTCCCATTTACTTCTGCCGAATACGAGAGGTAAAAAGCAAGTCAATCTATTAGCCATCTCCCTTTGGTTTTGATTCTAGGCGCTTTCGGGATCTGTAACGAAACGATAATGAATATGACGGGAGGAAACGAGGCAGTTAACCAAGGGCCGCACAGGGGTGATGCCGGATCCTATTCAACAGAAAATTATGAAGCTGACTCGGGTAGTTGGCGACAGT

>SSH-B36

ACATATGTCGCAAGCGACTTTTCTTCTCCAGCGGATAGAAGTTCTGGATTTTAGAAAAATTGGCTTTTTATGGTTTTTTGAGCTCTTGCTGAATCAGCTGTTCATGCTACCGATTCCTATCCCTAACTTGAAATCTCCTAAGAGAAGTCTTCAATAGAAAAAAATATAAGAAGCGAGTGAATAAAGCAGAATTGATTCAGACTTTCCTCTCGAAGCACTTTATGCTCCAGTCCTACAGTTGGATCAAAAGCCTGTCTTTGTCTTTCTCGGGTTAAGCTAGTAGTCCATTGGGTGGTGGGATCTCCTTATTCCTATTTACCTTGGCTGGGAACTGAAATCAAAGCCTAATCCGGGCTAATATGTCAAAGCAGTCATCTGAAAGCCTTCTCTAATTGTGGAAGAGAAAAAATGCTTTTTTCTTTAAATAGAGCTACTTTCCCTCTTCTCTTATTTCATTCACTCATTTCTCTAATTATCATTACCCCTTACCCTCTCTCACATTCCACCAAGAACTAATAGGCCTTGGCCTTCTACTAGCAAGAGGCCTTTAATTGCTTATATAAAGAGCAGATGGAGATCTTGTTCTTTCTTATTCCCTTATCCGTTCGGGACCCATACCTATGGTTCTCTCTCTATATGAGAATTTACAGGCTCTATTCTCACCTTGCAACCGTCTAGTTGACGAAGCGGAGTTCCTTTTAATACAACTCTTAGATTCTCAAGGTTTAGGAACTAAACTACAACTTTTAGCAAGTTAATCCCTTGTTAAGTTTTAATTTGATATTCATAGTTTTTCATCACATTGTTGATGTTGGGTTGTGAATCCTTGATTGCCTATTGTTTGTTCACTTAGTTTTCTATCACATTGTTGATATTGGGTTAAGTGATAACACTAGAAGAACTTGATTTTGAGCTTTCCTTGGGATGATTTCAAGATTGGTATTGGGTTCCTTAGCAAGTCTAAAGGGGTTTGCATCACCTATCTTTCATGGGTGGCAGT

>SSH-B37

ACATTCAGAGATGGATAAAATTGCTTTTTACCCTTATTTTTATGTAAAGGATCTAGTAGGTTGGGTAGCTTTTGCTATCTTTTTTCCATTTGGATTTTTTATGCTCCTAATGTTTTGGGGCATCCCGACAATTATATACCTGCTAATCCGATGCCCACCCCGCCTCATATTGTGCCGGAATGGTATTTCCTACCGATCCATGCCATTCTTCGTAGTATACCTGACAAATCGGGAGGTGTAGCCGCAATAGCACCAGTTTTTATATGTCTGTTAGCTTTACCTTTTTTTAAAAGTATGTATGTGCGTAGTTCAAGTTTTCGCCCGATTCACCAAGGAATCTTTTGGTTGCTTTTGGCGGATTGCTTACTACTAGGTTGGATCGGATGTCAACCTGTGGAGGCACCATTTGTTACTATTGGACAAATTTCTCCTTTTGTTTTCTTCTTGTTCTTTGCCATAACGCCCATTCCGGGACGAGTTGGAAAAGGAATTCCTAATTCTTACACGGATGAGACTGATCACACCTGATCAGTGAAAAATTCTGACACCAATCATTTACAAGTGAGTATTACACCAAGAATTGACAAGCGGATGAGTTTTCTAGTTGGCTATGTTGATATAGCTTAGATAGGGAAAAGATACTCCTTTTCGTATGCTCGGCAGGTAAGTTTAGT

>SSH-B38

ACACAAATGAAGAAAAAAAATATTGTTTCTAGATATCTCATCAAATTCGAAGCAATTCCCTCTTTTCGATGAATGTCCTAAAGAGCCGCCTCAAATTCGGATTTCAAGATGAAAGAATTCCGTTCTATCAACAAAACAAATATTTCGAAAAAAAAAATGGCCAACTTGCCCATATTCCACAGGAATAATTGAGAAAGGTTTCGGAAGAATATTGTGATGTGATAATCAAAAATGAGTGGCAAAAGCAAAATAAGACAGAAAAGTTATCATTCTAAGTGGTCCAGCCCTTTGTTCATTTTCATTAAGGAGGACAAAAAAAAGATAAAAAGAAACGTCGATTGACAAAAGAAAGGAGAGATAATTTACAAGATATGATCTATCCATATCTAAATCTAACGCATCAATTTCAAATATTGAAAATAAAAAGAGAATTTCTTTATTCTATATTTTATTTTATTCCGAAATTCCAAAACGCTTTGTTTTGCTCTATGAGATGGGTCTATTTTTTCGATTTCTTACTTGTTTTGTTACTGAATTTAGTGAATTTACATATGCATAAAAAATTTTGCAGATAAAAAAGTTTTATTTTCTAATTGTTCCATATATGTATAATATACGCCTTCTTTGGTTCATCAATATTGTAAAGAAATTTCAGTTAAATTATGCTATAGAAAAAAAAGAGGACTTTTGGATTTTTTCGCTCCTGCTAAGAAAATGTTCATTCTTCAGCTCATTTCAAAATACTTCAATTGGT

>SSH-B39

ACTCAAATTGGATCAGTGATCGATTTCTAGGTTTCGTCGTAAACCTAATTGGTTACTTCCAATTACGTAAATCAATAGTTCAAACCGCACTCAAAGGTAGGGCATTTCCCATTTTTATAGGAACTTCTGT

>SSH-B40

ACTTGCTGTCAAGGCCAGCCCTATCGGGAAGGACAGCGCGATGGTTGTTACAGCTGTCAGAATTTGACATAGAATGTGTCACTCCCAAAGCGGGGGCAAGTTTTTCCAGATCTACTGGCCCATCAAAAGACCCTATCGAGCTTTCTGAAGAAATTCCTGGTGAGACTATTCCACTACAGATTGATGGACTTACTAATTTGCTGAATCATCCACAGCCAAGGAAGAAGGTGGTGTAGGAATAGTTTTGA

>SSH-B41

ACCTTCAGTGCTTTCTAACCGAACGACTTGTTGGCTCGGGTTCCTTCTCTCCCTTGAAAGTGAAGACGTTAGCAAGAAGGGCTGATGAAAGAAGATCGGAGTCGTGAACAGGAAAAGCTAAGCCAAAAAGACAGGATTCTCGCCATCTCTTCAGGTCAGGTCAGAGGGGTTGATGGACATGAAATTAGATCTTTTTAGGCTTGAAATACTCCTCTAGGGGAACTCGCAATAGCTCTAACAGAACTTCCAGTTAAAAGCACTTCAACTAGATCGATAGGCTAGCTTGCTGCCTCAGCTCAGTATCTTTCCCTCGCTCGGCCCCAACTGTAAGGAATCCCCTTTTTTTTTGAAACAGGCTCGT

>SSH-B42

ACAGCGCGCTAAGAGAATACAATCGATCGTCCATAAGGGAATGCACCCCGCGGAAAGGAAACAAGGGCTAATTGGCCGCACTCTACCCTGTTATGGGGCAAGAAAGGGATTGACAAACGAAGCACTGGGGATTCAGAAAAGGGAGAGACCATTTGTTAATAAAAGGCATAAAGGTAGCACTGGATAAACTTTCTCCGATCTATGATGATGCCTGCCTTCCCTTTCCCTTCCCTTACTAATGTGGGATCAGTTCCTCCCGAACACCTCCTAGGAGCGTCGCATTCGATGCATATTACTCTCTAAAGTAAGGAGATCTAGCTTACCCGAATAAGCTGGCAAATGCGAAAGCACTTGGAAGAAAGAAAGATGGGTTCAGGCCATGAAGAAGGGCTAAAGGTGGAAAAGAAAGGTGTAGCTGGGCATATCCATGTTTGGGCACTTCACATGATGGGTCTTCCTCTTGCAATCGAGAGAGGCAAATAGGCGGAAATCGGT

>SSH-B43

ACTGTCGCCAACTACCCGAGTCAGCTTCATAATTTTCTGTTGAATAGGATCCGGCATCACCCCTGTGCGGCCCTTGGTTAACTGCCTCGTTTCCTCCCGTCATATTCATTATCGTTTCGTTACAGATCCCGAAAGCGCCTAGAATCAAAACCAAAGGGAGATGGCTAATAGATTGACTTGCTTTTTACCTCTCGTATTCGGCAGAAGTAAATGGGAGGACCAGGAGCAGCAACAGGCACAAGCAACACCAGAAAAAGAAATCGGAATAGGCATATTAGTAAGTAGTCGTATATCCCAAAAAACTCCTTGGTGAGCCGGGTCTCGGGATAATAGGGGGTAAACCGGACATCTTACTCTACGAGATTATGGAGTGCGGATATGAAAGCTGCATGCGCCTCGGATAAACCGATGTGTAGGCTTATTCCGTGTCCCGCGATCGCTCTGGACTAAGGGGCGAAGCGAAAGCTTTACAATAGACCTAGATCCGAGCTAGCCGGGTATTCACTCGATTGAAAGTCTATGATTGTCTGCTATGAGTAGAGAGGAGAGGTGAGAGGTAAGGATTCACATAGGATTTTTCTTTCTCGATGGGGGAAAGCTTAAGAGAGTGGTCAAGCCAAGAAGAATCGGGTGGGAGCATTCGACTTCATCAGTCGGGTTCGCTTTCCCTTCTGTCTGTGCTAGCTAGGCTAAGCTAAGTCTAAGTCTTGCTGCTTTAGTGGAGCCGGTGGCTTGACAAAGAGAGT

>SSH-B44

ACCCAAAGCACTGGAGTGATCCAGTAGCCGGGAAGGGGCCTAGAAGTGCCTACTACTATACCACACTACACTTGGCTCTACACATTTACAGAGCTAACCCCTGTCCAGTGCCTGGCAGAGCTAAGGGGCTTCAATCCTTATTCCTTATCCCCCCCAGGCTAAGGAGGCCTTACTTATTCAGGAGGGAGAGTGGAGCCTCGAGAAGCACTGTTGAGAGGAAGATCCTTGGCTCCTCTTCATTCTCTACAGGTTCTTCAACATAGGTGACAAGGAGCGAGGCAGAGATGGAAGAGATCGAACACGGGAATAAGAAAGACTACGTTCCTTTTTGATCATTTTGATAGAGGGGGATGGAGAAAGTTGACAAAACAGACTCGCATTTCCCGGTCGAAAAGATGGGTTCCTTTTTCGTCTCGCATTTTTTCATAGAACAAATACGGGAAAAAAAATCATATTGAAAACGTTCCTAACCCAACCCCTTCCTTCGTAGAGCTGTGTATTGTAAGTGATCCGA

>SSH-B45

ACTCCAAAGACTCAAATATAGTAAATAAAGAAGGTGGTAAGACCCTTGCAGAGTTCCCCAACAGCATAGAGCTTGGGAAGCTCGGACCCGGGCAAGATCCGAACAACAAGGAGCACTCAACTACTAGTCTAGTCTAGTAGTTGTTTTTTTCTATTAGTTGCGATGCGAACAGGCGTTTACTTATGAGATTAGTTGAGTAGGCTTGCGTTAGTTGTCTGCTATAAGATAGCTAGTTTTGGGGCTTTCGACATAAAAAAGCCTATCCGGCTTGGCTTCGCTATCGCTCATGACTTGTATTGTAGTCGGCCCGGAATGCCTCTGTAGTCTTTCCTCCCGCTGGTCGCCTTCTTCCTTCATTCATTTTCTTTTAGTTTCGGTAGCTTCCGCGCCAGCAAGATACGGACGGCGAAGCCAAAGCAATACTAAACAAGCGAGAAAAGTAAAGCCTAAACGTCCTTATTGAAAAGAAAACTAAAGCGCTAACGAGCAAGAAAAAGGCCCCTTACTATAGATAGGCTAATGCGCCTTTACTAATATTCTAATAGAAGGCCTTTCTTTCTCAAAGTCAAGTTTCGCGCTTCTTACTTTAGAAAGATTGCAGGGGCGCTCACGTTTTTTGGCCCCTTCCCGGCCCGGAAGTTCGCTTCCGGCGACTAGCTTCTACCGCTAGCGCTTGGACTTGGAAGCAATTTCATTGCCTTGAATCAATCAATGAATGAAAAGAAACCGCTTACCGAGGCGACCAGCGGAAGCTCGACTGAGTTGGAGAGGAAGAGTTCGGGAAGGAAAGGTTGGTTCGAGGACCCGTTGGTCAAAGGAAAGGGGAGGTCCTGATTCCGGGACGGAGCCGTATGACGCGAGAGTGTCACGT

>SSH-B46

ACTGCTTCTTTTCAAGCTAATCTCTATTTCAACTGCATTCCCTATTGAAAAGAAATCAACCTCGCTTCTTTCTGTTGATAGGTTAGTTTGATTCCAGGTGTGAGCCTCTCCAGTTGCAGTGAAAGTTCCCGACTCTCTAGTGCTATCCCCCTTTTTTCAGCCTGACATTACAAAGGTAGACTTCATCCACCGGACGCCCTGGCTTTCTTTTTAGTTTTCCAATCTTCTGTCCTTCATTCTTGCAGATGCAGATCATAGATGACTACGACATAACTCTTTTGACTTCGTCTCCATATTACGCTCAAGCAAACGGACAGGCAGAAGCTTCAAACAAGGTCCTGATCCGTATCCTGGAGAAAATGATTGAAGATAAGCCAAAAAGATGGGATGGCATCTGATGTTACCAGAAACTTTGTGGGCATACAGAACTTCTAAAAGGGATGCCACAAAGACAAGCCCTTATGCTCTTACCTATGGGCACGATGCCGTCTTGCCGATGGAGATTATGGTTCCTTCACTCAGAGTAGCTAGGCAACATGGCCTTACTCCAGATGATTACACTCAGGCCATGGTGATGGAACTTGAAGACCTGGATGAAACCAGGATGCAGGCCCTCAACCACATGGCTGCACAGAAGAAGAAGATAGCTAGGATCTACAATAATAGGGTCCGAAGACAAACCTTTCAAGAAGGAGACATAGTGTGGAAAACGGTGTTACCTGTCGGTGCCAAAAGTAGAGAGTTTGGCAAGTGGTCCCCGACCTGGGAAGGACCATTCCGGGTGCACAAAGTCCTCAGGGGAAACGCCTATTGATTGTCGAGTCTCGCGGGAGAACTGCACAGAAGT

>SSH-B47

ACAGCCTGTAAAGCTCTTCCCCGGATAAAGACTCTTGCTACAGAACGATTCCTACATTATTTGACTTGCTACCGGAGAGATAAAGAAAGCGAACTCTATTAAGAAACGTAGCTTTTGCCGACACACCATGGAAAGAGACTAGCTTTGAGCCCTTGCCGCCCTAGAAAACATCTATTCTTCTTCTGCTTAAAGGCCTGCAACCTATTCTCATATACATACATTATCGGACCTTTTGAATAAGAGCCAGGAAGACCTTATTCAAATGGAACATTTTCGTATAGAAGATGTAAAACAAATATTGGGAATTCTAGAAATAGAAAAGCATTTTGCAATTGATTTCTCAAAGAATAAATTTTAAATCTTAATGCAGGCATTTCATATCTCGTTGAATCAGTCTTTTTCGCCACATCGCGTATAACGGGAATCGAACCCCCTCTGCTGCTGGCGGGCGGATGGAGAATGTTCTACTTTGATCCAGCAACTTGTTAGGAGTAGGTTTTGGCTTTCCCCTTTTATGTTATTAGTAAAGCGCTAACGAGCAAGAAAACGGATGCGCGTTAGCGCAACGGCTTTCGCGCAGCTCAATCCGTTGCTTGTTCTATAGTAAGGGGCTTTTTCAATAAGGCTCGCGTAGGGGCGCGCACGTTTTTTGGCTCTCTTCGCTCCACTAGCCTTGATTGGCGGATGGAAGT

>SSH-B48

ACTGGGATACCAGTAAATCCATATGGTTATTCTTTTTTTGAGTCTAGGAGGAATGCCGCTTTCTTTAAAATAACTCAGTTTCCGAACAGATTTGTCGGATATAGATAGATACCTTAACCAAACCCCCCACCTGTTAAATTCAATAAATTGAAGATTGGGTGGAACTCGTTACTCGCGAGTCCCAACTGAGTGATGTTGTTCAATACAGAAGAAAAATTTCCCAGGGTCATATCATCTCCAAGTATATCAGCGACTCTCTGCGCGTAGGTCCAATTCTGAGGTAGAGCCACAGCTTCCTTTCTCTTTCTTTCTTACGTTATAACTAAGTAAGGCAGGCTTCTAGCACTCGCATCATCTTTGAAATTTTCATTTCTTTACCGGGCTTGGACCATGTCTCCCGAACAATCTCAGT

>SSH-B49

ACTAGTTATCTTAGGTCTTAACCAAAGCGAGCAGTCACGGTCGGGTGAACTGAGAACTTTCTTTACTAAGAAACTGGATACCTTACTACTTCAAACCAATACTACACTTTACTTCACGCCTGTCGGCTAAACCAGTAGCAGAACCAACAAAGCAAGCTTTCCTACCCATCAGTTAAGTTACCTTATTCGCGAGAGTTTTGACTGTTCCTACTTTGAAAACCAATCATGAGACGATCTATAATACGCTATTCTTTCTTTGATTCGACAGCGCACAAGGAAAGGGCAGTGCCCCACCAGGTCGTTCGT

>SSH-B50

ACTGCACATGGCATTATGCCATTCTGTGATTTTATGGCTTTTAGCCATTCTGACCTTGTGTTGAGATTTGGCCTTGTGCCTGATGTTATTACAGCTTATTAGGCTGTTCTGTTACACACCGGGAGTGCATATGTGACCGATGGTGTGACGGCCCGAGGTACCACCAGGGCAAGTAAAAGTGCTGGTCTGGTCATCTTTGGGATAACTATACGCATCTCGGC

>SSH-B51

ACATTCTTACAAAATTTACATCAAAAGAAATACAAACTAAACTTTATTACAAACTTCATACAAATTTTTTTACAGGCTGCTCAAGACCCATTTGCACGTCTATACATTTATATGCAATACATACATGAAAAGAAATATTTACAATTAGGGTATAAATTATACCCGAAGACTTTAAGCTGTAGTCCATCCGAACTGGCAGAAAATGAGCAGACTTGGTCAGTCTGTCAACAATGACCCAAACCACATCATGACTCTTCTGTGTCATCGGAAGTCCCATCACAAAATCCATCGTTATTCTCTCCCATTTCCACTCTGGTATTGGTAGTGGATGTAACAACCCAGTGGGT

>SSH-B52

ACAACGAAGAATTGTATGCTGCGTTCGGGAAGGATGAATCGCTCCCGAAAAGGAATCTATTGATTCTCTCCCAATTGGTTGGACCGTAGGTGCGATGATTTACTTCACGGGCGAGGTCTCTGGTTCAAGTCCAGGATGGCCCAGCTGCGCCAAAGAAAAGAATAGAAGAAGCATCTGACTCCTTCGTGCATGCTCCACTTGGCTCGGGGGGATATAGCTCAGTTGGTAGAGCTCCGCTCTTGCAATTGGGTCGTTGCGATTACGGGTTGGATGTCTAATTGTCCAGGCGGTAATGATAGTATCTTGT

>SSH-B53

GAAACCATTATGTGGAGGGTGGTGATGCTGGAAATCGCGAGGATTACATCAACGAGCTCATTAGGAGGATGAATTAGATCCTATTATGTTTGCTTCAGTTATTTTGGTTTCTTTAGCCTCATGAGTTTTGCTGGCATTACAAGTGATATGAGAGT

>SSH-B54

ACCTTGTCTTGGGGCGTCTTTGGTTTTACGAGAGAAAGCCCGCCGAAGAAAAAAGTCTTTCTCTTCCCGTCCCGGATCATATTCCGGTGCGTCCACAGAAGAGGAAATCGCAATGCATCTTGCTCAGCAGGCGTAGCTTGGAGTGGGAGTGTAGCGTGGGTAAGGTAAGGAAAGTGGCCGCCAGAGAGGAAGCCAAACCGGCCTATTAAGCGCAGCTAAGCTAATATGCGCCGGAGAAAGCCAGGTGCCGGAGGTAAGCTCTATTCGGCCCGGAGCATTGATTCCCCATAACGAATAGGCTAGCTCTATCTCTCAATTGCCTATCCTGTGCTCGATAAGGTCCCCCCAGTTCCTCGGCAGCACCTCGAGGTGCATTTAGTTGTCTTACATGAGACTCGGGATATTCCTCACTCCATGGCATGGCACCTTTCGCTCATCTATTCCGCCCGCCCGTCCAGACGCGGCGCCCTTTTTCATTATCATCTACCGCCCTTTCTTTCCTCCCGGCTATTCACGCATGAAGATCGTCGTATGTATGCTCGACTTCGGTTGCCGATGCTTTACTTTAATAGGTAGGAGT

>SSH-B55

ACATGAGATTTTCACCTCATACGGCTCCTCGTTCAATTCTTTCGAAGTCATTGGATCCTTTTCCTCGTTCGAGAAGTTCCTCCCTTCATCCACTCCGTCCCGAAGAGTAACTAGGACAAATTCAGTCACGTTTTCATGTTCCAATTGAACACTTTCCTTTTTTGAAAGGAGAAGATTCTTCTTTTTACCAAACATATGCGGATCCAATCACGATCTTATAATAAGAACAAGAGATCTTTCTCGACCAATCCCTTTGCCCCCTCATTCTTCGAGAATCAGAAATATTTTTTCAAGTTTGAATTTGTTCATTTGGAATCTGGGTTCTTCTACTTCATTTTTATTTATTTTTATTATTTTCCCTCTCTTTTCTTTTTTTATTTCTTTTTTTATTCCCTTCCGTCATTCCTTAAGTCCCATAGGTTTGATCCTGTAGAATCTGACCCATTTTCTCATTGTTTTCTCATTGAGCGAAATAAATCAGATTGATTTTTCGATCAAAAGT

>SSH-B56

ACTGGAAGGTGCGGCTGGATCACCTCCTTTTCAGGGAGAGGTAATGCTTGTTGGGTATTTTTGTTTGACACTGCTTCACACCCAAAAAGAAGGGAGCTACGTATGAGTTAAACTTGTCGACGGAAGTCTTCTTTCTCGACGGTGAAGTAAGACCAAGCTCATGAGCTTATTATCCTAGGTCGGAACAAGTTGATAGGATCCCCTTTTTTACGTCCCCATGTCCCTCCCGCGTGGCGACATGGGGGCGAAAAAAGGAAAGAGAGGGATGGGGTTTCTCTCGCTTTTGGCATAGCGGGCCCCCGGCGGGAGGCCCGCACGACGGGCTATTAGCTCAGTGGTAGAGCGCGCCCCTGATAATTGCGTCGTTGTGCCTGGGCTGTGAGGGCTCTCAGCCACATGGATAGTTCAATGTGCTCATCAGCGCCTGACCCTGAGATGTGGATCATCCAAGGCACATTAGCATGGCGT

>SSH-B57

ACTAGCAGCCTAGTCTGCTGCCCTGTCTGTCTGTCTACCTGTGACAGCAATAAAAAGCTATCGCTGAGACAATGTCTCAGTGGTGCACAACATTAACCAAATACAACTTTAAATCACAATTCATAAGTTAAATAAATAGTGGATATTTAAAACCATAGTTAAATTCAAGACAATAATGTCATAAGGAATTTAATACAATATTACAATAAATCTCAGTAATTAAAACATAGTTAAATTCAAAAGACAGTGAATGTCAATAAGAATTTAAACTCCATTTCACAATCTTACAATACGTATCAATAAATCACACACAGCTTGAAATCATGTTCCAAAGTTCAATTCATCCCAAAAGCCGATGGCTAATGAGGAATAACATAGCTAGCCTGCAAAAATATGAGT

>SSH-B58

CCCAGTTGTGCCCCCAAGGTTCCGCCCCCCCACCCCCCCCCTAAAAAGGCGGAGCAGAAGCCCCCGGAGCCTAAGAAGAAGTTAACAGGAAGCAACCCACCAACAAAGGGGGGGGAAAATGGGAAGAATCCCTGAATTAATTTTTTAATTCATGGGGATTCATATTTGCATCGAGGGATTTGATCCATATTAATGTGTAAATTAAAGCTTTTTGTTTTTTATAACCATGGGTTTTTTTTTAGGGTTCCCCTAATAAAAAATAAAAAAAAACCCCTGTTTTAAAT

>SSH-B59

ACGCTCACTTCTGCATTTTTGTTTAGGTTATCGAAATTATCAATCGCTCTTTTTAGTGGGGAGGAATAGTGCGTGAATGGCGGTTCTCAGACGAGGGAATCATTCCTCTCTAAATAAAAATAGGCTAGAATGCTTCTATTGATAGAACTTTAACGTCTGCGCATAGAATCGTTTTTTTGCCTTTCTATTCCGTTCTTACCGGCAGTTGGATCGGAATCCGTGTGATGATTCTAGAGTGGTTTCAAATATTCTTCGCATCCATCTTCGGCCTTGTGTTAGAAGTCCCGCGGGACTGAGTTAGTGGTCGAGTCGTTTTTGGTAATTGACACCCGTCGCATTAGTTTCAATTCATATGTTACCATTCATAGTGAAGTAGCCCTCTTTTTGATGTCTGAGTGCCTTATTCTATCTATCAAAGTCCTTCTTTGTCTATAGCTCGGGTCAGTCCCCCATGGTCCGCTTTGATTTTCTCGAACAGTTCCGGTCTGCATCAGGGACTCTCGTGCGAGCCATGCAACGTTCCCAGGCAGAAACTGCTCCTTTCCTTGAGCTCCCTATTTAGTTCCTCCCAGGCGTTGATCTCGCAACGGCCCTCCAGCACGTCCTCATATCGCGTCCGCCACCACAGCTTCGCATCCCCAGAGAGATACATTGTTGCCATTGTGACTTGGTCGTCAAAAGGGGCACGAACAGCTTTAATTAAAGT

>SSH-B60

ACTTGGGCACCCAGAAGGAATTGAACTTGAGGCAAAGGAGATGGTTAGAACTGATTAAAGACTATGATTACTTAATAGACTATCAACCAGGGAAAGAAAATGTGGTGGCTGACGCCTTAAATCACAAGACTATAGCAAGTCTCAGAGTTTCTCCTTTGTCTATGGTATATGAGTTGAAAGCATAGCATGCCAGTTTGGAGATTGATGATGAGGGACAGACAGTAGTTGCATGGCATGTACAGCTAGTGTTGATTGTTCAGATCAGAATGGTTGCTCAAAATGATGAAAAATATCAGAAGCTGCTGAAAGAAGTCCAACAGGGCAAGAAACCTGAATTTTCTGTGAGAGATGATGGTTTACTACTACATCAAGGCAGAATGTGCATTCCTAATGATGTTAACTTGAGACAGATCATTATGAAGGAAGCACATGAGTCTACTTTTGCTATGCACCCTGGTGGAACTAAAATGTACAGAGGGCTAAAAGAGCATTACTGGTGGATGGGTATGAAGAGGGATGTAGCAGATTTTGTCTCCA

>SSH-B61

ACTTCAAACTCTTGTGGTTGGTGTATATCTCACACACTTCACCATACAGGTAGTGTCTCCAAATCTTTAGTGCAAAGACTACAGCCGCCATTTTCAAATCATGGGTGGGGTAGTTCTGCTCATGCCTCTTTAGCTGCCTTGAAGCATAAGCCACTACCTTTCCATTCTACATCAAAACACACCATAAGCCAACTCTGGAGGCATCACAGTATACGGTATATCCTTCACCACTCATCGATAATGTTAACACAAGGGCTGTAGTTAGACATTCCTTAAGCTTCTGGAAGCTCTCCTCACAATCAACTGTCCAAATGAATGGAACATTCTTCCAAGTTAACTTAGTTAGGGGAGTTGCTATCCTGGAGAAAATTCTGTAAAAAATGCCTATAGTAGCTAGTTAGGCCTAGAAAACTTTACGCCGTAGTGACTGTTATAGGCCTAAGCCAATCAGTTACCGCCTCAATTTTCTTGGGATCCACTTGAATGCCTTCACTAGAAACCATGTGTCCCAAGAATGAGATGCTTTCTAGCCAAAATTCATATTTTGAAAATTTAGCATATAGCCGGT

>SSH-B62

ACAACTCAGTGTTATGAGCTGTAAAAGCGAACCTTTTTGAATTTGAAATAGGGATTTTCTTACCAGTAATACGAATATGATATGAGTTTAATATTGTAAACTTTCCCTTTTTTAATTTTAAATAGGCATATGCTTTTCTTACAGGAAGAGTCTTTTTTTTCTTTTTTGTATATCGAATTTATGCATATTTTATATATTATTCGTCATTTTTTTTTATATCTAAAGAGTCGCTGAAATGTAAATGTAATTTGACCTCGGTCATCTCGTCATTGGTAGATAGTTGTTTCCACCATCTAACGCTCTCCGGTGTCCACGTTCACTGACAATCACGAGAGACAGAGGGCGAATAAGCAGGCGCATCACTTATGAGAATACTAAGGGGCACACCAACCGAATCTCGACATAAGAAGGAACTAGATCACATGAAAGAAGATTGCTTCCTTCGTATAACTTATCTACTGGCATACCAAACATCTAATCGAGCCTAGAATCCAATCTCACTCACTTATTCACTTATTCTATTTCGAGTTAGCCCTCTCCACTTGCTTTCAACTCTCGTGCGTGATAGGGTCGCGATTACCTTATAGCTAGTTATAGATCGTTGCCCATCATTCAGCGGTTTAGGAATCGTCTTTCGCTACCATGAGATGTAAGCGCAACAACGAAAGTGGTTCACATCACATACGTAATTTCTACAGTTGCTTTTGACTAGTTAAGTTAAGGCTGCTTTACCTACCTCTCCTTAACAGTCGAGCATCGCTAAAGCCCTAATGGAGAACAAGTTCTATTGTCTAGTGATCAGAATGGCTTACCTCCTGCTGGTAATTTGTCTATAATCTTACTCGATATATATTTCAGTATTCTAGACCAGGAGTTTACGCGTGCATACCCAGATTTACCATATTATCGTTTTTTTAACGAAATCTTTATCATTTTTCCGTGTAATCATCTTGAGGAAAATAATATAGAAAAGGATCTATTGGATGCTTTCTTGATGGAAGTCTCTCTGGAAGGAGATGTAAGCATTCTCACTCCTGGGAGTGGATCTACTCAATGTAGAGATGGTAAAAAGATTTGGATAAAAAAGATAGGGTTAATTAAGATTAGGAATACGGAATCATGAATCAATGACTATCTACGACTTCTTTGCTAAACCGGTAGAAGGATGGTAGCGGGCGGGGAACTTTACCAGAAGTCCTATCTATAAAGATAGATTTACTAATCTTACATCTTACTAATGCTAGATTTAAAACTATTCCATTTGACTTTTTCAAGTTCTGTTATTCCTCCTTGCCCGCAGTAGGAAAGAGTGTTCTAGT

>SSH-B63

ACTAGCAGCCTAGTCTGCTGCCCTGTCTGTCTGTCTACCTGTGACAGCAATAAAAAGCTATCGCTGAGACAATGTCTCAGTGGTGCACAACATTAACCAAATACAACTTTAAATCACAATTCATAAGTTAAATAAATAGTGGATATTTAAAACCATAGTTAAATTCAAGACAATAATGTCATAAGGAATTTAATACAATATTACAATAAATCTCAGTAATTAAAACATAGTTAAATTCAAAAGACAGTGAATGTCAATAAGAATTTAAACTCCATTTCACAATCTTACAATACGTATCAATAAATCACACACAGCTTGAAATCATGTTCCAAAGTTCAATTCATCCCAAAAGCCGATGGCTAATGAGGAATAACATAGCTAGCCTGCAAAAATATGAGT

>SSH-B64

ACAAATCTAAAAAGTAGAAATATCTGCTCAACTCTGTAGCGTCTTTTCTTGCTGAGAGCACTTTCAATTTTCTCCGCCAAATCTTTTTTATTTGATATTTTCCGGGAGAGTTGAAGATTATATTTTGTTTGTGGCCTGATAATTGTTTCCGAGACATCCCTAGAGTGCTGGGGACATGGAACTGTTAGTAATGAGATGTTTTCCAAAGATTCTACCCTCAGCTTTCCTAAGCCACAGGGTATTCAATCACCTCGAGCGGCCGCCCGGGCAGGTACTTATTCTGATGAAGCTAGCCTTTCCGCTGGTTTTGATCTTGCTGCATCCATGATCTTCCAGTTCTTCTAGCCCATTTGATTATGGGTTGGCTTGCGAAATAAGTGCTTTCCACCCGAGAAGATCAAACTCGTTTGTTATGACATCTTCTTTATCTATCTTCCCCTCCCCTGGATAGAATGCTTTTCTTTCAGTTTCAGTAGGATTTGTCGAACGGCTTAGTTTAGCGGCAATTTTCCCCACTAGCGTGCTTGCGCAGATGAGCTTCTCCATAAAAAAAAGGAAAGGAATCTCCGCGTATTCATTTCTACGATTTGATTGATAAAGCAGCGCCCAAAGACTGTGCCCATACATAGTAGGCCGGTCGTCTGGCTAAGATAAGATCCAATCTGACACTTTATGAGCAAAAGGCACTCGCTCAAAAGAAGAAGATCTTTCGCTTCAAGTGTGCACTAGCGCTTTGACTTTGAGGAACCAGCGCCCAGGGCGGTTGGTGGGCAGGTCCACGTTTGGCTAATCAGACTCTTGGACATGAATAAAAGCATTCAATCAATCTGACCAGCAAAGAGTCTCTTTTTCTTCTTTTTTTAGGGACGT

>SSH-B65

ACCAAATTAATATTTGTTCCTCCATGGGAAGGATTCTCTCCCAATTCTGCTAGGTCTAGTGTAATGGCAAGCATAATGGGACATGATAGACAGAGCAATTTGCAGGATAATGCAAGGAGTTTGAGTTATGCAGACAGTTTGAAGATGCTAATTCAGAATTTTGACTTTTCTTACCGGCTAGAATGGGTTGGGCAACGTAAGGTGCTTCTTACTAGGCATGGCATGGAATTGGGCACTTTCCCTTTATGAACAGCAGCAGGTAGTGAAGCACATTCTTACGGGCGAGCTTCAAGAAGCATGCAGTGAGGGATGCACTCATGGTTCGT

>SSH-B66

ACCCTACGAATCCATAAATGATACTGGTATGTTCTATGGTATGAAGTTTTACAATGACCTTCTAATGGAAGCTGGACCTTTTGGAAATGTCCAGTCTGAGGTGCTTCTACGGAAAGACAAGAACACTTTTACCCTGAAGCAGGGGTGGGGATTTCCCAGGAAAGTTTATTTCAATGGTGATGAATGCAAGATGCCACCACCAGATGCATACCCATATCTGCCAAATTCTGCCTATGCAAATCCAGTTGCATTTTCAACAATGGCAGCTTCTTTGCTTTTGATGTTACTTTCAATATGGTGATCTATCTTCTATGGGGGAAATTTTATTCTTCAGATCTCTTTGTAACAGATAATGCAGATGCAGTTTAGGAAGCCAGAAAATTTTTTTTACCACAGGTATATATATCAATGTTAACTCTCATATACAAGCTAGAAAGGAGGATGTGCTTTTGAGTGTGGGAT

>SSH-B67

ACGTGTAGTCACTCATACACGCTATATGAGGTCATAATTAACATGCGAAAAAGTCATAGGATAGGCTGGTTCATGCTTACCGAAGACAAGATATTCTTCTTTACTTCCTTCTTTCGGACTAATGCGTCTGCAGCCGTTGCAGCTAAATAAACGGAGGAAGGAGGAGGCCCCAAGCATCAAATGGATTAGAGTTTGAGAAGAACTCAAAGCGGTTCGATGTTCTTAAGCAAAGAGTGCCTTGATCAGAATCAAGGGTATGCTATAAATGAGAGAGAGAGCAATGGGAAAGAGAAGGGATGATGCAGTCAATAATGACTTACTTATAGGTAAAGAGAGAATCTTCTTACCGAGTCGAATGAGATGT

>SSH-B68

CAAGGAGTGAAGAATTTTATTCCCTGCCTCTCAATCTAAACAAATACTTCCCATCTGTGTGATTTAATCAGCATCAGAATCATGAGTTGTCTTGTCCAAATATGTGTTCTCAATAGCTC

>SSH-B69

ACCTTGAAGATTTGACAGATTCTCGATTCCGCTTTAAAAAAGCTATAGCCTTTCCGGGGAATGAAAATCTAGAGACTAGAGTATGCCCGGCTGAAAGGAGAGAAGAAAGAACGGACTTTTGTAGGGAGAAGAGAAGAAGTAAGGCTAACCAACTCGGAATCTGCCTCTGATCGAAGAGTTATCCTATTCTATTATTTATTCCCGCCTGCCCCCTTTGTCTGTCTGGTAAGGAGTTGTTACTGAATCTCCGCCCCTTCCTTCGGTTCGGGAGAATATTCTGTATTCTCGACCAAAGAGGGTATATGTAAAAATCTAGAGCGATCGGGTGAGGGAATACGCAGTAACTCGACCAAAGAACAAAGAAGGGTTCAGAAGTTTAAATAGATAATAAGTGAAGAAAGGAAGTGAAGATTCCTATTCCTCGTTCGAGTGTTCCGTTTTGTAAGAACTCTTAACGTAAGAAATTAAATAAGAGAAGAAGGTTCCCCGTAGACCCTTTCTAGAAGCATTAGCCGGTTGGGAAGTCAATTCCTTGTTGCAAGTCAACTCATTCACCTTTCCCTTACTTTTTCAAGTCAACTACCTTTCTCGTTGGGGCTTACAATTAACGCTTTACCTTTTCTCGGACAAAGGCTGATGCCAGCTAAAGATTGAATCGGAAAGGCATGTGAGGGGTCGGCATTTTCCTCTTTATCTGATGATAAGTTGAGGT

>SSH-B70

ACCTGATGCTTTTAAGAATATCATTTCTATATCTAGTTTGACTAGAAATGGCTATGAATTTCAATTCACAGATGATGTTTGCAATATTTATTTTGGAAATAAATATGTCGGTTCGGGTTTCTGAATGATGGTCTTTATTATTTGGATAATAATGACAAACACAAATTGAATGCAAGTGATATAAAAGAATGCAATGCCATGGTGAAAACCAACTCAAGTTCAAAATATATTTGGCACTTAAGGTTATATCATGTTGTAGAAGATAGGATTGCAAAATTGTAGAAAATGGGGATTTTATCCTCATTGGGCTCTGAGCCTACTCCAACTAGTGAATCTTGCCTTCAGGGCAAAATGACTAGATCACCCTTTGTTGGACAAGGGCTAAGAGCTGAAAATATTTTGGAGCTAATACATAGTGATGTATGTGGTCCATTTATGGAAATGGCTAGAGGCGGTTTTCATTATTTTATTACCTTTACTAATGATAAATTAAGGTTTGGGTATTTGTATTTGATGAAATACAAACATGAATCCTTTGAAAAGTTCAAAGAATTTAAATCTGAAGTAGAAAATCAAACAGGAAAGAATATTAAAGCTCTTCGATCAGATCATGGAGGTGAATATTTGAGTACCTGCCCGGGCGGCCGCTCGACCATTTTAAGCAAAACCTTGATGCTCTGCCTTTACTTGCTGACAAGTTAGGCATTTGGATACAAACTCTGCCACATCTCTTTTCATACCCATCCACCAGTAATGCTCCTTTAGCCCCCTATACATTTTTGTGCCACCAGGGTGCATGGCAAAAGGAGACTCATGTGCTTCCTTCAAAATGATTTGCCTCAAATCCACATCATTAGGAACACATATTCTACCTTGGTGTAACAGTAGACCATCATCTCTGATTGAGAACTCTAGTTTCTTGCCCTGCCGGACTTCTTGCAACAGCTTCTGATACCTTTCATCATTCTGAGCAGCCATTCTGATCTGATCAATCAATACTAGCTGT

>SSH-B71

ACAGGGAAAGCTACTCTTCACCGGAAATAGTAAGAGCCGCGAAAAGGCATTCTCTTGAAAGTTTTCAGTAATCGTCTGCGCGACTCACTAAAGGATTTTCACTTAGCCCAGCCGCTTATCCACCTGCAACCCTCACTCGAATAAAGAGCATTTTCCTCGTCAGAAGGTTTTTCCCTCAACGACTAAAGGATAAGGCTAGGGGACTACAACCCGGATACCCAAGGGGGGGAAGCAGCATACTACTCCCTTATCTTACTCAGACAGAAAGAACACGCTAAGCACTAGAAGAAAAGGAAGAGATAAGCTACTCCTACTAGAAAGGGACCTAGATCAATAGAAGGAAGGAAGGACGACAAGCAAGAAGAAAGAACCAGAAGACTACACCCCAACTCAACCATGCCTCTCTTCCACTTAGAAAAAAGACGAGGGAAAGACGAACATCAGGACAAGGGAGCCTCACTCCTAGAAAGAAGGATGATACCATTCCCTTGTTGACTCAACCCATGACAGGGAGAAAAAAAAGAAAGGAAGAAGAACAAGCACATTAATCAAGCCCTTGCTTCAGTTCTTGGACGACACCAACTCGTCTTTCTTTTGGAATCTTTAGCCGTAAAACATGTATAGGATAAACTCCGAGATTCCGTTAGGGGTTGCTCTGTTTCAAGAGCATCCTTTCAATTTCAGGGACGATTTGGTGACCGGAGTCAGGT

>SSH-B72

ACGGGGAGGAATTGAGATGTCAGATCTTGTATTGATCACATAATTTCTGAATCTTCGGACCGTTCAAATTCTTAGCATTGTCAGTAACGATTTTATTGGGGAGGCCGTGCCGGCAGATGATATTATTTCTGAGGAATTTGAGAAACGTGTTCTGTGTAATGTGGGCATAGGATGTCGCTTCGACCCATTTAGAGAAATAGTCGATAGCTACTAGGATAAATCGGTGTCCATTGGATGCCTTAGGGTTGATGGGACCAATCATGTCGATGCCCTACATTGCGAAAGGCCATGGTGAGACGAGGTTGAACAATTTGTGAGGTGGCACATTTATCGGATCCGCATAGATCTGACACTTATGGCACTTTCGGAAATACTCGACACAATCCTTCTCCATGGTAGTCCAGAAATATCCTCTTCTCATGATTTGCTTAGCCATCATATGCCCATTGGCATGGGTTGCATAGTTTCCCTCATGAGTCTCGAAAAGGATTCTTTTTGCTTCTTTTGTATCCACACATGCCCGTTGGAGCTCCTTTTGTATAGGGTTTCCCCGCTGGGGAAGTATCCCAATGCTAATCGCCGAATCATCCTCTTTTCGTTTTTGCTCGCCTCTGGAGGAAATTCTCTGGTTCTAATGTAGACCAGGATGTCATGATACCAAGGTTTACCATCGGGCTCTTCCTCGATCATGAAGCAGTATGCTGGTTCACTTCTTGCCTTAATCCTCAATACTTGAGTTGTCTGCCCTTCTTCCATTTGAGCCATAACAGCCAGAGTGGCTAAGGCATCTGCAAATTGATTCTTGTCTCGACTAAGGTGAGTGAAAGAGATTTCTTCGAATTTCTTAATCAGCTCCAATAGGT

>SSH-B73

ACCTGATGCTTTTAAGAACATCATTTCTATATCTAGTTTGACTAGAAATAACTATGAATTTCAGTTCACAGATGATGTTTGCAATATTTATTTTGAAAATAAATATGTTGGTTCAGGTTATATGAATGATGGTCTTTATTATTTGGATAATAATGACAAACACAAAATGAATACAAGTGATCTAAATAAATAAAATGCCATGGTGAAAATCAACTCAAGTTCAAAATATATTTGGCACTTAAGGTTATGTCATGTTGCAGAAGATAGGATTGTAAAACTGGAGAAAATGGGGATTCTTTCCTCATTGGGCTCTAAGCCTACTCCAACTTATAAATCTTGCCTTCAGGACAAAATGACTAGATCACCATTTGTTGGGCAAAGGCTAAGGGTTGAAAATATTTTGGAGCTAATGCATAGTGATGTATGTGGTCCATTTAAGGAAATGGCTAGAGGTGATTTTCATTACTTTATTATCTTTACTGATGATAAATCAAGGTTTAGGTATTTGTATTTGATGAAATACAAACATGAATCCTTTGAAAAGTTCAAAGAATTTAAATATAAAGTAGAAACTCAAACAGGAAAGAGTATTAAAGCTCTTCGATCAGATCGTGAAGGTGAATATTTGAGT

>SSH-B74

ACCGTTAGGAACATATACTCTTCCTTCATACATAACCAAGGAGGCAAATTGTAAGGAGTGACAATGACTGGCCAAGATGAATATTGTTGTCCAGACTGACCGAATGGTTGAAACCCATCGGTGCACAGTCCTAGCCTTACATTCCGAACCTCAGCAGCAAATGAAGGATGTGTTTTATTAAAATGCTTCCATGTAGTTGCATCTGAACAATGATGCATTACCCCATCTTCATGGTCATGCTCAGCGTGCCATCTCATTTCTTTCGCTGTTGCATTCGAAGCATATAATCTTTGCAATCTCTGTGTGAGAGGAAAGTAGT

>SSH-B75

ACCTGGTCCAACCTCACAACCCTTCGCGGGTTGGTCAGAAGTCAGTCTTGTTTGGTAAGACGGAATTGGACAAAGAAAAGAGAGTGCTCGACCGGAACAACGGCCAACAAAGACCGTAATTACATAAACCATATCCTCCCCTTATCCGTCTTTAAGGCTTTAAGGCGAACCGTATGGCGGCTGGAAAGAAAGAGACCTCACCTTCTCGTAGATGGTGTCAGTGAGAGCAACTTTTTTATCCCCCGTTGACCTTCTTTTTTAGATAGAATCTTCAATGAGTGGAAAGAAGCAACCTTACTAATAATAAGAAAGGTGCTTGTTGAGTAAGGCCGGCTTTAGAGCCCAAGCCCCTTTAATATGATGAGAAGAAGAGGGGCCGCCCTCTTTTCTTTTCCGCACCAATCCTTATTTACTACTACATCTTTTTTTGGGTGTATAGCTCAGTTGGTAGAGCATTGGGCTTTTAACCTAATGGTCGCAGGTTCAAGTCCTGCTATACCCAAACCTACCTTACACTATACTATTAGTAAGGATGCGTAGTAGCGTTCAAAGATCACTCTTTGGCCTTTAGACTCGCTTCAGCGACTTCGCCCTTGTTGTGACAAGGGGGGTGAGCCGCTCCAAGTCGAAAGCGATAGCGAATCCCGAACTTGCCCACGCGCACCCAAACCGGCCTCAAAAAGCGATCGGAAAGCGAAGCCCTTCCTTCCAATCCAAGCCAGCGAAGCCGCCCCTATATCTAACCACCGCTCCCCCCGATCACATATTAGCTCGATTTTCTTGACGGCTTGAAGGCGAAGCCGCCTATTTCTTAGGGCAAAGGGCCGCTCCCTCCTCCTAACTCCTTCCACTTCTCCCAGGGGCAGGGACTACGGCTTTCCCTTCGGGGAATAACTCCGTGGGATAGAAGAATAGGCTGTTTGCAAGAAAAGGTTTGACAGGAGAGGGAGGAAGACACTCGAGCTAGATAACAGATCTGCGGGGGAAGTGGAAGTCTAAGAGCGAGT

>SSH-B76

ACCGGCATGTCGATAATTGCTTTGGCTTTTGCAGGTCGACTTCGATTTCTCTTCTGCTGACAATGAACCCGAGTAGCTTACCTGACGAAGCACCAAACCTCCTCCCGGCTTTATATGCTTTTGCGGATGAATTCGAAGACTGTATTTCCGCAATCTGTCAAACACTTTCTTCAAATTCACGGTCTTCTTTAGCCCAAGACTTGGCGATCATGTCATCGACATAGACCTCCATTTCCTTGTGTTTCATATCATGAAACAGCGCAGTCATGGCTCGCTGGT

>SSH-B77

ACAAAATTCATTTCAGATGTTCTGCTAGTGCCTGATTTAGATCAAAATTTGTTGAGTGTGGGTCAAATGATGCAAAAAGGCTATTCTTTGACATTCAAAAATAATCACTGTGTAATTGTGGATCCTAATGGCAATGAACTAGCTGATGTGTCTATGGAAAATAGAAGTTTTCCTTTGAATTGGAATTAAATTTCCTTGCATGCTTGTAGGAGCAGTAAATATGATGATGAATCTTTTTTATGGCATAAGAGGCTTGGTCACTATAATTTGGCATCAATCCAGTTTGCTCAAAGGAAGGGACTTGTTAGAGATTTACCTGTTATTGAAGTTTGTTCTGATGTATGTGAAAGTTGTCAGTTAGGAAAGCAACATAGATTGCCATTTCCCATTTCTGGTGCTTGGAGAGCAAATGAAAAATTAGAATTGGTCCATTCAGATGTCTGCGGGCCAATGAGCAATCCTTCACTCAGTGACAACAAGTATTTTATCCTTTTCATAGATGATTTCACAAGAATGACTTGGGTCTACTTTCTCAAGCAAAAGTCTGAAGTGTTTTCTGTCTTCAAAAAATTTAAAGCACTTGTTGAAGCTCAGAGTGGCTGCAAATTGAAGACTCTAAGAACAGATAATGGGAAGGAATATACATCTGGTGATTTCAATCAGTTTTGTGAAGACTTGGGAATTCAACATCAGTTAACGGTTAGCTACTCTCCACAACAGAACGGTGTGTCAGAGAGGAAGAATAGATCAG

>SSH-B78

ACAAAACTAGTAAAACATCGATACATTCAATACATATTTATTTTCTGTTATTTCTTTCTATTTTATTATTTGCACCACTAAGCATTATTGCTTAGCGCGTTGCTTTTGCCACGCGTAGGTTCTGGAGATACTGATCGAGAGCCCAGTAGACCGCAGACTGGGTGAGACCATCCTGCAGCTCTGCATAGTGTCCGTGTCACCTCACCATCTTCAGTGCATTGGTAGGACACTAGGTTTCATTTTGATTTTTGTAGCTAATTTTTACTTTCTCATATGTAATTGAACTTATGAAATGTATTTTGATGTTCATGTAAATAATGAGAATTGTGTTTGTGAATGGAAAAGTGAATATTTATCTGTGATTTATACATGATTATCACATGAGATGAATGATTGAGAAATGAAATTGAAAAATGTTGAGATTTTGATATTGGAGTTTGAGATGATTGAATATGATTATTGGAAGTGTTTTTCACAGGTTCCAAAGAACTGTTTTCTCCATTTTTAGCCGGTACTCTGCCGAATTTTCTATAAAATTTTCGGAACCTCAAATAAATTATAATTTTAATAAAGGGCTCAAATATATTAAATTTCACAAGTTATATTTAAAACCATGATAAAAATTAATTAAGGTATAATAGAGTGTGCCGGTACACCGTGTGACATTGCTTACTCGGGTATACTGT

>SSH-B79

GCTTGTACAAAAACCTCGCAGGTTCAAGAGTGGTTTTAGAGAAGGCACTCAAGCAGCACCAAGGAAGAAGGGAGCAGCAACATCTGCTTAAGGCTAGAGGCTATTGCTGGCATATATCCTGTTACAGATTCAGTCAACAATTATGTGTTGGAACATATTTTTATGAGAACATTGTTTGATGGTTGTTATTCACTTTTGATGCTATTTAGTCTATTGTAGTTCTTTTTGATCTTTAATGGGGATTTGGAGACTAGAGACCATTTAATTACCTGATGTCTCCATTGAAGTGCATCAATTCTATCTTTTGGCTCGACATGAAGTAATATGTGCAATATCTGTTTACATATTTAGCAGTCACTCTGTCATTTGTTGTAACTGTAACTAATTCAGGTTTCAGT

>SSH-B80

ACTGGGATACCAGTAAATCCATATGGTTATTCTTTTTTTGAGTCTAGGAGGAATGCCGCTTTCTTTAAAATAACTCAGTTTCCGAACAGATTTGTCGGATATAGATAGATACCTTAACCAAACCCCCCACCTGTTAAATTCAATAAATTGAAGATTGGGTGGAACTCGTTACTCGCGAGTCCCAACTGAGTGATGTTGTTCAATACAGAAGAAAAATTTCCCAGGGTCATATCATCTCCAAGTATATCAGCGACTCTCTGCGCGTAGGTCCAATTCTGAGGTAGAGCCACAGCTTCCTTTCTCTTTCTTTCTTACGTTATAACTAAGTAAGGCAGGCTTCTAGCACTCGCATCATCTTTGAAATTTTCATTTCTTTACCGGGCTTGGACCATGTCTCCCGAACAATCTCAGT

>SSH-B81

ACCTCTAACAGCATACTCATCATCACTTTCAGATTCGGGTGCCAAATCTGAATTTGAACTTTTATCTTCAATAACTTTTGCAGGCCTCTCTTGAACCTGAGAATCATTCCAATCCCAATAAGCATCCTCATCCACCTTTACATCTCTGCTGACAAAAACCTTCCTTGTTTTAATGTTGAAAACTCTATAACCCTTAGATTGAGAGCTATAGCCAATCAAAATACACTTTTCAGCCTTTTCATCAAGCTTGTCCCTTTTTACATCTGGTATATGAGCATAACAAATGGAGCCAAAGATTTTCAAATGTTTGACTGAAGGCTTAAATCCACTCCAAGCTTCTAAAGGAGTCATACCATTCACAGCCCTTGTAGGAAGTCTATTTTGCAAGTAGACAGCAGTATTCACAGCCTCTGCCAAAAAGAACTTTGGCATTTTCTTCTCAAAAATCATGCACCTAGCCATCTCAAGT

>SSH-B82

ACTCTCCAGACCTACCATAATAGACTTGGTTTCAATTTCAGGTCAAACCGCATGGCGTGATAAGTGAAAAAGAAATCTACCTATGTTGATAGCCCTCTTTCGGAGTCTTTGATTCGATAGTAGTCACCGCTAGAAAGGAAAAAAAGGAAAGCTTTTAACAAAACATCTCTTCAAATCTAAGAGGATTCGCCCGTGAAGGTAGAAAAATTTTCTTGCTTCTCTAAAAGCTGCTATGTTGCCGGGCTAGTCCTTTCTTTTAGGAAGAGTCTTCCCATAGCATAGTCTTGAAACGAAGCCCTTCGAACCTTTTTGCCCTTTCAAATAAAAAAAGCTTTTCGGTGGCTGACTTCTTTGCTTTTTTCTTTTCTCTTGCTTTGTCTGGCAGTTCTCCTTCTACCATGACTGGCCTGAAGCCGGCCTCCTTAACAGTAACAGACATATCTGAAAAGAACATGGCGTCTATCAGAGCTGGTTCTCTGATTTTATTCCTTTCTTGGGGCTATCCCATCTCGTCCAATATCGTAGCGTAGTCTAAGACGCTCAGCCTAGAAATCGACTCAGAGACCTGGCCCTGTCCCAAAATAAAGTTCTTTTCTCTTCTCTTAATAATGGGATAAATCATCAACGGGGGTAGTCCTTTAACCTTTAAACTAAGAAATTCCCCCATGCCTTCTAGGAAGATGCACTTTGATGACTAGGCTAAGCCGCTTCCTATTTTTCCTTCCTATGAATGGATAAATAAAGCTACTGGTATCCCTTCCCAAATCCCGCTCTTTCAAAAAAGATCGTATCGTATATAAGATACCTCCCTTTGCTCTTTTCTTCGATCTGAGCTGAGCCCTCTGAATTGCGGGGAACCCTCTTCTGACTATGAAGATGCCTCGGTTTAGTCTTTCCTGCGCCCTTTTCTTGTCTTTTTGCTATTGGTTGAAGAAGGTCG

>SSH-B83

ACGTTAAGGATTGTTGTTGTCATGCCAATGTATTATTAGCATATTACTACATAGAAAATTGCATACATTATACAACTAAAGTTCATCAAAAGTGCACTATAATTTAGTCACATAACAGAGCAAGCATTGGGGTTCTCATCACTGAATATTTCAGGATCATAAACATACTCTAAGTAAGACTTTGATGACCAATATTCACTTCTTTTGATGTCACTCTTCTTATCTTCCTCTCCCTTTCCTTCTTCACCATCTTTCTTTTC

>SSH-B84

ACTTCAAACTCTTGTGGTTGGTGTATATCTCACACACTTCACCATACAGGTAGTGTCTCCAAATCTTTAGTGCAAAGACTACAGCCGCCATTTTCAAATCATGGGTGGGGTAGTTCTGCTCATGCCTCTTTAGCTGCCTTGAAGCATAAGCCACTACCTTTCCATTCTACATCAAAACACACCATAAGCCAACTCTGGAGGCATCACAGTATACGGTATATCCTTCACCACTCATCGATAATGTTAACACAAGGGCTGTAGTTAGACATTCCTTAAGCTTCTGGAAGCTCTCCTCACAATCAACTGTCCAAATGAATGGAACATTCTTCCAAGTTAACTTAGTTAGGGGAGTTGCTATCCTGGAGAAAATTCTGTAAAAAATGCCTATAGTAGCTAGTTAGGCCTAGAAAACTTTACGCCGTAGTGACTGTTATAGGCCTAAGCCAATCAGTTACCGCCTCAATTTTCTTGGGATCCACTTGAATGCCTTCACTAGAAACCATGTGTCCCAAGAATGAGATGCTTTCTAGCCAAAATTCATATTTTGAAAATTTAGCATATAGCCGGT

>SSH-B85

ACTCTAGAGTCAAAGGGTTTTAAGTTAAGTAGAAAGAAGACAGAATACATGCATTGCAAGTTCAGTGAAGGCCAAACTGGTGATAGGGAAGGAGTTAGTTTGAATGGAGTGGCACTGTCCCAAAGTAATCACTTTAAATATCTAGGCTCAGTCCTTCAAGTAGATGGGGGATGTGAGGAGGATGTTAGTCATAGGATTAAAGCCGGATGGTTGAAGTGGAGACGTGCCACGGGAGTTTTATGTGATCGTAAGATTCCCAATAAATTAAAAGGAAAATTTTACCGTACAGCCATACGACCGGCTATGTTATGTGGTAGTGAGTGTTGGGCACTGAAAGAGTCGTATGTATCTAAGATAAGAGTTGCAGAGATGAGAATATTAAGGTGGATGAGTGGCCATACTAGACTAGATAAAGTCCGTAATGAGAGTATTAGAGAAAAGGTAGGAGTGGTGCCAATTGAAGATAAGTTGAGAGAAGGGAGATTGAGGTGGTTTGGTCATGTGAAGCGTAGACATACGGAGGCTCCAGTTAGACAAGTAGAGCACATTAGATTAGAGGATAGAAAGAAAAAAAAGGGGTAGACTTAAATTGACTTGGATGAGAGTAGT

>SSH-B86

AGAGAAGATGATTCTATCACAGCTGAAAACTGGTTGGACAGAATTGGAAGAGTTTTGAAACAACTTCATTATACTCCAGAGTAGAATTTGGAAGCTGCTGTATCACTACTGCAAGATGATGCATACCAATGGTGGGATACAGTAACTAGTGAGGTGCCACCAGAATAAATAACTTGGGATTTCTTTCTCACTGAGTTCAGAAAGAAGTATGTGGGCAGTGTATACTTGGAAGAGAAAAGAAGGGAATTTATTACCCTACGCCAGAGACAGCTGATAGTGGCTGAATATGAAAGAGAATTTGTCAGACTGAGCCATTATGGGTGGGAAATAGTCCCCAATGAGGCAGAGAGATGCAAGAGATTTGAGGAAGGACTCAATGATAACATTAAAGGAATAATTATAGCCTTGGGGATTACAGAGTTTTCCAAGTTAGTGGAAGCCGCACTGAAGGTTGAAAAAGTTAGAATGAATGAGCAGAACAGAAGGGACAGAAAGCAGAAAAGAGGTTAGGGTCAG

>SSH-B87

ACTACAGTTTAGAAAGATTGGCCAAGTTCTTTATAGATGAGAATATGGACTTCACATCACAGAGAGTGGTCGATGAGGAAGTGGAAAGTCATGCTTCATCTATGATTGAACCAGGAGACAGGAGGGAACCTGCTCCACCAGTACCAGGTGCACCAGCCACATTTCAGCAAATGGTTGAGTTTTCTAGATAGATGGCAGGAGTTTTGCCACCACCACCACCATCTCCACATCAAAAATCTCATATGGAGAGACTCAAGAAGTTTGGAGCAGTGAATTTTTTGGGCAAAAGAGAAGATGATTCTATCACAGCTGAAAACTGGTTGGACAGAATTGGAAGAGTTTTGAAACAACTTCATTATACTCCAGAGTAGAATTTGGAAGCTGCTGTATCACTACTGCAAGATGATGCATACCAATGGTGGGATACAGTAACTAGTGAGGTGCCACCAGAATAAATAACTTGGGATTTCTTTCTCACTGAGTTCAGAAAGAAGTATGTGGGCAGTGTATACTTGGAAGAGAAAAGAAGGGAATTTATTACCCTACGCCAGAGACAGCTGATAGTGGCTGAATATGAAAGAGAATTTGTCAGACTGAGCCATTATGGGTGGGAAATAGTCCCCAATGAGGCAGAGAGATGCAAGAGATTTGAGGAAGGACTCAATGATAACATTAAAGGAATAATTATAGCCTTGGGGATTACAGAGTTTTCCAAGTTAGTGGAAGCCGCACTGAAGGTTGAAAAAGTTAGAATGAATGAGCAGAACAGAAGGGACAGAAAGCAGAAAAGAGGTTAGGGTCAG

>SSH-B88

ACATTACTTGGAGATCAAAATGAAGACAGCGAAATTTACAAACATTATGCCGAATTATGTCAGGGCTCAGCAATGGAACCCTTCCAGCATGATCTGGACAGGGAGGAGCACTATGCCAACATTGTTCGCATGAACATGATAGATGTCATAGATGATGCCACTGTTGAAGCGGAGATGGAAGCGGCTCTGAAGGAGT

>SSH-B89

ACAAAAGTGGACTTTGAGGTGCTAGTAACAAGTCCGCTAGGACAGGAGGTCAAGGTTAATAAAATGTATAGGGATTGTCCTTTGGTGATCCAAGGACATACTTTTCTATTTGATCTCATTGAAATGCCCTTCAGAGATTATGATATCATCTTGGGCATGGATTGGTTAGCTAGGCATCACGCCATTATTGACTGTAGACTGAAGATAGTCACTTTTGGTCTCCTTCAATACAGTGAAGTGGTAATACATGGGGAGAGGCAGCTATTGCCATCAAACATCATTTCGACTGCACTAGCCAGAAAAATGATCAGAAAGGGGTGTGAAGCATACTTAGCACATGTGGTAGACACCAAAGTGGGGAGTCCAGTATTGAGGGACATCCCTACAGTATTTGACTTTCCAGATGTGTTTCCTAATAAATTGTTAGGATTACCTCCAGAAAGAGAGGTGCAGTTTGAGATTAATGTTATGCCTGGTGTGGATCCAATCTCCATAACACCATACAGAATGGCACCAGCAGAGTTGAAGGAGTTGAAGGT

>SSH-B90

GCTTGTACAAAAACCTCGCAGGTTCAAGAGTGGTTTTAGAGAAGGCACTCAAGCAGCACCAAGGAAGAAGGGAGCAGCAACATCTGCTTAAGGCTAGAGGCTATTGCTGGCATATATCCTGTTAC

>SSH-B91

AGTCACATAACAGAGCAAGCATTGGGGTTCTCATCACTGAATATTTCAGGATCATAAACATACTCTAAGTAAGACTTTGATGACCAATATTCACTTCTTTTGATGTCACTCTTCTTATCTTCCTCTCCCTTTCCTTCTTCACCATCTTTCTTTTC

>SSH-B92

ACAAAGAAGAAAGCTTTAAAGTAAAGTTCAGTTAGAGTTCTCCGTTAGGGTGACTCGATAGCTAAAAGTAGGCATTCAGTAAGAAGTAAGTTTGCCTTGTGGCGTGGATCGAAGGAATCAAGAATGTAGAAACCTACCTAGCTTTAGGGGAAAGATCATAGATAGATTATCTGGACTAGATCCTAGaGATCTGGTTAGAGGTTTGCAAGTAGAAAAGTAGGAATAGAGCTTACAGCCCATCATGAGGTCTAATCTCATGAGTCTATCGTGCCAAAGCTACTAATGCTAAGAATGCTTCGCCCTGACTAGCTAGTAAGGGAAGGTAGGTAAGTAAGAGGCGGATCTAGGGAGTCTTTACTTCTTCGACTTCTTATTCTTATTCATAGTTGAGATACTCCACCAGCAAGCAGCACCGGAGTGAATCGTAACGTTTCGATCTGGGGGTCAGGACTCGTCAAGAGACGAGACGTAGCTAATGCTGTTTGATCACCGTCACGAGATTTGTTTGCAGCTACTATAGCTAATCAATCTCCTGCTTTCATTCCGTTTGATTGAAGACTGGAATCAACATGCGATCTGAGAGCGGATCTAGGAATCCCCTGGTAGTGGTAGTTGGGTTGGTGAAATCCGATAATCGATTGGCTTTAGAAAGGCTTTCCAACGAGCCATTGT

>SSH-B93

ACCGTCTTCCCATAACACTACCACGGTGCTAAGGATTTATGCCACGAAGGCATAAATAAACAGGAGTCACCACCCAGGTAATAACCGAGACATATTCAGTGTTTCTTCCAAGGGTCATGGATGGCAACTTACTCCTTGCAGAGTTTCCACAATCATCATTACCATAGCCCAAGGTCTAGGCTCAGGATAGTGGGTACGTAAGGGGAAGGTGTTAGGCACCCCTTACGCCTGATCTAACCTCGTTAATTCCAGTGCCAGTCCTCTACTAATGTTTTTAAAAATATTGTTTATTATTCCTTTATTAAACTTCATCCTAAAAAATGCAATTCTAATCCTACACACGTAAGTAATTCACAAAAGGGTTGTTTACAAATAATTTTTATGCACAAGTAATTCCTATCTATGGGGTTGCTAATTATTTAAATGATATTTATCAAATGACTAAAATTAATTTATTATTGAGAATTTAATTTACAAACAAATTCAATTTCAAATAGCCCTATGTTTCTATTTAACCTAATTAATTAATTTTCATCAAGTTACCCTAAGGATAATTGAACTAAGTTAATTATGT

>SSH-B94

ACATTCTTACAAAATTTACATCAAAAGAAATACAAACTAAACTTTATTACAAACTTCATACAAATTTTTTTACAGGCTGCTCAAGACCCATTTGCACGTCTATACATTTATATGCAATACATACATGAAAAGAAATATTTACAATTAGGGTATAAATTATACCCGAAGACTTTAAGCTGTAGTCCATCCGAACTGGCAGAAAATGAGCAGACTTGGTCAGTCTGTCAACAATGACCCAAACCACATCATGACTCTTCTGTGTCATCGGAAGTCCCATCACAAAATCCATCGTTATTCTCTCCCATTTCCACTCTGGTATTGGTAGTGGATGTAACAACCCAGTGGGT

>SSH-B95

ACAAGACTCAAAAAGGAGAAACCAAGAGGATCTTTGCAAATACCAAGCCTTTCAGACGACAGAAGCATTCTATGCAGATTCAAAATTCATTCTATTTGGAGCGGAGTTGCGAAAGCCTGCTCCTACACCTCCAGATCAAATGGGCTTTCTCGGAGAAAAAAAAATAAGAATATTGAAGCAGGTCGCAGGCTGACCATGAAGAAGACGTATCGCTACATCCCTAGTGAGCAACGTCGGGAAAGTCAAACCATCTTTAATAGATGGAGTCTCTCTCGAAGAGGTTCCATGTCTAAGACTCAAGATCTCTCCAAACGTGGTCATACCAGTTGATGCCACTAAGGCGGAGAGAAGACGGAAGAGGAGACGCCATGCCACTATACCTCCTCCTTTACGTTATCTTCTACGAAAGTTCGATGTTCCCACAACTGTCGACAAGAATGAAGGTCGTGAAGCGTCACTCCCCGTTTTGTTTTGCTGCATGATTCTAAAGTCATTCACTTGATGACGGAAATGGGCTATGACCCTTTCTTTTCAAAGGTGAAGGGCTGTGTAGAGGAAGGGGGGATCGTGCTTGTGCTATCAAGCCGACAAATGACTGTTCTTTTCAATGACTGCTTTCGAGCGGTATTCACGGAAATGACAAATATTGAATGGTTGTAAACAGATTCGCTAGTTGGCACTCTTTCACTAGTGATTTTTAGCTAGGTGCCCTTACCTTTAAATGATGACAGGAGGGGAAGAAGCTCTAACGGAAGCATCCAATCAACCGGGAATCCCACCTTTCATGCTACTGCTGGAGTGGATAATGCCTGTGCCAGCAAACAAACAAAAACTATAAAGAAGAAAAGGCTAGGTAGGTGTCCATGCCACCAACCAACTCCTGAAAGAAAAACAAGAGCTTCGACGGCAAACCACTAGAAGT

>SSH-B96

AAAAAAAAAGGGGGGGGGACAAAAATGTATCATGACCTGAAGTTTTATTATTGGTGGCCTAGTATGAAGAATGACATAGCTAACTATGTGACTAAATGCTTGACATGTTAGCAAGTCAAAGTAGAACATCAAGTTTCAACGGGTTTGCTACAGCCTATACGCATACCTAAATAGAAATGGGATCGGGTCACCATGGATTTTGTAAGTGGTTTACCTCTCACCCAAAAGAAGCATGACGCAGTATGGGTGATAGTGGATAGATTGACGAAGTCAGCACACTTTCTGCCAGTTAGGACTGACTACTCACTGGAGAAGTTAGCAGAATTGTATATCAGTGAGATAGTTAGACTGTATGGAATTCCACTTTCCATTATATCTGATTGAGACCCAAGGTTTACATTGAGATTTTGGAAGAAGTTGCATGAATCTTTGGGT

>SSH-B97

ACCTGATGCTTTTAAGAACATCATTTCTATATCTAGTTTGACTAGAAATGGCTATGAATTTCAGCTCACAGATGATGTTTGCAATATTTATTTTGCAAAATAAATATGTTGGTTCGGGTTATATGAATGATGGTCTTTATTATTTGGATAATAATGACAAACACAAAATAAATGCAAGTGATCTAAATGAATGCAATGCCATGGTGAAAATCAACTCAAGTTCAAAATATATTTGACACTTAAGGTTAGGTCATATTGCAGAAGATAGGATTGCAAAGCTGGGGAAAATGAGGATTTTATCTTCATTGGGTTTTGGACCTACTCCAACTTGTGAATAATGCCTTCAGGGCAAAATGACTAGATCGCCCTTTGTCGGACAAGGACTAAGAGTTGAAAATATTTTGGAGCTAATACATAGTGATGTATGTGGTCCATTTAAAGAAATGGCTAGAGGCAGTTTTCATTATTTTATTACCTTTACTGATGATAAATCAAGGTTTAGGTATTTGTATTTGATGAAATACAGACATGAATCCTTTGAAAAGTTCAAAGAATTTAAATCTGAAGTAGAAAATCAAACAGGAAAGAGTATTAAAGCTCTTCGATCAGATCGTGGAGGTGAATATTTGAGT

>SSH-B98

ACTGGTAGATAGACTTTACAACCCTCACCTCTTTCTGATGGGACTACTCTACTAAGGGTAATCACAGAACAGGCGTAAAAGAGAGGTTTTATTCCTTTGCCGCCATGCCCTTGAGATCTGCTACAGACGTAGGTAAAAGAGAGATCAATACGGACTGAAAATAGTGGAGACTGAACCCTCTACGGAGATGATAGAAGATATAAACCGCTCAATCGATACGATAGAAGAGGAGCTCCGAAGGTTCCTCTCTTCACTCTTTTCGGATGATAAGTCGAGCTAACCTATTCATAAGTCTCACTAGCCGT

>SSH-B99

ACAATATACATTGATAAAATTTACAGCAAATAATTTGGAAATCTGAATACTCATCAAATTCCAAAACTCAAAATATTCATTGCCAATAATGTAAATCATTTGTATGAAATGACTTTGATCACGAAATTCAATTTATCAAATCATGACTAAACATTTAAGGTAGTTCTAACAAAATCAATTATACATAAATCATAACTGAAATCACAATTTTTTACTATGCAAAAACATTCTGTGTCTCAAAAACCATGTTGTATCTCAAAAACCATGCTGTATCTCAAAAATCATGCTGTATCTCAAAAATCATTCTTTATCTCAAAAATCATTCTTTATCTCACTAACTATGCAAGACTAATCCGAAAGGGCCATATTCGAAGTGATTCTAACTCCCTATGGTCGGGGAGGTCGAATCAGATTCTAACTCCCTATGGTCGGGGAGGTCGAATCATCGTATACAGT

>SSH-B100

ACTGCGGGCTTCTTGCGGTTCCCCGGGCGCCTCTTATCTTTCCTTATGTCTTTACTACTTAGAGAGATCACATAATTGCTTCTTTTGTCTGGAAGCCTCTTTCCGGTCTTAGTCTAGTTTTCTACCTTTGCCTCGAGACACGTAGCTCTTTCAATCAACCCCAGCTTGAACTGATAAGAAAGAGGTTTTTTGAATCCCATACCGAGTGCTATCGTATAGTTGATCACTGCTGCATTTATAAGTTATTCGACTCGAAATTGCATATGCATAAGAGAAGAAAGGATAGAAAATAGGTTTACAGTCGTCTCTTTGACTGCAGAGCAAAGCAGAATGAAGACTTAAATAAGTCAAAAAGAGTCTCTGGCTCTTGGGCCGATTCCATTACTTATAGAGCATTCTAGACGCAAATAGAAAGAGAATAGCTCACAGCCAAGAAATTTCATAGAATGACGAAGAGT

>SSH-B101

ACCTTGAAGATTTGACAGATTCTCGATTCCGCTTTAAAAAAGCTATAGCCTTTCCGGGGAATGAAAATCTAGAGACTAGAGTATGCCCGGCTGAAAGGAGAGAAGAAAGAACGGACTTTTGTAGGGAGAAGAGAAGAAGTAAGGCTAACCAACTCGGAATCTGCCTCTGATCGAAGAGTTATCCTATTCTATTATTTATTCCCGCCTGCCCCCTTTGTCTGTCTGGTAAGGAGTTGTTACTGAATCTCCGCCCCTTCCTTCGGTTCGGGAGAATATTCTGTATTCTCGACCAAAGAGGGTATATGTAAAAATCTAGAGCGATCGGGTGAGGGAATACGCAGTAACTCGACCAAAGAACAAAGAAGGGTTCAGAAGTTTAAATAGATAATAAGTGAAGAAAGGAAGTGAAGATTCCTATTCCTCGTTCGAGTGTTCCGTTTTGTAAGAACTCTTAACGTAAGAAATTAAATAAGAGAAGAAGGTTCCCCGTAGACCCTTTCTAGAAGCATTAGCCGGTTGGGAAGTCAATTCCTTGTTGCAAGTCAACTCATTCACCTTTCCCTTACTTTTTCAAGTCAACTACCTTTCTCGTTGGGGCTTACAATTAACGCTTTACCTTTTCTCGGACAAAGGCTGATGCCAGCTAAAGATTG

>SSH-B102

ACAAGATTAAATATTTATTTTTAGGGTTTACTGGTCATTATACAATTCATTGGTCAATGAAAATGTTGACCTTTTCATACATCATGCATAAGTTGATTCTAATATCAACATGTCACAATTTGTAATTCAATATGCTTCTAATATAGTGCAATTTACCATTTTTATAAATTGGCACTCATTGCCAAATTGCTTCAAGCATCATTGTATGTAAATGTCAAATTCTCAATTTTTGTGTGCTAGATTAGTCTAGCTTAAAGTCTTATTTCTCTTGGTTTTTAGCTTCTGGTCAAAAAAGCAAAATTGTAGCTCTATGTCTTATTGCACTCTGGGTAAAATTTCAGGTCATTCTGAGTTGTATAGACCAAGATATGGTCAATTTACTAAAGCTGGATAGATTGCACCTTTAGTGCAAAATTTGGTCAATCTTAGGTCACATTTAGTTCTGGCAGTTTTGGTATCCGAACATGTGCAAGCTATTTGACTTGGTTCTGGCCATTTCAGGGCTTTGGTGTCTTCATAACAATTGT

>SSH-B103

ACCTCAACTTATCATCAGATAAAGAGGAAAATGCCGACCCCTCACATGCCTTTCCGATTCAATCTTTAGCTGGCATCAGCCTTTGTCCGAGAAAAGGTAAAGCGTTAATTGTAAGCCCCAACGAGAAAGGTAGTTGACTTGAAAAAGTAAGGGAAAGGTGAATGAGTTGACTTGCAACAAGGAATTGACTTCCCAACCGGCTAATGCTTCTAGAAAGGGTCTACGGGGAACCTTCTTCTCTTATTTAATTTCTTACGTTAAGAGTTCTTACAAAACGGAACACTCGAACGAGGAATAGGAATCTTCACTTCCTTTCTTCACTTATTATCTATTTAAACTTCTGAACCCTTCTTTGTTCTTTGGTCGAGTTACTGCGTATTCCCTCACCCGATCGCTCTAGATTTTTACATATACCCTCTTTGGTCGAGAATACAGAATATTCTCCCGAACCGAAGGAAGGGGCGGAGATTCAGTAACAACTCCTTACCAGACAGACAAAGGGGGCAGGCGGGAATAAATAATAGAATAGGATAACTCTTCGATCAGAGGCAGATTCCGAGTTGGTTAGCCTTACTTCCTCTCTTCTCCCTACAAAAGTCCGTTCTTTCTTCTCTCCTTTCAGCCGGGCATACTCTAGTCTCTAGATTTTCATTCCCCGGAAAGGCTATAGCTTTTTTAAAGCGGAATCGAGAATCTGTCAAATCTTCAAGGT

>SSH-B104

ACTCTAAGCATCTCTCTTTTAGTCTGCCGGCCTTTGAAATAGCCACGCTTTTCGGGAAGCCCCTTTCTTAAGCTTAAGGGCGAGTGAATGAGAGCGATTTCATCTTTTTCCTCTACTAATACTAAGAGACTGAGTCTCACCGCTTTCCCGGATCTGAGACTGGGGATCCTTCTTACTTAAAAAAATTCTCATTACATTAGGCAATGATAATAGGAAGGACTCTTGTCTAGGAGTCAGAACGATGTGGAAGATCTATATAGAAGCTATTTCCCGGGCTCTTCCTCGCTCACTAGTAAGCGCTTGAAACTCTTCTTTTCATCTCTTCACTCCCCAAAGGGCCACTTTCTTCACTCCAAAGGCATAGGATACTTGCTTTCATTGCTGCAGATAGCAGATATACCTTTTTGTTTTGCCTTACCCACAGACAGAATCAATCCTAGAAATTCGGAAAGGCTTGCTTAGGAAGGGAAGT

>SSH-B105

ACCTATTGTTTATGCAAAAGTCAACATTTTAGTTGACTAATGAAATGAATAGTGACATGAAAACTTGAAATTCAAAAATTGTGAAACTTAAAAATGCAAAATGCCCTAGTAGGCCTAATGTGATTGGTTTGGATAGTTTGGCATGCCAATAGGGTATTGTTTTAGCAGTACTGCGAAAGGAAAGGCTTTATGCCTGTGTTCATGGCTTTATGCCCGTATTCATGGCTTTTATGCCCGTATTCATGGCTTTTATGCCAATTATGTGATATCATGGCTTTTTAGCCATAGTGACTGCATACGTGGTTGACGTTCTGCGTCCCATGGTATGACGGCCCGAGGCACCGCGGTGTCCAGTGCCAACGACCCGTTATCCAGTCCAGTCGTCCAGTATAGGTTACTTGGGCATGGAAAAGTATAACTGTAATTGAACTGATTATTGAAGAAAATACGAAAATTAAGTATCAGGAATGATTACAAAAATACAAAGAAGTTCAAGATCATGAAGAAAATAATAAACGAAATGCATGAAGAAGTTAATATCATAAAACGTAATTAACCATCGACTAAACATTAAGTTGGTAATTATTCAGTTCTTATGAACACAATGGCTAAGAAATTATGATTTTTTTTTATTGGCATATTATTTCTTTCTATTTTATTATTTGCACCACTAAGCTTTATGCTTAGCGCGTCGCTTTTGCAACGCGTAGGTACTGGCCCCGGGGCGGCCGCTCGA

>SSH-B106

ACAGATGGACATATATTACTAATACCAGATAGAGATAGATATTTCACTAGCACCAGTTCTTTACCTATAGCTATAAGCGGGGGAAGCTGAAGCTAGATCTGTCACGTATAAAGGAAAGAAGGACGTTAGATACCTCTTTTTTGTAGATAGGAGACCTCTTTACTCTCATCAATAGTGATGTTGTGATTGTCGTTATTCTATTCCGATGGATTGACTGGTTATAATCTTGACGCCCGAAATAAAAGAAGAGTGGAAGTTGTCTTGAAAGTCAATCTTGACGAAGAGATGAAAGTCGGTTCTCAGTTCCCGAATCAGCTGTTGTTTGTGATCGCGCTGGTAGGAATGCTGCTTTTGTGCCCGGCGATGATTTGGGTTTGGAATGGCCCCTTCTTCTATGCCCGCTATGCCGT

>SSH-B107

ACCGATCTTGATCCGCCATGCATGAATGGCAAACACCTAGAAAGATCCCTGACTCCAGGGAGAAAACTCGATCTTCCTTGACTCTATTACCTGAAAAGCGGTGTCAAAGAGACTCAAAGAAGCCTTCTTTTTATCACGACGTGGATTTTCACCTCCCGTAGAGGCGGATCAATGAGGGGGGATTGGGAAGAGGGGAGCACCATAGACAAGAGCAGATGTCAACCATTTTCGATAACTAGTGGAATGGTTTCCAACTATAGATCTTCTTGTATTGCCCATTTTAAAAACGAGACGAAAGAGAGAGGGACATTAGTGATTCCCCTACTAAC

>SSH-B108

ACGACACGATTTAATTTAATAAAGTAGTAGTTTTGCTACTACAATAGAGTAGTTATGAGTTTTCAGCCTAATCGATCAGAGGAATTTTCTTCAATCTCATCCTCGAACAGGCCGCAATATTTCCTATTTCTCGTCCAAGCCTTTATGAGGAGGTGATTCCCTTTCTTCCACACCGAACAAGCCCCATTGCTCAAAGTGCTCTGAATCCGGTCAACAAATAAGGACCCGGGCTAAGCCTTCGTTCGCAGTCGTAAGTAAAAGATAACAATTAGGCAGTGGAACTTGCCCTAAGTCTAACTAACTAACGAGCCAGAAACTTTTTCTGAGAGTGCTAGACTTGGTATTTCCATTGGTGTGATTTAGTCGGGGTTGTATTTGGAATTGAAGCAGGGCCTCAACCAAAAAGGAGGGAGGTAACTTGGCTTGAATTAACATGGT

>SSH-B109

ACTGGAAAGTTGTAGCTTTTGATCCTGTTGAAAACTATATATATAAATTATGTGCTTTTCAACCTTTTTTTATTAATGAAAGATTTTTCATATTTTTCAGAAT
